# Diversity and Evolutionary Analysis of Iron-Containing (Type-III) Alcohol Dehydrogenases in Eukaryotes

**DOI:** 10.1371/journal.pone.0166851

**Published:** 2016-11-28

**Authors:** Carlos Gaona-López, Adriana Julián-Sánchez, Héctor Riveros-Rosas

**Affiliations:** Departamento de Bioquímica, Facultad de Medicina, Universidad Nacional Autónoma de México (UNAM). Cd. Universitaria, Ciudad de México, México; Russian Academy of Medical Sciences, RUSSIAN FEDERATION

## Abstract

**Background:**

Alcohol dehydrogenase (ADH) activity is widely distributed in the three domains of life. Currently, there are three non-homologous NAD(P)^+^-dependent ADH families reported: Type I ADH comprises Zn-dependent ADHs; type II ADH comprises short-chain ADHs described first in Drosophila; and, type III ADH comprises iron-containing ADHs (FeADHs). These three families arose independently throughout evolution and possess different structures and mechanisms of reaction. While types I and II ADHs have been extensively studied, analyses about the evolution and diversity of (type III) FeADHs have not been published yet. Therefore in this work, a phylogenetic analysis of FeADHs was performed to get insights into the evolution of this protein family, as well as explore the diversity of FeADHs in eukaryotes.

**Principal Findings:**

Results showed that FeADHs from eukaryotes are distributed in thirteen protein subfamilies, eight of them possessing protein sequences distributed in the three domains of life. Interestingly, none of these protein subfamilies possess protein sequences found simultaneously in animals, plants and fungi. Many FeADHs are activated by or contain Fe^2+^, but many others bind to a variety of metals, or even lack of metal cofactor. Animal FeADHs are found in just one protein subfamily, the hydroxyacid-oxoacid transhydrogenase (HOT) subfamily, which includes protein sequences widely distributed in fungi, but not in plants), and in several taxa from lower eukaryotes, bacteria and archaea. Fungi FeADHs are found mainly in two subfamilies: HOT and maleylacetate reductase (MAR), but some can be found also in other three different protein subfamilies. Plant FeADHs are found only in chlorophyta but not in higher plants, and are distributed in three different protein subfamilies.

**Conclusions/Significance:**

FeADHs are a diverse and ancient protein family that shares a common 3D scaffold with a patchy distribution in eukaryotes. The majority of sequenced FeADHs from eukaryotes are distributed in just two subfamilies, HOT and MAR (found mainly in animals and fungi). These two subfamilies comprise almost 85% of all sequenced FeADHs in eukaryotes.

## 1. Introduction

Alcohol dehydrogenase (ADH) activity is widely distributed in numerous phyla, which include organisms belonging to the three domains of life [1,2]. This activity is performed by different enzymes in different organisms. Indeed, there are three non-homologous NAD(P)^+^-dependent ADH families, which arose independently throughout evolution and possess different 3D scaffolds and mechanisms of reaction [3,4]. Type I ADHs were discovered first one hundred years ago by Federico Battelli and Lina Stern [5,6], who made the first preparation of a soluble alcohol dehydrogenase obtained from horse liver. Some years later, Bengt Andersson [7] showed that this enzyme requires the presence of co-zymase or diphosphopyridine nucleotide (actually known as NAD^+^) to be active. In 1937, Erwin Negelein and Hans J. Wulff purified and crystallized an alcohol dehydrogenase from brewers’ yeast [8], and in 1948, Roger K. Bonnichsen and Anders M. Wassen crystallized ADH from horse liver [9]. Few years later, Bert L. Vallee and Frederic L. Hoch showed that zinc is a functional component of the yeast and horse liver ADH [10,11]. Interestingly, horse liver ADH was also the first oligomeric enzyme for which an amino acid sequence [12] and a three-dimensional structure were determined [13].

In contrast, a type II ADH from *Drosophila melanogaster* was purified for the first time in 1968 by William Sofer and Heinrich Ursprung, who showed that this enzyme possesses a lower molecular weight as compared to that of liver and yeast ADHs, as well as a different substrate specificity [14]. Partial primary structure of *Drosophila* alcohol dehydrogenase obtained in 1976 [15] showed extensive differences with liver and yeast ADHs sequences, concluding that large differences exist between the active sites of the *Drosophila* enzyme and the other previously reported ADHs [15,16]. In 1981, Jörnvall and co-workers showed a distant but clear relationship among zinc-containing ADHs and sorbitol dehydrogenase from sheep, and between *Drosophila* ADH and ribitol dehydrogenase from *Klebsiella*, proposing that ADHs can be divided in “long chain” (type I) and “short chain” (type II) alcohol dehydrogenases [17].

A type III ADH was reported for first time by Christopher Wills and co-workers in 1981 [18] who found two ADHs with very different amino acid composition in *Zymomonas mobilis*. This new ADH-II was purified by Robert K. Scopes and described as an iron-activated ADH [19]. The gene which encodes this alcohol dehydrogenase II (*adhB*) from *Zymomonas mobilis* was cloned and sequenced by Tyrrell Conway and co-workers in 1987 [20] showing no homology with all previously sequenced ADHs. However, a few months later, Valerie M. Williamson and Charlotte E. Paquin [21] cloned a reported *ADH4* gene in *Saccharomyces cerevisiae* [22] showing that the amino acid sequence encoded by this *ADH4* gene was homolog to the iron-activated ADH II from *Z*. *mobilis*. A third homolog protein (1,2-propanediol oxidoreductase) encoded by *fucO* gene in *E*. *coli* was identified, allowing Tyrrell Conway and Lonnie O. Ingram to propose that these unusual ADHs comprise a novel (type III) ADH family of enzymes [23]. Later, new protein homologs to the iron-activated alcohol dehydrogenase (FeADH) family displaying different activities were found. Thus, glycerol dehydrogenase (GldA) from *Escherichia coli* [24]; butanol dehydrogenase (BdhA and BdhB) from *Clostridium acetobutylicum* [25]; ethanolamine utilization protein (EutG) from *Salmonella typhimurium* [26]; and, 1,3-propanediol dehydrogenase (DhaT) from *Klebsiella pneumoniae [27]* were all identified as homologs of the FeADH family. Although type III ADHs were initially described only in microorganisms, Yingfeng Deng and co-workers identified and cloned, in 2002, a gene (*ADHFE1*) that encodes an iron-activated ADH in humans [28].

Nowadays, it has been shown that Zn-dependent (type I) ADHs are homologous to several other proteins that comprise the superfamily of medium-chain dehydrogenases/reductases (MDR) [2,29]; concurrently, short-chain (type II) ADHs belong to the superfamily of short-chain dehydrogenases/reductases (SDR), that comprise many different proteins with diverse catalytic and non-catalytic activities [30,31]. Oppositely, Iron-activated (type III) ADHs have not been extensively studied. According to NCBI's conserved domain database [32], iron-dependent ADHs are related to glycerol-1-phosphate dehydrogenases [33,34] and dehydroquinate synthases [34–36].

Several papers have been published analyzing the origin and evolution of Zn-dependent ADHs [37,38] and MDR superfamily [2], as well as the evolution of short-chain ADHs [1] and SDR superfamily [39]. However, analyses about the evolution and diversity of iron-activated (type III) ADHs have not been published yet. Therefore in this work, a phylogenetic analysis of iron-activated ADHs was performed, to get insights into the evolution of this protein family, as well as explore the diversity of iron-dependent ADHs in distinct eukaryotic phyla.

## 2. Methods

Amino acid sequences from eukaryotes belonging to FeADH family were retrieved by BlastP searches at the NCBI site [40] (http://blast.ncbi.nlm.nih.gov/Blast.cgi), or UniProt database [41] (http://www.uniprot.org/). Progressive multiple amino acid sequence alignments were performed with ClustalX version 2 [42] (http://www.clustal.org/clustal2/) using as a guide a structural alignment constructed with the VAST algorithm [43] (http://www.ncbi.nlm.nih.gov/Structure/VAST/vast.shtml) that included all non-redundant Fe-ADHs protein structures deposited in the Protein Data Bank [44] (http://www.rcsb.org/pdb/home/home.do). Amino acid sequence alignments were corrected manually using BioEdit [45] (http://www.mbio.ncsu.edu/bioedit/bioedit.html).

To obtain the smallest unbiased representative sample of protein sequences that are homologous to FeADHs, protein sequence dataset were collected from Pfam version 29.0 [46] based on representative proteomes [47] at 15% co-membership threshold (RP15). As FeADHs possess ca. 400 amino acids, only retrieved protein sequences with more than 200 residues were included in alignments and phylogenetic analyses.

Phylogenetic analyses were conducted using MEGA7 software [48] (http://www.megasoftware.net). Four methods were used to infer phylogenetic relationships: maximum likelihood (ML), maximum parsimony (MP), minimum evolution (ME), and neighborjoining (NJ). The amino acids substitution model described by Le-Gascuel [49], using a discrete Gamma distribution with five categories, was chosen as the best substitution model, since it gave the lowest Bayesian Information Criterion values and corrected Akaike Information Criterion values [50] in MEGA7 [48]. The gamma shape parameter value (+G parameter = 1.1824) was estimated directly from the data with MEGA7. Confidence for the internal branches of the phylogenetic tree, obtained using ML method, was determined through bootstrap analysis (500 replicates each).

Sequence logos were constructed using the WebLogo server (http://weblogo.threeplusone.com/). Each logo consists of stacks of amino acid letters. The ordinate axis of the logos graphs, indicate the stack for each position in the sequence. The height of the letters within the stack indicates the relative frequency of each amino acid at that position [51].

## 3. Results and Discussion

### 3.1. FeADH family definition

Iron-dependent (type-III) ADHs are reported as members of FeADH family in protein databases. However, public protein database use different criteria to sort amino acid sequences into different protein families and superfamilies; therefore, boundaries between related protein families are not necessarily the same. The NCBI's Conserved Domain Database [32] (http://www.ncbi.nlm.nih.gov/cdd/) identify iron-dependent (type-III) ADHs as members of DHQ-FeADH protein superfamily (cd07766), which comprises four related families: i) the dehydroquinate synthase-like family (cd08169), which catalyzes the conversion of 3-deoxy-D-arabino-heptulosonate-7-phosphate (DAHP) to dehydroquinate (DHQ) in the second step of the shikimate pathway; ii) the family of glycerol-1-phosphate dehydrogenase and related proteins (cd08549); iii) the glycerol dehydrogenase-like family (cd08550); and, iv) the iron-containing alcohol dehydrogenase-like family (cd08551). Pfam database [52] (http://pfam.xfam.org/) sorts these proteins into three different protein families: 1) the dehydroquinate synthase family (PF01761); 2) the iron-containing alcohol dehydrogenase family (PF00465); and, 3) the iron-containing alcohol dehydrogenase family 2 (PF13685).

**Fig 1:**
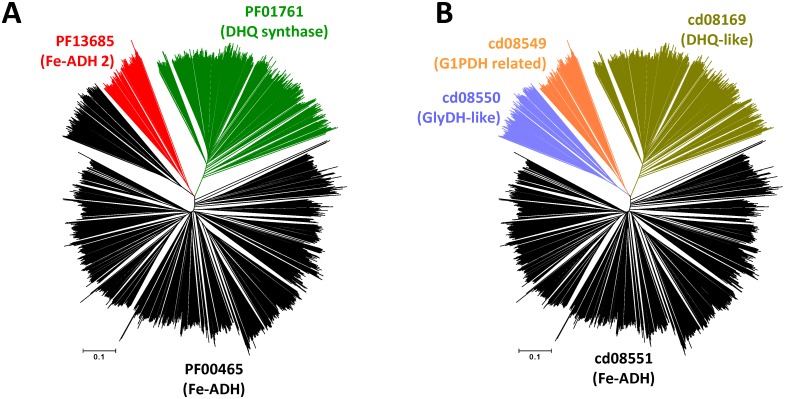
Unrooted tree constructed with protein sequences that possess homology to iron-dependent ADHs. 2459 nonredundant protein sequences were retrieved from Protein Data Bank, Swiss Prot database, NCBI’s Conserved Domain Database, and Pfam database (using RP15 option to allow maximum representation of divergent proteins). Amino acid sequences were ascribed to protein families as considered by Pfam database (A) or NCBI’s Conserved Domain Database (B).

To test the correspondence among the above described protein families, all identified sequences retrieved from the NCBI’s Conserved Domain Database (152 sequences from cd08169 family; 66 sequences from cd08549 family; 118 sequences from cd08550; and 538 sequences from cd08551 family), were aligned with unbiased representative samples of protein sequences (15% co-membership threshold) collected from the Pfam families related with iron-containing ADHs (518 sequences from PF01761 family; 79 sequences from PF13685 family; and 1080 sequences from PF00465 family). Fig 1 shows an unrooted tree illustrating the correspondence between Pfam protein families and NCBI’s Conserved Domain Database families. This figure shows that dehydroquinate synthase-like family (cd08169) shares the same branch as that protein sequences from PF01761 family in the Pfam database. In the same way, the family of glycerol-1-phosphate dehydrogenase and related proteins (cd08549) are located in the same branch as that the iron-containing alcohol dehydrogenase family 2 from Pfam database (PF13685). In contrast, the iron-containing alcohol dehydrogenase family (PF00465 from Pfam database) comprises amino acid sequences that belong to two related protein families in the NCBI’s Conserved Domain Databases: the glycerol dehydrogenase-like family (cd08550); and the iron-containing alcohol dehydrogenase-like family (cd08551). Because glycerol dehydrogenases are reported as Zn-metallo-enzymes not containing iron [53,54], comprise a divergent branch with respect to the other iron-containing alcohol dehydrogenases (Fig 1), and conserve just one of the three conserved histidine residues involved in iron-binding (See 3.7 section), we centered the present analysis to the bona fide iron-dependent alcohol dehydrogenase (FeADH) protein family, as defined in the NCBI’s Conserved Domain Database (cd08551).

### 3.2. (Type III) FeADH family comprises proteins with distinct catalytic activities

Several proteins reported as members from the FeADH family have been characterized exhibiting different catalytic activities. Thus, besides initial reports of iron-containing proteins with ethanol dehydrogenase activity in *Zymomonas mobilis* or *Saccharomyces cerevisiae* [18,21,23,55], other activities have been found: methanol dehydrogenase [56–58], lactaldehyde:propanediol oxidoreductase (lactaldehyde reductase) [23,59], propanol dehydrogenase [60], butanol dehydrogenase [61,62], L-1,3-propanediol dehydrogenase [63–66], maleylacetate reductase [67–71], L-threonine dehydrogenase [72], and hydroxyacid-oxoacid transhydrogenase [73] among others.

### 3.3. (Type-III) FeADH family comprises several protein subfamilies

**Fig 2:**
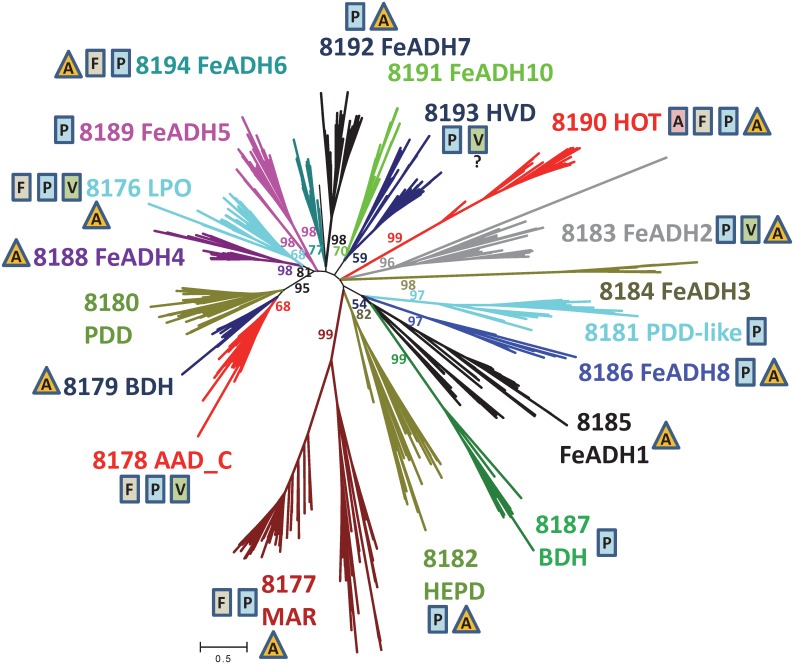
Phylogenetic analysis of 538 Fe-ADH protein sequences retrieved from the NCBI’s Conserved Domain Database (CDD). The unrooted phylogenetic tree was inferred using the Maximum Likelihood method based on the Le-Gascuel model [49]. Branches are colored according to the Conserved Domain Database Fe-ADH subfamily they belong. The tree with the highest log likelihood (-2505413,5328) is shown. Similar trees were obtained with maximum-parsimony, minimum-evolution and neighbour-joining methods. A discrete Gamma distribution was used to model evolutionary rate differences among sites (5 categories (+G, parameter = 0.8682)). The tree is drawn to scale, with branch lengths measured in the number of substitutions per site. There were a total of 783 positions in the final dataset. The proportion of replicate trees in which the associated taxa clustered together in a bootstrap test (500 replicates) is given in color next to selected branches. Rectangles and triangles adjacent to each Fe-ADH subfamily name, indicate the presence of protein sequences from archaea domain (triangles), or eukarya domain (rectangles with A (animals), F (fungi), V (viridiplantae), and P (other eukaryotes) in each subfamily. Protein sequences from bacteria are present in all FeADH subfamilies.

According to NCBI’s Conserved Domain Database, sequences from FeADH protein family (cd08551) are distributed in at least 19 different protein subfamilies (Table 1). To explore the relationships between the different FeADH proteins, an alignment of 538 protein sequences retrieved from the NCBI’s Conserved Domain Database, identified as members of any of the above mentioned 19 protein subfamilies was constructed, and used to perform a phylogenetic analysis. Fig 2 shows a maximum likelihood phylogenetic tree where it can be observed that each of the 19 protein subfamilies proposed by the NCBI’s Conserved Domain Database possesses a good bootstrap support. Blast reciprocal best hits were used as an additional criterion (e.g., [74]) to corroborate that each of these families comprises a putative group of orthologous proteins (data not shown). On the other hand, among the different protein subfamilies comprised by the FeADH family, just a few closely related protein subfamilies showed a good bootstrap support between them. Thus, lactaldehyde:propanediol oxidorectuctase (LPO) subfamily (cd08176) is related to FeADH4 subfamily (cd08188) (81% bootstrap support), and the C-terminal domain of the acetaldehyde-alcohol dehydrogenase two-domain (AAD-C) subfamily (cd08178) is related (95% bootstrap support) to butanol dehydrogenase (BDH) subfamily (cd08179) and propanediol dehydrogenase (PDD) subfamily (cd08180).

**Table 1:** Protein subfamilies that comprise the FeADH family.

CDD Protein Subfamily	Reported activity [properties]/Characterized proteins [accession number]	Reported structure	Phyletic distribution			Reference
			Bacteria	Eukarya	Archaea	
LPO cd08176	**Lactaldehyde:propanediol oxidoreductase (lactaldehyde reductase)** [dimer; NAD^+^-dependent activity]		Yes	Yes	Yes	
	FucO *Escherichia coli* [P0A9S1] [crystallized either with NAD^+^, Fe^2+^, 1,2-propanediol, adenosine diphosphoribose, or Zn^2+^]	2BL4; 2BI4; 1RRM		(Euglenozoa; Heterolobosea; **Fungi**, Ascomycota; **Viridiplantae**, Chlorophyta)		[23,59]
	**L-1,3-Propanediol dehydrogenase** [dimer; NAD^+^-dependent activity]					
	DhaT *Klebsiella pneumoniae* [Q59477][pentamer of dimers; crystallized with Fe^2+^]	3BFJ				[66]
	DhaT *Clostridium pasteurianum* [o30454]	—				[65]
	ADH3 (*dhaT* gene) *Oenococcus oeni* [EKP90059] [dimer; crystallized with Ni^2+^]	4FR2				[64]
	DhaT *Citrobacter freundii* [p45513]	—				[63]
	**Methanol dehydrogenase** [NAD^+^-dependent activity]					
	MDH *Bacillus methanolicus* [P31005] [pentamer of dimers; contains Zn^2+^ and Mg^2+^]	—				[56–58]
	**Ethanol dehydrogenase** [NAD^+^-dependent activity]					
	ADH2 *Zymomonas mobilis* [F8DVL8] [crystallized with NAD^+^, Fe^2+^]	3OX4				[18,23,55]
	ADH4 *Saccharomyces cerevisiae* [P10127][dimer][Zinc activated enzyme]	—				[21,23]
	ADH4 Schizosaccharomyces pombe [Q09669]	—				[119]
	**L-threonine dehydrogenase** [NAD^+^-dependent activity]					
	YiaY *Escherichia coli* [P37686] [enzyme contain both Fe^2+^ and Zn^2+^]	—				[72]
MAR cd08177	**Maleylacetate reductase** [NADH dependent activity] Involved in the degradation of substituted aromatic compounds through the 3-oxoadipate pathway		Yes	Yes	Yes	
	MacA1 Rhodococcus opacus 1CP [O84992]	—		(Haptophyceae; Stramenopiles; **Fungi**, ascomycota, basidiomycota)		[70]
	TfdFI (Reut_D6463) *Cupriavidus necator* JMP134 (previously known as *Ralstonia eutropha* or *Alcaligenes eutrophus*) [P27137]	—				[68,69]
	TfdFII (Reut_D6471) *Cupriavidus necator* JMP134 [P94135]	—				[68,69]
	TcpD (Reut_A1589) *Cupriavidus necator* JMP134 [Q471H8; AAZ60955]	—				[68,69]
	HxqD (Reut_B4129) *Cupriavidus necator* JMP134 [Q46TQ1] [crystallized with NAD^+^]	3JZD				[68,69]
	HqoD (Reut_B4694) *Cupriavidus necator* JMP134 [Q46S41]	—				[68,69]
	MAR (Ncgl2952 locus) *Corynebacterium glutamicum* ATCC 13032 [Q8NL91; NP_602249]	3IV7				[92]
	MAR (Ncgl1112 locus) *Corynebacterium glutamicum* ATCC 13032 [Q8NR93; NP_600385]	—				[92]
	DxnE (Swit_4891 locus) *Sphingomonas wittichii* RW1 [A5VGV4; ABQ71513]	—				[144]
	TftE *Burkholderia cepacia* AC1100 [Q45072]	—				[67,145]
	LinF *Sphingobium japonicum* UT26 (formerly *Sphingomonas paucimobilis* UT26) [Q5W9E3; BAD66863]	—				[146]
	CcaD *Pseudomonas reinekei* MT1 [C6YXH0; ABO61029]	—				[147]
	GraC *Rhizobium* sp. MTP-10005 [A1IIX4; BAF44524] [homodimer]	3W5S				[148]
	MacA (Atu2528 locus) *Agrobacterium fabrum* str. C58 [NP_355474] [crystallized with NAD^+^]	3HL0				Unpublished
	FUM7 *Fusarium verticillioides* [is a gen associated with fumonisin biosynthesis]	—				[133]
AAD-C cd08178	**C-terminal alcohol dehydrogenase domain of the acetaldehyde dehydrogenase-alcohol dehydrogenase bifunctional two-domain protein** [NAD(H) dependent activity]		Yes	Yes	No	
	ADHE Geobacillus thermoglucosidasius NCIMB 11955 [WP_013877698; C-terminal domain, 435–867 aa] [crystallized with Zn^2+^]	3ZDR		(Amoebozoa; Alveolata; Diplomonadida; Cryptophyta; **Viridiplantae**, Chlorophyta; **Fungi**, ascomycota, neocallimastigomycota)		[80,100]
	ADH2 *Entamoeba histolytica* [Q24803]	—				[79–81]
	ADHE *Escherichia coli* K-12 [P0A9Q7] Also possess activity as pyruvate-formatelyase deactivase	—				[82,83]
NADPH-BDH cd08179	**NADPH-dependent butanol dehydrogenase** (*Clostridium saccharobutylicum* and *C*. *beijerinckii* use both ethanol and butanol as substrates)		Yes	No	Yes	
	AdhA *Clostridium beijerinckii* NRRL B592 [AAM18705]	—				[61]
	ADH1 *Clostridium saccharobutylicum*, formerly *C*. *acetobutylicum* [P13604]	—				[62,149]
PDD cd08180	**1,3-propanediol dehydrogenase** [NAD^+^-dependent activity]		Yes	No	No	
	***PduQ*** *Salmonella typhimurium* LT2 [Q9XDN0; NP_460997]	—				[60]
PDD-like cd08181	**Putative 1,3-propanediol dehydrogenase-like** [The enzyme bound NADP^+^]		Yes	Yes	No	
	TM0920 gene of *Thermotoga maritima* [Q9X022; WP_004080642] [crystallized with NADP^+^, Fe^3+^, or Zn^2+^]	1O2D; 1VHD		(Diplomonadida)		[85]
HEPD cd08182	**Hydroxyethylphosphoate dehydrogenase or phosphonoacetaldehyde reductase**. Encoding gene is located inside an operon involved in the biosynthesis of phosphinothricin tripeptide (PTT), an antibiotic used as herbicide.		Yes	Yes^5^	Yes	
	phpC *Streptomyces viridochromogenes* DSM 40736 [AAU00078]	—		(Stramenopiles)		[150,151]
	fomC Streptomyces fradiae [ACG70833]	—				[152,153]
BDH cd08187	**Butanol dehydrogenase / aldehyde reductase** NADP^+^-dependent activity with preference for alcohols longer than C3		Yes	Yes	No	
	YqhD Escherichia coli [Q46856] [crystallized with NADP^+^, Zn^2+^]	1OJ7; 4QGS		(Stramenopiles; Amoebozoa; Parabasalidea)		[86]
	BDH (TM0820 locus) *Thermotoga maritima* MSB8 [NP_228629; Q9WZS7] [crystallized with NADP^+^, Fe^3+^]	1VLJ				Unpublished
HOT cd08190	**Hydroxyacid-oxoacid transhydrogenase**		Yes	Yes	Yes	
	ADHFE1 Homo sapiens [Q8IWW8]	—		(Ichthyosporea; Apusozoa; Stramenopiles; Amoebozoa; Rhizaria; **Fungi**; **Metazoa**)		[28,73,104,107,154]
	ADHFE1 Rattus norvegicus [Q4QQW3]	—				
HVD cd08193	**5-hydroxyvalerate dehydrogenase**		Yes	Yes^4^	No	
	CpnD *Comamonas* sp. NCIMB 9872 [BAC22648]	—		(Haptophyceae; **Viridiplantae**, tracheophyta)		[155]
FeADH1 cd08185	None characterized	—	Yes	No	Yes	
FeADH2 cd08183	None characterized	—	Yes	Yes	Yes^1^	
				(Alveolata; Stramenopiles; Rhodophyta; **Viridiplantae**, Chlorophyta)		
FeADH3 cd08184	**3-deoxy-alpha-D-manno-octulosonate 8-oxidase**. [Catalyzes the first step of the biosynthesis of Kdo8N (8-amino-3,8-dideoxy-D-manno-octulosonate), found in lipopolysaccharides of members of the Shewanella genus]		Yes	No	No	
	KdnB *Shewanella oneidensis* [Q8EEB0]	—				[156]
FeADH4 cd08188	None characterized		Yes	No	Yes	
FeADH5 cd08189	None characterized		Yes	Yes	No	
				(Euglenozoa)		
FeADH6 cd08194	None characterized		Yes	Yes	Yes	
				(Alveolata; Haptophyceae; Rhizaria; Ichthyosporea; **Fungi**, chytridiomycota)		
FeADH8 cd08186	**NADP**^**+**^**-dependent ADH**, [oxidizes a series of primary aliphatic and aromatic alcohols, but shows a higher affinity for aldehyde substrates].		Yes	Yes	Yes	
	*Thermococcus paralvinellae* (formerly T. sp. ES1) [ACK56133]	—		(Diplomonadida)		[87–90]
	Thermococcus sp. AN1 [AAB63011]	—				
	*Thermococcus hydrothermalis* [CAA74334]	—				
FeADH7 cd08192	**Iron-containing alcohol dehydrogenases** probably involved in the linear alkylbenzenesulfonate (LAS) degradation pathway [in *Parvibaculum lavamentivorans* the expression of the gene encoding this enzyme is induced during growth with LAS] ^2^.		Yes	Yes	Yes^3^	
	*Parvibaculum lavamentivorans* [ABS64400]	—		(Haptophyceae; Stramenopiles)		
HHD FeADH10 cd08191	**6-hydroxyhexanoate dehydrogenase**		Yes	No	No	
	ChnD1 Brevibacterium sp. HCU [AAK73161]	—				[157]

^1^ Found in *Lokiarchaeum* sp. GC14_75.

^2^ Personal communication from Dr. David Schleheck, University of Konstanz.

^3^ Found in Thaumarchaeota archeon SCGC AB-539-E09.

^4^ Found in *Posidonia oceanica*, a Mediterranean seagrass.

^5^ Found in *Nannochloropsis gaditana*, an oleaginous microalgae.

### 3.4. Phyletic distribution of FeADHs

**Fig 3:**
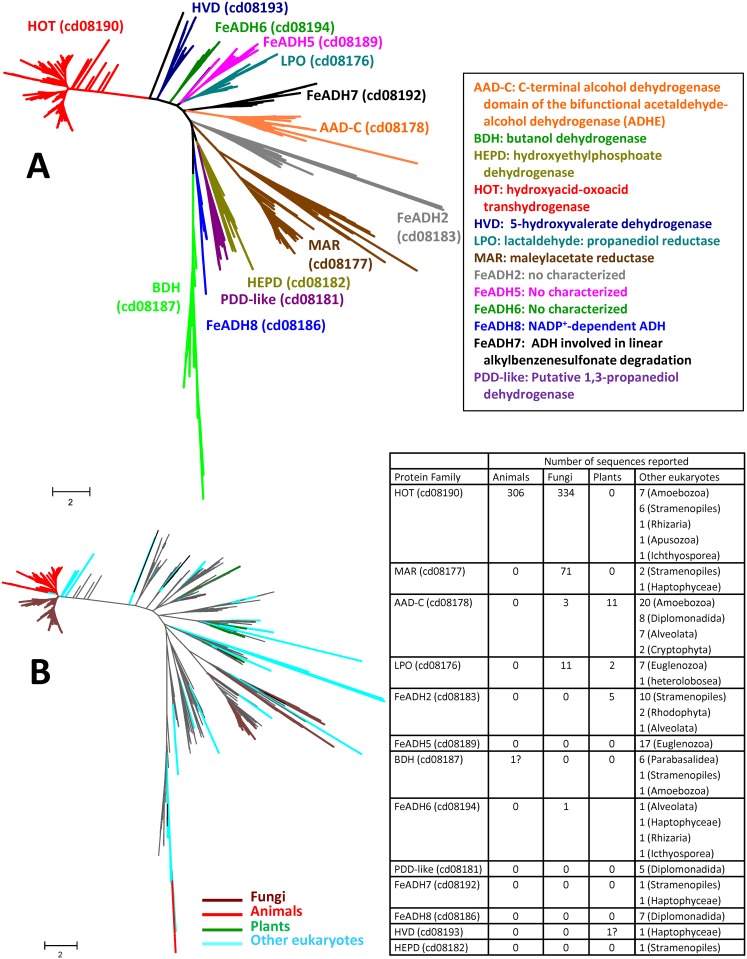
Phylogenetic analysis of 867 Fe-ADH protein sequences from eukaryotes plus 352 non-redundant sequences retrieved from the NCBI’s Conserved Domain Database (CDD). The evolutionary history was inferred using the Maximum Likelihood method based on the Le-Gascuel model [1]. The tree with the highest log likelihood (-3414819.0869) is shown. Initial tree(s) for the heuristic search was/were obtained automatically applying Neighbor-Join and BioNJ algorithms to a matrix of pairwise distances estimated using a JTT model, and then selecting the topology with superior log likelihood value. A discrete Gamma distribution was used to model evolutionary rate differences among sites (5 categories (+G, parameter = 0.4901)). The tree is drawn to scale, with branch lengths measured in the number of substitutions per site. The analysis involved 1219 amino acid sequences. There were a total of 996 positions in the final dataset.

Our results show that the FeADH family members are found in the three domains of life: archaea, bacteria, and eukarya (Table 1). In eukaryotes, FeADHs have a broad distribution and can be found in animals, fungi, plants and many lower eukaryotes. S1 Table provides a complete list of FeADH sequences from eukaryotes identified in this work. Fig 3 shows a phylogenetic tree that comprises all identified FeADH subfamilies that possess proteins from eukaryotes. 656 protein sequences from eukaryotes (from a total of 868 sequences) are members of the HOT subfamily (cd08190). Thus, 75% of all sequenced eukaryotic FeADHs belongs to this subfamily. Indeed, all reported FeADH from animals (306 sequences), and 80% of FeADH found in fungi (334 sequences), belong to this protein subfamily. Other eukaryotes with HOT proteins are amoebozoa like *Acanthamoeba castellani*, *Polysphondylium pallidum*, *Acytostelium subglobosum*, *Dictyostellium discoideum*, *D*. *lacteum*, and *D*. *purpureum*; stramenopiles like *Phaeodactylum tricornutum*, *Thalassiosira oceanica*, *Aphanomyces astaci*, *A*. *invadens*, *Saprolegnia parasitica* and *S*. *diclina*; icthyosporea like *Capsaspora owczarzaki*; Apuzoa like *Thecamonas trahens*; and Rhizaria like the foraminifera *Reticulomyxa filose*. HOT sequences were not found in plants.

In fungi, FeADHs are sorted in two main protein subfamilies: 1) HOT subfamily (cd08190; that includes 80% of fungal protein sequences), is apparently found in all fungal taxa, with exception of some saccharomycetes which include yeast such as *Saccharomyces cerevisiae* and *Kluyveromyces lactis*, and schizosaccharomycetes such as *Schizosaccharomyces pombe*; and 2) MAR subfamily (cd08177), which includes almost 17% of fungal protein sequences, was found mainly in ascomycetes, and basidiomycetes. All reported FeADHs from saccharomycetes and schizosaccharomycetes belong to the LPO subfamily (cd08176) and probably are involved in ethanol metabolism (only the saccharomycete *Geotrichum candidum* was found to possess a FeADH that belong to the HOT subfamily). Three reported fungal sequences belong to the AAD-C (cd08178) subfamily (ADHE from *Togninia minima* (ascomycota), *Neocallimastix frontalis* and *Piromyces* sp. E2 (neocallimastigomycota)). However, the presence of ADHE in eukaryotes has been proposed to result from horizontal gene transfer from different bacteria [75]. In contrast, the FeADH from cd08194 subfamily found in *Gonapodya prolifera* JEL478 (chytridiomycota) is difficult to explain by horizontal gene transfer since the gene encoding this protein possesses 9 exons. Therefore, the origin of this last protein in fungi is uncertain.

FeADHs are absent in superior plants; only green algae (chlrorophyta) possess FeADHs. Interestingly, different classes of green algae possess FeADH that belong to different protein subfamilies (Table 2). Thus, taxa from the chlorophyceae class possess only FeADH that belongs to the bidomain acetaldehyde-alcohol dehydrogenase (AAD-C) subfamily (cd08178). Algae’s from class Trebouxiophyceae possess FeADHs that belongs to AAD-C (cd08178) and lactaldehyde:propanediol oxidoreductase (LPO) subfamily (cd08176); and algae’s from the Class Prasinophyceae possess FeADHs that belong to LPO (cd08176) and one uncharacterized protein subfamily (cd08183). The broad distribution of FeADHs in chlorophyta and its absence in higher plants, suggests that genes encoding FeADHs were lost in the last common ancestor of terrestrial plants. Here, it should be mentioned that a FeADH from 5-hydroxyvalerate dehydrogenase (HVD) subfamily (cd08193) has been reported in the Mediterranean seagrass *Posidonia oceanica*. This protein was sequenced from an isolated mRNA that changed its expression in response to cadmium treatment [76], and showed the highest identity (65%) with FeADHs from *Rhizobium* genera (α-proteobacteria). It is not clear if this reported FeADH in *Posidonia oceanica* might results from horizontal gene transfer from a bacterium, or if it is just the results of bacterial contamination during the total RNA isolation procedure from leaves and apical tips. Because the presence of FeADHs has not been confirmed in any other higher plant, and the absence of additional evidence, its presence in *Posidonia oceanica* should be considered dubious.

**Table 2:** Number of FeADH proteins from plants found in different subfamilies.

Organism	Number of genes			
	cd08178	cd08176	cd08183	cd08193
	AAD-C	LPO	FeADH2	HVD
**Chlorophyta**				
**Class Chlorophyceae**				
*Chlamydomonas reinhardtii* (CC-503 cw92 mt+)	3	0	0	0
*Polytomella* sp. Pringsheim 198.80	1	0	0	0
*Volvox carteri* f. Nagariensis	2	0	0	0
*Gonium pectoral*	2	0	0	0
*Monoraphidium neglectum* SAG 48.87	1			
**Class Trebouxiophyceae**				
*Chlorella variabilis* NC64A	2	0	0	0
*Auxenochlorella protothecoides* 0710 (*Chlorella protothecoides*)	0	1	0	0
**Class Prasinophyceae**				
*Micromonas pusilla* CCMP1545	0	1	1	0
*Micromonas* sp. RCC299 (*Micromonas commoda*)	0	0	1	0
*Ostreococcus tauri*	0	0	1	0
*Ostreococcus lucimarinus* CCE9901	0	0	1	0
*Bathycoccus prasinos*	0	0	1	0
**Streptophyta, Tracheophyta (monocot)**				
*Posidonia oceánica* (Mediterranean seagrass)	0	0	0	1?

It is interesting that fungi and chlorophyta exhibit a patchy distribution of FeADHs, particularly if it is considered that the FeADHs that belong to different protein subfamilies are not functionally equivalent and participate in different metabolic functions.

### 3.5. FeADHs share the same scaffold

**Fig 4:**
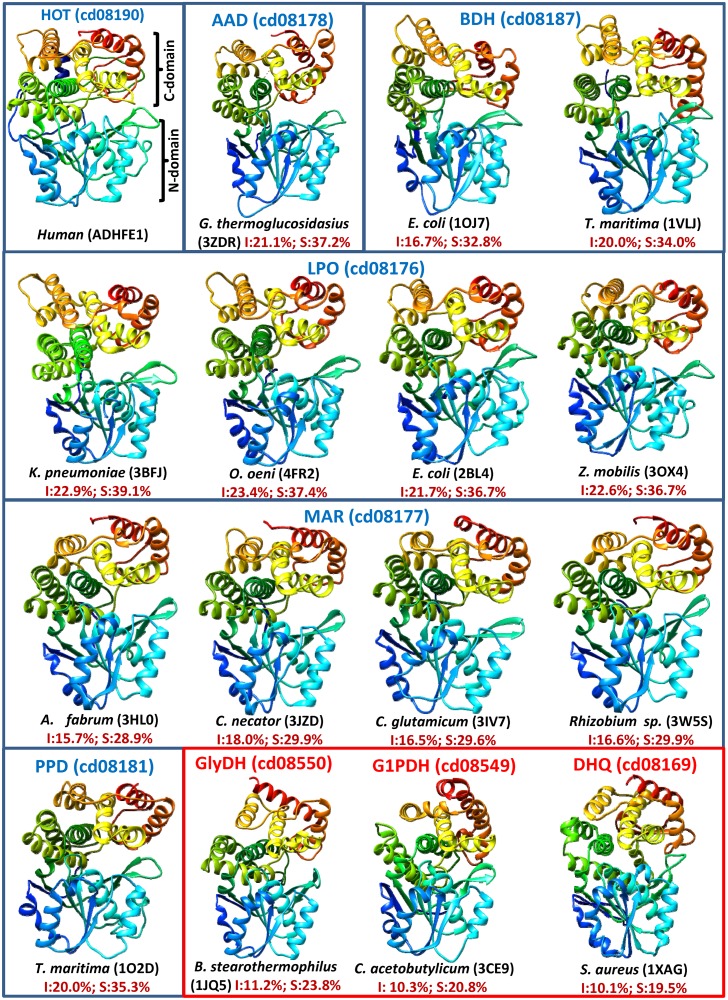
Comparison of the different FeADH proteins with a known three-dimensional structure. These proteins belong to five different FeADH protein subfamilies (sorted inside blue rectangles according to the protein subfamily to which they belong). Below each structure the scientific name of the organisms where the protein is found, as well as the PDB accession number is indicated in parenthesis. In a red rectangle are included representative structures of proteins of homolog protein families that belong to the DHQ-FeADH protein superfamily (cd07766). For reference, a structure prediction (performed with I-TASSER server; [77]) of human ADHFE1 (accession NP_653251), which belongs to HOT subfamily (cd08190), is also included. Numbers in dark red show sequence identity (I) and similarity (S) between human ADHFE1 sequence and the indicated proteins. Secondary structure elements are colored in rainbow successive colors, starting from blue for the N-terminus and ending with red at the C-terminus. Protein structures were drawn using UCSF Chimera version 1.9 [78]

The three-dimensional structures of twelve FeADH proteins have been resolved. These structures are sorted in five different protein subfamilies that belong to the FeADH family (cd08551). All FeADHs have two distinct domains separated by a deep cleft. The α/β N-terminal domain shows a Rossmann-fold structure and contains the coenzyme-binding site. The C-terminal domain is composed of nine α-helices and contains the iron-binding site. In Fig 4 it can be observed that this scaffold is conserved in all members of FeADH protein family as well as in members of related protein families such as glycerol dehydrogenase (GDH) family (cd08550), glycerol-1-phosphate (G1PDH) family (cd08549), and dehydroquinate synthase (DHQ) family (cd08169), which belong to the DHQ-FeADH protein superfamily (cd07766). However, in the DHQ family, the C-terminal domain comprises two or four β-strands in addition to the nine α-helices. The sequence identity among proteins that belong to different FeADH subfamilies is ca. 20% (30–40% sequence similarity), while the sequence identity among proteins that belong to different protein families inside the DHQ-FeADH protein superfamily (cd07766) is ca. 10% (20% similarity). Thus, although protein sequences from different subfamilies are divergent within the FeADH family, Fig 4 shows that they all share a similar scaffold and similar domains.

**Fig 5:**
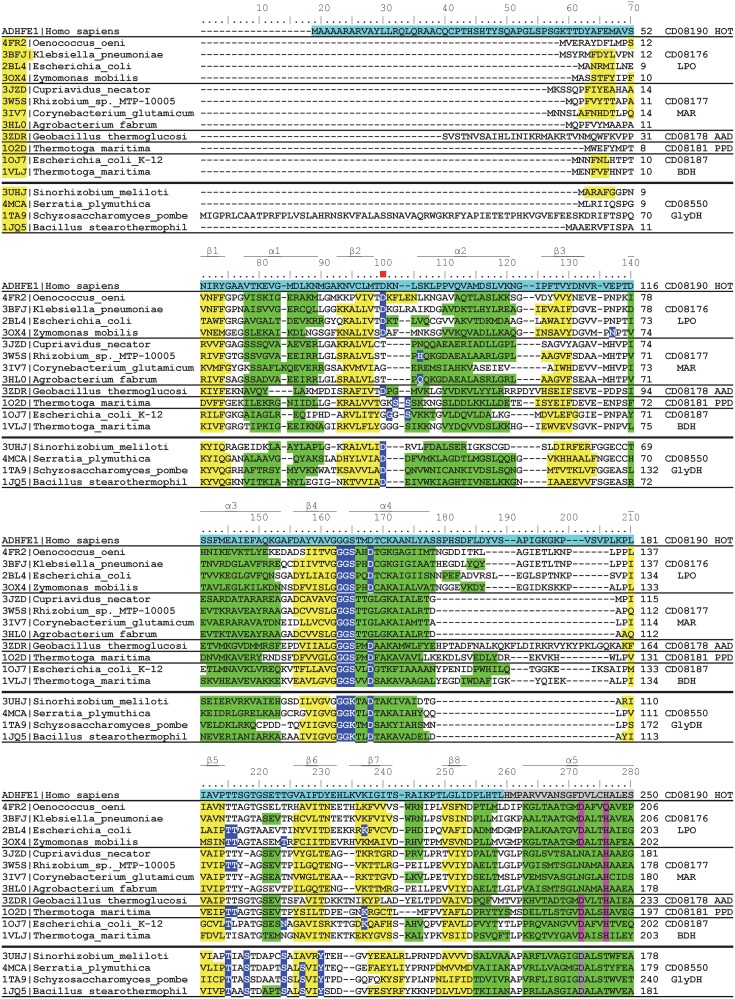
Multiple structure-based sequence alignment of FeADHs with a known 3D structure (residues 1–250 according to human ADHFE1). These proteins belong to five different subfamilies of the FeADH family. For comparison, ADHFE1 sequence from human is included in the alignment, as well as four glycerol dehydrogenase sequences with a known three-dimensional structure. PDB accession number of each sequence is indicated at the left side of alignment, whereas the protein subfamily to which each sequence belongs, is in the right side of the alignment. Conserved β-strands and α-helices for each structure are indicated in yellow and green, respectively. Residue position determinant for coenzyme specificity is indicated with a red square. Residues involved in the binding of Fe atom are highlighted in pink; residues involved in the binding of Zinc atom in glicerol dehydrogenases are highlighted in grey. Amino acid residues from human ADHFE1 sequence, highlighted in blue and grey indicate positions that belong to the N-terminal or C-terminal domains, respectively. The three-dimensional alignment of FeADH structures was performed using the VAST tool at the NCBI’s server [43].

**Fig 6:**
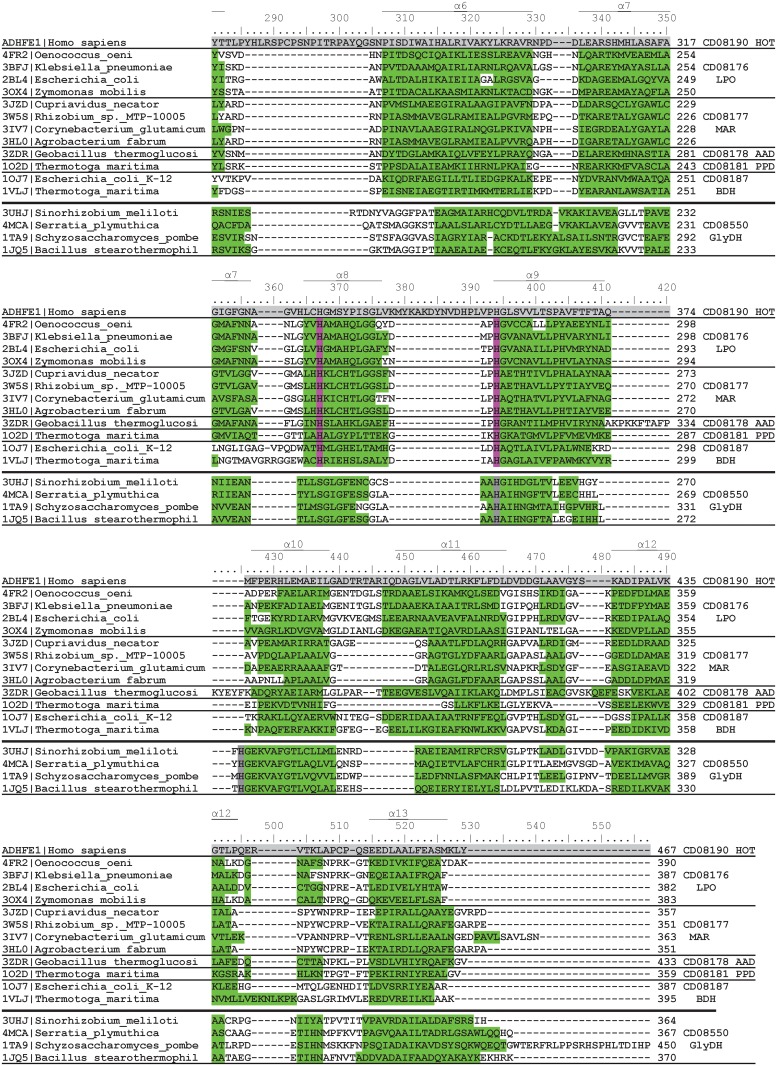
Multiple structure-based sequence alignment of FeADHs with a known 3D structure (residues 251–467 according to human ADHFE1). For additional details see caption of Fig 5.

Figs 5 and 6 show a structure-based multiple sequence alignment of FeADHs with known 3D structure. It can be observed that the twenty-one secondary structures that exhibit the FeADH scaffold are strictly conserved in all FeADHs reported structures (eight β-strains and thirteen α-helices). The N-terminal domain comprises residues 1–229 (human ADHFE1 numbering), while the C-terminal domain comprises residues 230–467 (human ADHFE1 numbering) with the last nine α-helices. Thus, N-terminal domain is involved in binding the coenzyme NAD(P)H; and C-terminal domain possesses the conserved amino acids important for metal ion coordination. For comparative purposes, four structures of glycerol phosphate from the related protein family cd08550 are included in Figs 5 and 6. They show a similar scaffold to FeADHs, but with some differences: the loop located between the α4 helix and the β5 strand is very short in glycerol dehydrogenases; the α6 helix is displaced eight residues, and helices α7 and α8 are joined in one helix. All these differences, together with data from Fig 1, support the idea that glycerol dehydrogenases must be considered as a related protein family separated from *bona fide* FeADH family.

### 3.6. Coenzyme-binding site

As mentioned in the previous section, the N-terminal domain shows a Rossmann-type fold that contains the coenzyme-binding site. Residues involved in coenzyme binding are conserved in all FeADH subfamilies. A GGGS motif (residues 138 to 141 according to human ADHFE1) is conserved in all FeADH subfamilies (Fig 5). This motif interacts with the pyrophosphate group of NAD(P)^+^ and forms a loop that links the β4 strand and the α4 helix.

**Fig 7:**
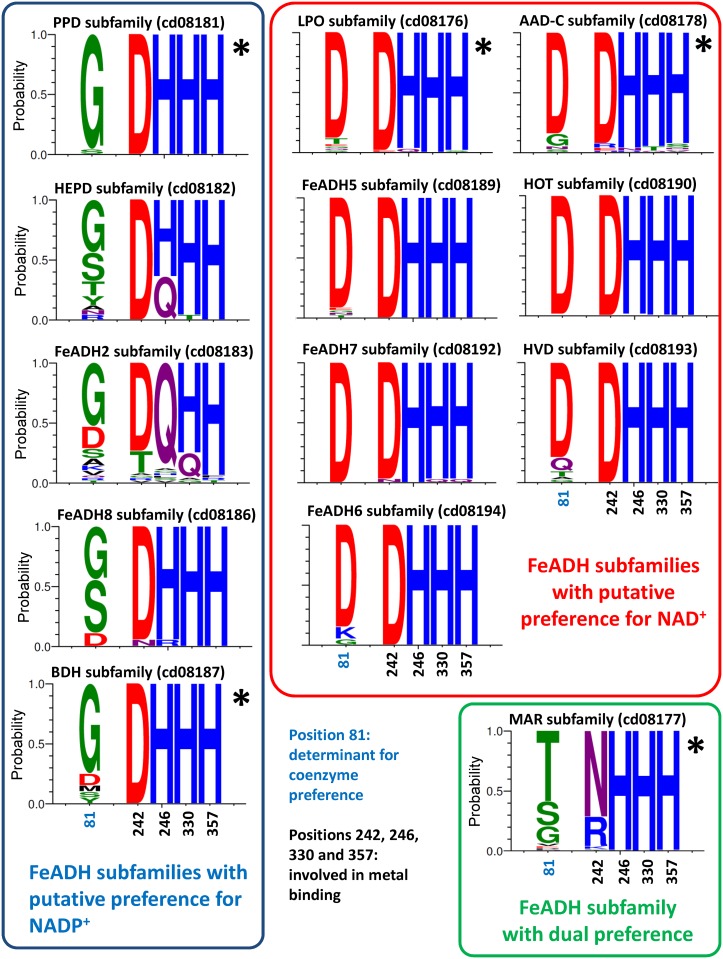
Sequence logos of selected positions in different FeADH subfamilies. The sequences of FeADH family were sorted in subfamilies according to the results of the phylogenetic analysis. Numbering is according to the sequence of human ADHFE1. Residue in position 81 is determinant for coenzyme preference; residues in positions 242, 246, 330 and 357 are involved in metal binding. The amino acid residue coloring scheme was according to their chemical properties: polar (G, S, T, Y, C), green; neutral (Q, N), purple; basic (K, R, H), blue; acidic (D, E), red; and hydrophobic (A, V, L, I, P, M, W, F), black. FeADH subfamilies, whose members putatively use NADP^+^ as coenzyme, are enclosed with a blue box, those that use NAD^+^ as coenzyme, are enclosed with a red box, and those that use both NAD^+^ and NADP^+^, are enclosed in a green box. FeADH subfamilies with experimental support for coenzyme preference are indicated with an asterisk. Sequence logos were made using WebLogo 3 (http://weblogo.threeplusone.com) [51].

Experimental support about coenzyme preference in FeADHs is scarce, but available data show that coenzyme specificity is mainly determined by the nature of the residue at position 81 (human ADHFE1 numbering): Eight different enzymes have been crystallized with their coenzyme as ligand: enzymes that bound NAD^+^ possess aspartate or threonine at position 81, and enzymes that bound NADP^+^ possess glycine at this position. Fig 7 shows the conservation of residues at position 81 as found in a logo analysis.

FeADHs with aspartate at position 81 prefer NAD^+^ because the side-chain of this residue electrostatically and/or sterically repels the 2'-phosphate group of NADP^+^ (the carboxyl group of Asp81 directly interacts with the hydroxyl group at C2 position of the adenine ribose). Examples are FucO from *E*. *coli* [23,59], DhaT from *Klebsiella pneumoniae* [66], ADH II from *Zymomonas mobilis* [18,23,55], MDH from *Bacillus methanolicus* [56–58], ADH4 from *Saccharomyces cerevisiae* [21,23], ADH2 from *Entamoeba histolytica* [79–81], and ADHE from *E*. *coli* [82,83].

In contrast, the shorter side-chain of glycine at position 81 is distant from the ribose and leaves room for binding the 2'-phosphate group of NADP^+^. Thus, enzymes having a residue with a shorter side-chain, can bind both, NAD^+^ with less affinity, or NADP^+^, even sometimes with higher affinity than NAD^+^ [84]. Examples of FeADHs with glycine at position 81 that bind NADP^+^ are: 1,3-propanediol dehydrogenase (TM0920) from *Thermotoga maritima* [85], YqhD from *E*. *coli* [86], butanol dehydrogenase (TM0820) from *Thermotoga maritima* (PDB: 1VLJ), and FeADH from *Thermococcus hydrothermalis*, *T*. *paralvinellae* and *T*. sp. AN1 [87–90].

Serine and threonine are two short-chain residues that have been associated in other NADP^+^-dependent enzymes, as residues that can bind to the 2'-phosphate group of NADP^+^ [84,91]. However, HxqD from *Cupriavidus necator* JMP134, and MacA from *Agrobacterium fabrum*, are two enzymes that belong to the maleylacetate reductase subfamily (cd08177), which were crystallized with NAD^+^ as ligand (PDB: 3JZD and 3HL0), and both possess threonine at position 81. In addition, maleylacetate reductase (Ncgl1112) from *Corynebacterium glutamicum* can use both coenzymes NAD^+^ and NADP^+^ [92], despite having a glycine at position 81. Therefore, although the residue at position 81 is the most important determinant of coenzyme preference, additional residues must be considered. A similar conclusion has been obtained in other NAD(P)-dependent enzymes as for example aldehyde dehydrogenases [84,93,94].

On the other hand, González-Segura et al. [84] analyzed the coenzyme preference of different aldehyde dehydrogenase (ALDH) families and found that coenzyme preference is a variable feature within many ALDH families, consistent with being mainly dependent on a single residue that apparently has no other structural or functional role, and therefore can easily be changed through evolution and selected in response to physiological needs. Considering that residues at position 81 are not conserved in some FeADH subfamilies (e.g., MAR subfamily (cd08177), hydroxyethylphosphoate dehydrogenase (HEPD) subfamily (cd08182), FeADH2 subfamily (cd08183) and FeADH8 subfamily (cd8186)), it is likely that in these subfamilies, coenzyme preference is a variable feature also.

### 3.7. Metal-binding site

The majority of FeADH subfamilies, contain a divalent metal M^2+^, which is tetrahedrally coordinated through an ion dipole interaction with four conserved residues: Asp242, His246, His330, and His357 (according to human ADHFE1 numbering) (Figs 5–7). Interestingly, maleylacetate reductases (MAR subfamily; cd08177) are active in absence of metal ions, and do not have a divalent metal M^2+^ at their active center [69,95–97]; this may be due to the substitution of Asp242 by asparagine or arginine (Figs 5–7), which probably makes that MAR enzymes lose affinity for metal ions [95]. Considering this, members of the uncharacterized subfamily FeADH2 (cd08183), and some members of the HEPD subfamily (cd08182), probably are also functional in absence of divalent metal because Asp242 is replaced by glutamine in these proteins (Fig 7).

In a previous study using site-directed mutagenesis [98], the Fe^2+^-binding participation of His267 from *E*. *coli* FucO was proposed (Tyr334 according to human ADHFE1). However, the crystal structure of *E*. *coli* FucO showed that His267 is not coordinated with Fe^2+^ ions [59]. Recently, Fujii et al., [95] performed an structural comparison between *E*.*coli* FucO and *Rhizobium* sp. MTP-10005 GraC (an enzyme with maleylacetate reductase activity; MAR subfamily; cd08177), and proposed that His267 of FucO correspond to His 243 of GraC, and that both residues could interact with the substrate, and therefore should be involved in catalysis, but not in metal-binding.

It is important to note that despite the members of the FeADH family (cd08551) are described as ‘‘iron-activated” alcohol dehydrogenases, these enzymes are activated by a range of divalent cations, among which, besides iron, we can find others such as zinc, nickel, magnesium, copper, cobalt, or manganese (e.g., [64,86,99,100]). Moreover, in enzymes activated by iron such as *E*. *coli* FucO or *Z*. *mobilis* ADH II (from LPO subfamily; cd08176), iron can be displaced by zinc [59,101]. *E*. *coli* FucO is an interesting example, because although FucO is active only with Fe^2+^ (Zn^2+^ inactivates the enzyme), FucO has *in vitro* a higher affinity for Zn^2+^ than for Fe^2+^ [59].

In the glycerol dehydrogenases, iron is absent but they contain a zinc-atom coordinated by two histidines and one aspartate [102,103]. Thus, only one histidine residue at position 357 (according to human ADHFE1 numbering) is conserved in both families, and is used to coordinate either an iron-atom in FeADHs, or a zinc-atom in glycerol dehydrogenases (see Figs 5 and 6). The differences in metal-binding residues between FeADHs and glycerol dehydrogenases support the idea that these latter proteins are members of a different, but related protein family.

### 3.8. Protein subfamilies that possess FeADHs from eukaryotes

All sequenced eukaryotic FeADHs are sorted in thirteen different protein subfamilies that belong to the FeADH family. Only one FeADH found in *Vitrella brassicaformis* CCMP3155 (Alveolata; Protein accesion number: CEM34088) could not be ascribed to any of above identified protein subfamilies. However, because no Blast reciprocal best hits could be identified for this protein, we propose that this FeADH is just a divergent sequence and not a member of a new FeADH protein subfamily. Four of the FeADH subfamilies found in eukaryotes contain more than 92% of all FeADH sequences identified in these organisms. These subfamilies are:

#### 3.8.1. HOT subfamily (cd08190)

Some proteins of this subfamily have been characterized in mammals and possess activity as hydroxyacid-oxoacid transhydrogenase (HOT), catalyzing the conversion of γ-hydroxybutyrate into succinic semialdehyde in a reaction coupled with the reduction of α-cetoglutarate [73,104,105]. In humans, the gene encoding HOT was denominated *ADHFE1* [28] by the HUGO gene nomenclature committee. In animals, γ-hydroxybutyrate (GHB) is a naturally occurring compound present in micromolar concentration in brain and peripheral tissues [106], and HOT is the most active enzyme that oxidizes GHB [107]. GHB is of interest because it is a natural compound with neuromodulatory properties at central GABAergic synapses [108], is an energy regulator that promotes the release of growth hormone [109], and has been illegally used by athletes as a performance-enhancing drug [110]. Indeed, endogenous GHB metabolism appears to be associated with natural athletic ability [111]. This idea is supported by data that identify *ADHFE1* as an athletic-performance candidate gene, which has been a target for positive selection during 400 years in Thoroughbred horses [112].

The *ADHFE1* gene is expressed mainly in adult liver, kidney, hearth, adipocytes [28,73,113], in hypothalamus and neuroblastoma cells [73], and diverse fetal tissues [28], as well as surface epithelium and crypt top of colorectal mucosa [114]. In contrast, *ADHFE1* transcript is non-detectable in lung, intestine, stomach, seminiferous tubules, muscle and testis [113]. Tae et al. [114] showed that *ADHFE1* expression in colon is higher in well-differentiated tissues than in poorly differentiated tissues, and that colorectal cancer cell lines show a down-regulation of *ADHFE1* mRNA and ADHFE1 protein due to hypermethylation of *ADHFE1* promoter. Therefore, ADHFE1 has an important role in organs with a high metabolic activity, as well as in differentiation and embryonic developmental processes.

Immunocytochemical staining reveals mitochondrial localization for mouse ADHFE1 [113]. Predictions performed with MITOPRED [115] WoLF PSORT [116], and PredSL [117], suggest that all animal ADHFE1s are also mitochondrial (data not shown). These enzymes conserve both the NAD(P)^+^-binding site and an iron-binding motif (see Figs 5–7). ADHFE1 contain a tightly bound cofactor and did not require the addition of NAD(P)^+^ to display catalytic activity [118]. Since ethanol oxidation requires coupling with the reduction of a second molecule such as free NAD(P)^+^, participation of ADHFE1 on ethanol metabolism seems improbable. Furthermore, in human adipocytes exposure to ethanol (1–100 mM) does not modify the *ADHFE1* transcript levels [113], reinforcing the idea that this enzyme is not involved in ethanol metabolism in animals.

With no exceptions, all *ADHFE1* in animals corresponded to a single-copy gene, in spite of several whole genome duplications observed through the evolution of vertebrates. Because all animals have one ADHFE1 that belongs to HOT subfamily, it can be assumed that this protein is performing essential activities in animals.

All HOT proteins possess two insertions: the first is a 19-residue insert (residues 256–274 in human ADHFE1) in a loop located between helices α5 and α6, and the second is a 13-residue insert (residues 342–354 in human ADHFE1) in a loop located between helices α8 and α9 (see Fig 6). This insert is absent in other iron-containing ADH members of FeADH family and even in the glycerol dehydrogenase protein family.

Because members of this protein subfamily are found in the three domains of life (archaea, bacteria and eukarya), this group is probably one of the most ancient protein subfamilies inside the FeADH family. However, the activity performed by this protein in non-animal organisms is unknown.

#### 3.8.2. LPO subfamily (cd08176)

This protein subfamily includes proteins with different catalytic activities (Table 1). Among the reported activities, we found: lactaldehyde:propanediol oxidoreductase (lactaldehyde reductase) [23,59], L-1,3-propanediol dehydrogenase [63–66], methanol dehydrogenase [56–58], alcohol dehydrogenase [18,21,23,55,119], and L-threonine dehydrogenase [72]. In eukarya, proteins that belong to this subfamily have been reported in fungi (saccharomycetes); chlorophyta (*Micromonas pusilla*), euglenozoa and heterolobosea. Of these proteins, only the ADH4 from *Saccharomyces cerevisiae* has been thoroughly characterized [120–122]. *S*. *cerevisiae* possess five alcohol dehydrogenase (Adh) isoenzymes. Cultivation with glucose or ethanol as carbon substrate revealed that ADH1 was the only alcohol dehydrogenase capable of efficiently catalyzing the reduction of acetaldehyde to ethanol [120]. A mutant yeast strain with the sole intact *ADH4* gene was able to grow on glucose but at much slower rates than the wild-type strains, to produce even less ethanol from glucose and was unable to utilize ethanol as carbon source [120]. In contrast, high levels of glycerol and acetaldehyde were observed in this mutant (op. cit.). Because *ADH4* transcription is not observed in strains grown on ethanol, and strains with *ADH4* as the only intact isoenzyme gene, were unable to grow on ethanol [120], it is likely that *ADH4* expression is not related to ethanol consumption, in spite of that the kinetic properties of ADH4 compared with those of other yeast ADHs isoenzymes, showed that ethanol is a suitable substrate for ADH4 [121,122]. Indeed, ethanol and n-propanol are the best substrates for yeast ADH4 [121]. Thus, although the kinetic properties of ADH4 make it suitable for ethanol metabolism, it is possible that this enzyme develops different physiological role(s).

#### 3.8.3. AAD-C subfamily (cd08178)

The C-terminal alcohol dehydrogenase domain of the bifunctional acetaldehyde dehydrogenase-alcohol dehydrogenase bidomain protein corresponds to one of the FeADH subfamilies found in bacteria, fungi, chlorophyta, and in several lower eukaryotes. These bifunctional bidomain enzymes are also known as ADHEs, and are found in many fermentative microorganisms. They catalyze the conversion of an acyl-coenzyme A to an alcohol via an aldehyde intermediate. This is coupled to the oxidation of two NADH molecules to maintain the NAD^+^ pool during fermentative metabolism. ADHE enzymes form large helical multimeric assemblies or ‘spirosomes’ [79,83,123], and consist of an N-terminal acetylating aldehyde dehydrogenase domain, which belongs to ALDH20 protein family of the ALDH superfamily, and a C-terminal alcohol dehydrogenase domain (ADH), which is a member of the AAD-C subfamily of the FeADH family.

ADHEs have been described in many fermentative microorganisms that grow in anaerobic conditions, and it is generally accepted that ADHEs perform the important function of regenerating NAD^+^ from NADH under anaerobic conditions to maintain a continuous flow of glycolysis through alcoholic fermentation [80,124]. The fact that ADHE inhibition in *Entamoeba histolytica* induced a significant accumulation of glycolytic intermediates and lower ATP content [80], as well as the fact that ADHE knockout strains from bacteria and *E*. *histolytica* show the complete abolition of ethanol production and an inability to survive under anaerobic conditions [100,123], strongly support the role of ADHE in ethanol production. Indeed, the expression of the *adhE* gene is greatly increased under anaerobic conditions [81].

Atteia et al. [125] reported the presence of an ADHE in isolated mitochondria from the colorless chlorophyta *Polytomella* sp. Expression at ambient oxygen levels of ADHE in an oxygen-respiring algae extends the occurrence and expression of this enzyme to aerobic eukaryotes growing under aerobic conditions, and suggests that ADHE could be involved in either the maintenance of redox balance (ethanol production), or in ethanol assimilation (producing acetyl-CoA and NADH for respiration); and, depending upon environmental conditions, in both.

Finally, it is interesting to mention that *E*. *coli* ADHE can bind to 70S ribosome, exhibiting a substantial RNA unwinding activity, which can account for the ability of the ribosome to translate through downstream of at least certain mRNA helices [126]. Thus, ADHE can function in *E*. *coli* as a ribosomal regulatory protein, revealing an unexpected moonlighting action that opens the door to find additional functions in other ADHEs.

#### 3.8.4. Maleylacetate reductase (MAR) subfamily (cd08177)

Proteins that belong to this subfamily have been described mainly as maleylacetate reductase (MAR), a key enzyme for degradation of ring-fission products derived from the aerobic microbial degradation of aromatic compounds [71]. They catalyze the NADH- or NADPH-dependent reduction of maleylacetate, at a carbon-carbon double bond, to 3-oxoadipate. We found MAR homologs in the three domains of life (Table 1). In eukaryotes, MARs are present mainly in fungi, including both ascomycetes and basidiomycetes. In lower eukaryotes, MAR homologs were found in Haptophyceae (*Emiliania huxleyi*) and stramenopiles (*Nannochloropsis gaditana*). In fungi, maleylacetate reductases contribute to the catabolism of very common substrates, such as tyrosine, resorcinol, phenol, hydroquinone, gentisate, benzoate, 4-hydroxybenzoate, protocatechuate, vanillate, and even, aromatic pollutants [96,127–132]. In *Fusarium verticilloides*, a MAR homolog gene identified as *FUM7* was found in a cluster of genes involved in fumonisin biosynthesis [133].

Fumonisins are polyketide mycotoxins that can accumulate in plants infected with this fungus and cause several fatal animal diseases, including leukoencephalomalacia in horses, pulmonary edema in swine, cancer in rats and mice, and esophageal cancer in humans [133,134].

*FUM7*-deletion mutants produce fumonisin analogs with an alkene function [135]. This suggests that FUM7 likely catalyzes the reduction of an alkene intermediary of fumonisin biosynthesis, in a reaction similar to that performed by maleylacetate reductases.

## 4. Conclusions

FeADHs belong to an ancient protein family that can be found in the three domains of life. These proteins comprise a complex family with at least 19 different subfamilies with proteins that develop different metabolic functions. Many FeADHs are activated by or contain Fe^2+^, but many others contain other divalent metals as Zn^2+^, or even lack of metal cofactor. In eukarya, the majority of FeADHs belongs to the hydroxyacid oxoacid transhydrogenase (HOT) subfamily (cd08190). Indeed, 100% of FeADHs found in animals, and 80% of FeADHs found in fungi, belong to this protein subfamily. Interestingly, HOT proteins are absent in plants. The rest of FeADHs from eukaryotes shows a patchy phyletic distribution, and are sorted in twelve additional protein families being the more important, the maleylreductase (MAR) subfamily (cd08177) found mainly in fungi, the lactaldehyde:propanediol dehydrogenase (LPO) subfamily (cd08176) and the bidomain aldehyde dehydrogenase-alcohol dehydrogenase (AAD) subfamily (cd08178) found in fungi, chlorophyta and lower eukaryotes. Several protein families with a patchy phyletic distribution have been reported previously, such as glucosamine-6-phosphate isomerase, alcohol dehydrogenase E, hybrid-cluster protein (*prisS*), A-type flavoprotein [75], glycerol-1-phosphate dehydrogenase [136], aerolysin [137], hemerythrin, hemocyainin, tyrosinase [138], phycocyanin-like phycobilisome proteins [139], and circularly permuted RAS-like GTPase domain [140] among others. The patchy distribution of these protein families has been explained mainly through intra- and inter-domain lateral gene transfer events, or gene transfer through endosymbiotic events in lower eukaryotes. Indeed, many genes of bacterial origin in eukaryotes were obtained through endosymbiotic events that generated actual mitochondria and chloroplast. Even more, many microbial eukaryotes obtained additional genes through secondary and tertiary eukaryote-eukaryote endosymbiosis events (e.g., [141–143]). This results in a very complex evolutionary history of lower eukaryotes and open the door to multiple events of gain/loss of protein genes and an extensive horizontal gene transfer. Thus, the scattered distribution of many FeADHs subfamilies in eukaryotes suggests that it is likely its presence/absence in different taxa results from events of lateral gene transfer or endosymbiotic gene transfer.

## Supporting Information

S1 Table Proteins identified in eukaryotes as members of different iron-containing alcohol dehydrogenase subfamilies. (PDF)
