# Correction: Diversity and Evolutionary Analysis of Iron-Containing (Type-III) Alcohol Dehydrogenases in Eukaryotes

**DOI:** 10.1371/journal.pone.0172376

**Published:** 2017-02-13

**Authors:** Carlos Gaona-López, Adriana Julián-Sánchez, Héctor Riveros-Rosas

In the Funding section, the name of the last funder is listed incorrectly. The correct funding information is: This work was supported by Dirección General de Asuntos del Personal Académico, Universidad Nacional Autónoma de México, PAPIIT grants IN216513 and IN225016 to HRR, and Consejo Nacional de Ciencia y Tecnología (México), Ph. D. fellowship No. 233791, and Programa de Doctorado en Ciencias Biomédicas, Universidad Nacional Autónoma de México, to CGL. The funders had no role in study design, data collection and analysis, decision to publish, or preparation of the manuscript.
