# Supplementary material for: Diversity and Evolutionary Analysis of Iron-Containing (Type-III) Alcohol Dehydrogenases in Eukaryotes
Source: PLoS One. 2016 Nov 28;11(11):e0166851. doi: 10.1371/journal.pone.0166851 (PMC5125639; doi:10.1371/journal.pone.0166851)
Supplement: S1 Table — (PDF) [file pone.0166851.s001.pdf]

Carlos Gaona-López, Adriana Julián-Sánchez and Héctor Riveros-Rosas. Diversity and evolutionary analysis of iron-containing (Type-III) alcohol dehydrogenases in eukaryotes.

**Supplementary material.**

**S1 Table.** Proteins identified in eukaryotes as members of different iron-containing alcohol dehydrogenase subfamilies.

| Organism[1]<br>Lineage<br>Chromosome location and exon count | Fe-ADH subfamily (conserved Domain Database)<br>Protein accesion number<br>(Gene locus)<br>Amino acid sequence lenght |
|---|---|
| Animals | |
| Porifera | |
| *Amphimedon queenslandica*<br>Eukaryota; Metazoa; Porifera; Demospongiae; Heteroscleromorpha; Haplosclerida; Niphatidae; Amphimedon.<br>chromosome: Unknown Exon count: 13 | cd08190<br>XP_003384308<br>(LOC100639220)<br>472aa |
| Cnidaria | |
| *Hydra vulgaris*<br>Eukaryota; Metazoa; Cnidaria; Hydrozoa; Hydroidolina; Anthoathecata; Aplanulata; Hydridae; Hydra<br>Chromosome: Unknown Exon count: 8 | cd08190<br>XP_002163284<br>(LOC100201794)<br>472 aa |
| *Nematostella vectensis* (starlet sea anemone)<br>Eukaryota; Metazoa; Cnidaria; Anthozoa; Hexacorallia; Actiniaria; Edwardsiidae; Nematostella<br>Chromosome: Unknown Exon count: 12 | cd08190<br>XP_001640433<br>(NEMVEDRAFT_v1g234082)<br>420 aa |
| *Exaiptasia pallida*<br>Eukaryota; Metazoa; Cnidaria; Anthozoa; Hexacorallia; Actiniaria; Aiptasiidae; Exaiptasia.<br>Chromosome: Exon count: 9 | cd08190<br>KXJ20076<br>(AC249_AIPGENE17932)<br>475 aa |
| *Acropora digitifera*<br>Eukaryota; Metazoa; Cnidaria; Anthozoa; Hexacorallia; Scleractinia; Astrocoeniina; Acroporidae; Acropora.<br>Chromosome: Unknown Exon count: 11 (XP_015748924)<br>Chromosome: Unknown Exon count: 12 (XP_015771800) | cd08190<br>XP_015748924<br>(LOC107328715)<br>410 aa<br><br>cd08190<br>XP_015771800<br>(LOC107350091)<br>361 aa |
| Nematoda | |
| *Ancylostoma duodenale*<br>Eukaryota; Metazoa; Ecdysozoa; Nematoda; Chromadorea; Rhabditida; Strongylida; Ancylostomatoidea; Ancylostomatidae; Ancylostomatinae; Ancylostoma.<br>chromosome: Unknown, Exon count: 8 | cd08190<br>KIH51488<br>(ANCDUO_18426)<br>295aa |
| *Ancylostoma ceylanicum*<br>Eukaryota; Metazoa; Ecdysozoa; Nematoda; Chromadorea; Rhabditida; Strongylida; Ancylostomatoidea; Ancylostomatidae; Ancylostomatinae; Ancylostoma.<br>chromosome: Unknown, Exon count: 12 | cd08190<br>EPB68125<br>(ANCCEY_12781)<br>470aa |
| *Oesophagostomum dentatum*<br>Eukaryota; Metazoa; Ecdysozoa; Nematoda; Chromadorea; Rhabditida; Strongylida; Strongyloidea; Cloacinidae; Oesophagostomum.<br>chromosome: Unknown, Exon count: 7 | cd08190<br>KHJ87540<br>(OESDEN_12684)<br>302aa |
| *Necator americanus*<br>Eukaryota; Metazoa; Ecdysozoa; Nematoda; Chromadorea; Rhabditida; Strongylida; Ancylostomatoidea; Ancylostomatidae; Bunostominae; Necator.<br>chromosome: Unknown, Exon count: 11 | cd08190<br>XP_013299057<br>(NECAME_11412)<br>460aa |
| *Haemonchus contortus* (barber pole worm)<br>Eukaryota; Metazoa; Ecdysozoa; Nematoda; Chromadorea; Rhabditida; Strongylida; Trichostrongyloidea; Haemonchidae; Haemonchinae; Haemonchus.<br>chromosome: Unknown, Exon count: 11 | cd08190<br>CDJ98032<br>HCOI_00128900)<br>443aa |
| *Dictyocaulus viviparus* (bovine lungworm)<br>Eukaryota; Metazoa; Ecdysozoa; Nematoda; Chromadorea; Rhabditida; Strongylida; Trichostrongyloidea; Dictyocaulidae; Dictyocaulinae; Dictyocaulus.<br>chromosome: Unknown, Exon count: 13 | cd08190<br>KJH50934<br>(DICVIV_02895)<br>489aa |
| *Caenorhabditis briggsae*<br>Eukaryota; Metazoa; Ecdysozoa; Nematoda; Chromadorea; Rhabditida; Rhabditoidea; Rhabditidae; Peloderinae; Caenorhabditis.<br>chromosome: II, Exon count: 7 | cd08190<br>XP_002631017<br>(CBG02769)<br>465aa |
| *Caenorhabditis remanei*<br>Eukaryota; Metazoa; Ecdysozoa; Nematoda; Chromadorea; Rhabditida; Rhabditoidea; Rhabditidae; Peloderinae; Caenorhabditis.<br>chromosome: Unknown, Exon count: 7 | cd08190<br>XP_003116873<br>(CRE_02087)<br>465aa |
| *Caenorhabditis elegans*<br>Eukaryota; Metazoa; Ecdysozoa; Nematoda; Chromadorea; Rhabditida; Rhabditoidea; Rhabditidae; Peloderinae; Caenorhabditis.<br>chromosome: II Exon count: 7 | cd08190<br>NP_496764<br>(CELE_Y38F1A.6)<br>465aa |

| Organism[1]<br>Lineage<br>Chromosome location and exon count | Fe-ADH subfamily (conserved Domain Database)<br>Protein accesion number<br>(Gene locus)<br>Amino acid sequence lenght |
|---|---|
| *Caenorhabditis brenneri*<br>Eukaryota; Metazoa; Ecdysozoa; Nematoda; Chromadorea; Rhabditida; Rhabditoidea; Rhabditidae; Peloderinae; Caenorhabditis.<br>chromosome: Unknown, Exon count: 7 | cd08190<br>EGT36044<br>(CAEBREN_19666)<br>465aa |
| *Ascaris suum* (pig roundworm)<br>Eukaryota; Metazoa; Ecdysozoa; Nematoda; Chromadorea; Ascaridida; Ascaridoidea; Ascarididae; Ascaris.<br>chromosome: Unknown, Exon count: 10 | cd08190<br>ERG86848<br>(ASU_01609)<br>472aa |
| *Toxocara canis* (dog roundworm)<br>Eukaryota; Metazoa; Ecdysozoa; Nematoda; Chromadorea; Ascaridida; Ascaridoidea; Toxocaridae; Toxocara.<br>chromosome: Unknown, Exon count: 10 | cd08190<br>KHN73801<br>(Tcan_01903)<br>472aa |
| Arthropoda | |
| *Metaseiulus occidentalis* (western predatory mite)<br>Eukaryota; Metazoa; Ecdysozoa; Arthropoda; Chelicerata; Arachnida; Acari; Parasitiformes; Mesostigmata; Gamasina; Phytoseioidea; Phytoseiidae; Typhlodrominae; Metaseiulus<br>chromosome: Unknown, Exon count: 9 | cd08190<br>XP_003739170<br>(LOC100897235)<br>462 aa |
| *Cerapachys biroi* (clonal raider ant)<br>Eukaryota; Metazoa; Ecdysozoa; Arthropoda; Hexapoda; Insecta; Pterygota; Neoptera; Endopterygota; Hymenoptera; Apocrita; Aculeata; Vespoidea; Formicidae; Cerapachyinae; Cerapachyini; Cerapachys<br>chromosome: Unknown, Exon count: 8 | cd08190<br>XP_011349907<br>(LOC105286565)<br>473 aa |
| *Vollenhovia emeryi*<br>Eukaryota; Metazoa; Ecdysozoa; Arthropoda; Hexapoda; Insecta; Pterygota; Neoptera; Endopterygota; Hymenoptera; Apocrita; Aculeata; Vespoidea; Formicidae; Myrmicinae; Vollenhovia<br>chromosome: Unknown, Exon count: 9 | cd08190<br>XP_011882255<br>(LOC105569976)<br>473 aa |
| *Monomorium pharaonis* (pharaoh ant)<br>Eukaryota; Metazoa; Ecdysozoa; Arthropoda; Hexapoda; Insecta; Pterygota; Neoptera; Endopterygota; Hymenoptera; Apocrita; Aculeata; Vespoidea; Formicidae; Myrmicinae; Monomorium<br>chromosome: Unknown, Exon count: 9 | cd08190<br>XP_012537662<br>(LOC105837435)<br>473 aa |
| *Solenopsis invicta* (red fire ant)<br>Eukaryota; Metazoa; Ecdysozoa; Arthropoda; Hexapoda; Insecta; Pterygota; Neoptera; Endopterygota; Hymenoptera; Apocrita; Aculeata; Vespoidea; Formicidae; Myrmicinae; Solenopsis<br>chromosome: Unknown, Exon count: 8 | cd08190<br>XP_011160547<br>(SINV_01501)<br>473 aa |
| *Pogonomyrmex barbatus* (red harvester ant)<br>Eukaryota; Metazoa; Ecdysozoa; Arthropoda; Hexapoda; Insecta; Pterygota; Neoptera; Endopterygota; Hymenoptera; Apocrita; Aculeata; Vespoidea; Formicidae; Myrmicinae; Pogonomyrmex<br>chromosome: Unknown, Exon count: 8 | cd08190<br>XP_011639925<br>(LOC105428988)<br>418 aa |
| *Wasmannia auropunctata* (little fire ant)<br>Eukaryota; Metazoa; Ecdysozoa; Arthropoda; Hexapoda; Insecta; Pterygota; Neoptera; Endopterygota; Hymenoptera; Apocrita; Aculeata; Vespoidea; Formicidae; Myrmicinae; Wasmannia<br>chromosome: Unknown, Exon count: 8 | cd08190<br>XP_011707387<br>(LOC105462462)<br>473 aa |
| *Harpegnathos saltator* (Jerdon's jumping ant)<br>Eukaryota; Metazoa; Ecdysozoa; Arthropoda; Hexapoda; Insecta; Pterygota; Neoptera; Endopterygota; Hymenoptera; Apocrita; Aculeata; Vespoidea; Formicidae; Ponerinae; Ponerini; Harpegnathos<br>chromosome: Unknown, Exon count: 8 | cd08190<br>XP_011147035<br>(EAI_12458)<br>473 aa |
| *Dinoponera quadriceps*<br>Eukaryota; Metazoa; Ecdysozoa; Arthropoda; Hexapoda; Insecta; Pterygota; Neoptera; Endopterygota; Hymenoptera; Apocrita; Aculeata; Vespoidea; Formicidae; Ponerinae; Ponerini; Dinoponera<br>chromosome: Unknown, Exon count: 9 | cd08190<br>XP_014481918<br>(LOC106748178)<br>474 aa |
| *Linepithema humile* (Argentine ant)<br>Eukaryota; Metazoa; Ecdysozoa; Arthropoda; Hexapoda; Insecta; Pterygota; Neoptera; Endopterygota; Hymenoptera; Apocrita; Aculeata; Vespoidea; Formicidae; Dolichoderinae; Linepithema<br>chromosome: Unknown, Exon count: 8 | cd08190<br>XP_012215053<br>(LOC105667680)<br>473 aa |
| *Camponotus floridanus* (Florida carpenter ant)<br>Eukaryota; Metazoa; Ecdysozoa; Arthropoda; Hexapoda; Insecta; Pterygota; Neoptera; Endopterygota; Hymenoptera; Apocrita; Aculeata; Vespoidea; Formicidae; Formicinae; Camponotus<br>chromosome: Unknown, Exon count: 8 | cd08190<br>XP_011255084<br>(EAG_14414)<br>473 aa |
| *Trachymyrmex zeteki*<br>Eukaryota; Metazoa; Ecdysozoa; Arthropoda; Hexapoda; Insecta; Pterygota; Neoptera; Endopterygota; Hymenoptera; Apocrita; Aculeata; Vespoidea; Formicidae; Myrmicinae; Trachymyrmex<br>chromosome: Unknown, Exon count: 7 | cd08190<br>KYQ55614<br>(ALC60_05536)<br>456 aa |

| Organism[1]<br>Lineage<br>Chromosome location and exon count | Fe-ADH subfamily (conserved Domain Database)<br>Protein accesion number<br>(Gene locus)<br>Amino acid sequence lenght |
|---|---|
| *Atta colombica*<br>Eukaryota; Metazoa; Ecdysozoa; Arthropoda; Hexapoda; Insecta; Pterygota; Neoptera; Endopterygota; Hymenoptera; Apocrita; Aculeata; Vespoidea; Formicidae; Myrmicinae; Atta<br>chromosome: Unknown, Exon count: 22<br>Reported sequence contain 4 different domains (probable sequencing error) | cd08190<br>KYM90944<br>(ALC53_01710)<br>1483 aa (1-445aa) |
| *Acromyrmex echinatior* (Panamanian leafcutter ant)<br>Eukaryota; Metazoa; Ecdysozoa; Arthropoda; Hexapoda; Insecta; Pterygota; Neoptera; Endopterygota; Hymenoptera; Apocrita; Aculeata; Vespoidea; Formicidae; Myrmicinae; Acromyrmex<br>chromosome: Unknown, Exon count: 8 | cd08190<br>XP_011052747<br>(LOC105145091)<br>473 aa |
| *Trachymyrmex septentrionalis*<br>Eukaryota; Metazoa; Ecdysozoa; Arthropoda; Hexapoda; Insecta; Pterygota; Neoptera; Endopterygota; Hymenoptera; Apocrita; Aculeata; Vespoidea; Formicidae; Myrmicinae; Trachymyrmex.<br>chromosome: Unknown, Exon count: 7 | cd08190<br>KYN44264<br>(ALC56_01327)<br>455 aa |
| *Trachymyrmex cornetzi*<br>Eukaryota; Metazoa; Ecdysozoa; Arthropoda; Hexapoda; Insecta; Pterygota; Neoptera; Endopterygota; Hymenoptera; Apocrita; Aculeata; Vespoidea; Formicidae; Myrmicinae; Trachymyrmex.<br>chromosome: Unknown, Exon count: 6 | cd08190<br>KYN19595<br>(ALC57_08071)<br>419 aa |
| *Atta cephalotes*<br>Eukaryota; Metazoa; Ecdysozoa; Arthropoda; Hexapoda; Insecta; Pterygota; Neoptera; Endopterygota; Hymenoptera; Apocrita; Aculeata; Vespoidea; Formicidae; Myrmicinae; Atta<br>chromosome: Unknown, Exon count: 8 | cd08190<br>XP_012060756<br>(LOC105623996)<br>472 aa |
| *Polistes dominula* (European paper wasp)<br>Eukaryota; Metazoa; Ecdysozoa; Arthropoda; Hexapoda; Insecta; Pterygota; Neoptera; Endopterygota; Hymenoptera; Apocrita; Aculeata; Vespoidea; Vespidae; Polistinae; Polistini; Polistes<br>chromosome: Unknown, Exon count: 8 | cd08190<br>XP_015178568<br>(LOC107067499)<br>470 aa |
| *Polistes canadensis*<br>Eukaryota; Metazoa; Ecdysozoa; Arthropoda; Hexapoda; Insecta; Pterygota; Neoptera; Endopterygota; Hymenoptera; Apocrita; Aculeata; Vespoidea; Vespidae; Polistinae; Polistini; Polistes<br>chromosome: Unknown, Exon count: 8 | cd08190<br>XP_014610342<br>(LOC106790130)<br>474 aa |
| *Cyphomyrmex costatus*<br>Eukaryota; Metazoa; Ecdysozoa; Arthropoda; Hexapoda; Insecta; Pterygota; Neoptera; Endopterygota; Hymenoptera; Apocrita; Aculeata; Vespoidea; Formicidae; Myrmicinae; Cyphomyrmex<br>chromosome: Unknown, Exon count: 6 | cd08190<br>KYM97267<br>(ALC62_12000)<br>419 aa |
| *Cephus cinctus* (wheat stem sawfly)<br>Eukaryota; Metazoa; Ecdysozoa; Arthropoda; Hexapoda; Insecta; Pterygota; Neoptera; Endopterygota; Hymenoptera; Cephoidea; Cephidae; Cephus<br>chromosome: Unknown, Exon count: 5 | cd08190<br>XP_015609172<br>(LOC107274489)<br>473 aa |
| *Lasius niger*<br>Eukaryota; Metazoa; Ecdysozoa; Arthropoda; Hexapoda; Insecta; Pterygota; Neoptera; Endopterygota; Hymenoptera; Apocrita; Aculeata; Vespoidea; Formicidae; Formicinae; Lasius; Lasius<br>chromosome: Unknown, Exon count: >3 | cd08190<br>KMR04483<br>(RF55_684)<br>244 aa (fragment) |
| *Microplitis demolitor*<br>Eukaryota; Metazoa; Ecdysozoa; Arthropoda; Hexapoda; Insecta; Pterygota; Neoptera; Endopterygota; Hymenoptera; Apocrita; Ichneumonoidea; Braconidae; Microgastrinae; Microplitis<br>chromosome: Unknown, Exon count: 5 | cd08190<br>XP_008556451<br>(LOC103577546)<br>472 aa |
| *Orussus abietinus*<br>Eukaryota; Metazoa; Ecdysozoa; Arthropoda; Hexapoda; Insecta; Pterygota; Neoptera; Endopterygota; Hymenoptera; Orussoidea; Orussidae; Orussus<br>chromosome: Unknown, Exon count: 6 | cd08190<br>XP_012274945<br>(LOC105696793)<br>244 aa (fragment) |
| *Dufourea novaeangliae*<br>Eukaryota; Metazoa; Ecdysozoa; Arthropoda; Hexapoda; Insecta; Pterygota; Neoptera; Endopterygota; Hymenoptera; Apocrita; Aculeata; Apoidea; Halictidae; Rophitinae; Dufourea<br>chromosome: Unknown, Exon count: 8 | cd08190<br>XP_015430596<br>(LOC107187102)<br>473 aa |
| *Habropoda laboriosa*<br>Eukaryota; Metazoa; Ecdysozoa; Arthropoda; Hexapoda; Insecta; Pterygota; Neoptera; Endopterygota; Hymenoptera; Apocrita; Aculeata; Apoidea; Apidae; Habropoda.<br>chromosome: Unknown, Exon count: 6 | cd08190<br>KOC65611<br>(WH47_01646)<br>419 aa |
| *Megachile rotundata* (alfalfa leafcutting bee)<br>Eukaryota; Metazoa; Ecdysozoa; Arthropoda; Hexapoda; Insecta; Pterygota; Neoptera; Endopterygota; Hymenoptera; Apocrita; Aculeata; Apoidea; Megachilidae; Megachilinae; Megachile<br>chromosome: Unknown, Exon count: 8 | cd08190<br>XP_003701050<br>(LOC100880896)<br>473 aa |

| Organism[1]<br>Lineage<br>Chromosome location and exon count | Fe-ADH subfamily (conserved Domain Database)<br>Protein accesion number<br>(Gene locus)<br>Amino acid sequence lenght |
|---|---|
| *Bombus terrestris* (buff-tailed bumble bee)<br>Eukaryota; Metazoa; Ecdysozoa; Arthropoda; Hexapoda; Insecta; Pterygota; Neoptera; Endopterygota; Hymenoptera; Apocrita; Aculeata; Apoidea; Apidae; Bombus; Bombus<br>chromosome: LG B10, Exon count: 8 | cd08190<br>XP_003398412<br>(LOC100643126)<br>472 aa |
| *Bombus impatiens* (common eastern bumble bee)<br>Eukaryota; Metazoa; Ecdysozoa; Arthropoda; Hexapoda; Insecta; Pterygota; Neoptera; Endopterygota; Hymenoptera; Apocrita; Aculeata; Apoidea; Apidae; Bombus; Pyrobombus<br>chromosome: Unknown, Exon count: 8 | cd08190<br>XP_003489041<br>(LOC100746095)<br>472 aa |
| *Apis dorsata* (giant honey bee)<br>Eukaryota; Metazoa; Ecdysozoa; Arthropoda; Hexapoda; Insecta; Pterygota; Neoptera; Endopterygota; Hymenoptera; Apocrita; Aculeata; Apoidea; Apidae; Apis<br>chromosome: Unknown, Exon count: 9 | cd08190<br>XP_006616811<br>(LOC102671579)<br>471 aa |
| *Apis mellifera* (honey bee)<br>Eukaryota; Metazoa; Ecdysozoa; Arthropoda; Hexapoda; Insecta; Pterygota; Neoptera; Endopterygota; Hymenoptera; Apocrita; Aculeata; Apoidea; Apidae; Apis<br>chromosome: LG10, Exon count: 8 | cd08190<br>XP_624450<br>(LOC552069)<br>475 aa |
| *Apis florea* (little honey bee)<br>Eukaryota; Metazoa; Ecdysozoa; Arthropoda; Hexapoda; Insecta; Pterygota; Neoptera; Endopterygota; Hymenoptera; Apocrita; Aculeata; Apoidea; Apidae; Apis<br>chromosome: Unknown, Exon count: 8<br>(possess two gen copies in tandem inside the same locus) | cd08190<br>XP_012340594<br>XP_003692753<br>XP_012340593<br>(LOC100871730)<br>475 aa |
| *Neodiprion lecontei* (redheaded pine sawfly)<br>Eukaryota; Metazoa; Ecdysozoa; Arthropoda; Hexapoda; Insecta; Pterygota; Neoptera; Endopterygota; Hymenoptera; Tenthredinoidea; Diprionidae; Diprioninae; Neodiprion<br>chromosome: Unknown, Exon count: 5 | cd08190<br>XP_015510345<br>(LOC107217356)<br>474 aa |
| *Athalia rosae* (coleseed sawfly)<br>Eukaryota; Metazoa; Ecdysozoa; Arthropoda; Hexapoda; Insecta; Pterygota; Neoptera; Endopterygota; Hymenoptera; Tenthredinoidea; Tenthredinidae; Allantinae; Athalia<br>chromosome: Unknown, Exon count: 6 | cd08190<br>XP_012256043<br>(LOC105686060)<br>475 aa |
| *Melipona quadrifasciata*<br>Eukaryota; Metazoa; Ecdysozoa; Arthropoda; Hexapoda; Insecta; Pterygota; Neoptera; Endopterygota; Hymenoptera; Apocrita; Aculeata; Apoidea; Apidae; Melipona.<br>Chromosome: Unknown, Exon count: 5 | cd08190<br>KOX72506<br>(WN51_03100)<br>371 aa |
| *Zootermopsis nevadensis*<br>Eukaryota; Metazoa; Ecdysozoa; Arthropoda; Hexapoda; Insecta; Pterygota; Neoptera; Orthopteroidea; Dictyoptera; Isoptera; Termopsidae; Zootermopsis.<br>Chromosome: Unknown, Exon count: 10 | cd08190<br>KDR13466<br>(L798_12575)<br>477 aa |
| *Tribolium castaneum* (red flour beetle)<br>Eukaryota; Metazoa; Ecdysozoa; Arthropoda; Hexapoda; Insecta; Pterygota; Neoptera; Endopterygota; Coleoptera; Polyphaga; Cucujiformia; Tenebrionidae; Tenebrionidae incertae sedis; Tribolium<br>chromosome: LGX Exon count: 7 | cd08190<br>XP_968236<br>(LOC656628)<br>466 aa |
| *Bemisia tabaci*<br>Eukaryota; Metazoa; Ecdysozoa; Arthropoda; Hexapoda; Insecta; Pterygota; Neoptera; Paraneoptera; Hemiptera; Sternorrhyncha; Aleyrodiformes; Aleyrodoidea; Aleyrodidae; Aleyrodinae; Bemisia.<br>Chromosome: Unknown, Exon count: unknown | cd08190<br>AGT15702<br><br>475 aa |
| *Acyrthosiphon pisum* (pea aphid)<br>Eukaryota; Metazoa; Ecdysozoa; Arthropoda; Hexapoda; Insecta; Pterygota; Neoptera; Paraneoptera; Hemiptera; Sternorrhyncha; Aphidiformes; Aphidoidea; Aphididae; Macrosiphini; Acyrthosiphon<br>chromosome: Unknown, Exon count: 2 | cd08190<br>XP_008188055<br>(LOC100169201)<br>463 aa |
| *Diuraphis noxia* (Russian wheat aphid)<br>Eukaryota; Metazoa; Ecdysozoa; Arthropoda; Hexapoda; Insecta; Pterygota; Neoptera; Paraneoptera; Hemiptera; Sternorrhyncha; Aphidiformes; Aphidoidea; Aphididae; Macrosiphini; Diuraphis<br>chromosome: Unknown, Exon count: 2 | cd08190<br>XP_015366664<br>(LOC107163655)<br>463 aa |
| *Papilio machaon* (common yellow swallowtail)<br>Eukaryota; Metazoa; Ecdysozoa; Arthropoda; Hexapoda; Insecta; Pterygota; Neoptera; Endopterygota; Lepidoptera; Glossata; Ditrysia; Papilionoidea; Papilionidae; Papilioninae; Papilio<br>chromosome: Unknown, Exon count: 8 | cd08190<br>XP_014362886<br>(LOC106714366)<br>462 aa |
| *Papilio xuthus* (Asian swallowtail)<br>Eukaryota; Metazoa; Ecdysozoa; Arthropoda; Hexapoda; Insecta; Pterygota; Neoptera; Endopterygota; Lepidoptera; Glossata; Ditrysia; Papilionoidea; Papilionidae; Papilioninae; Papilio<br>chromosome: Unknown, Exon count: 8 | cd08190<br>XP_013176243<br>(LOC106124293)<br>462 aa |

| Organism[1]<br>Lineage<br>Chromosome location and exon count | Fe-ADH subfamily (conserved Domain Database)<br>Protein accesion number<br>(Gene locus)<br>Amino acid sequence lenght |
|---|---|
| *Papilio polytes* (common Mormon)<br>Eukaryota; Metazoa; Ecdysozoa; Arthropoda; Hexapoda; Insecta; Pterygota; Neoptera; Endopterygota; Lepidoptera; Glossata; Ditrysia; Papilionoidea; Papilionidae; Papilioninae; Papilio<br>chromosome: Unknown, Exon count: 9 | cd08190<br>XP_013149477<br>(LOC106111861)<br>462 aa |
| *Danaus plexippus* (monarch butterfly)<br>Eukaryota; Metazoa; Ecdysozoa; Arthropoda; Hexapoda; Insecta; Pterygota; Neoptera; Endopterygota; Lepidoptera; Glossata; Ditrysia; Papilionoidea; Nymphalidae; Danainae; Danaini; Danaina; Danaus; Danaus.<br>Chromosome: Unknown, Exon count: 8 | cd08190<br>EHJ78758<br>(KGM_11849)<br>462 aa |
| *Plutella xylostella* (diamondback moth)<br>Eukaryota; Metazoa; Ecdysozoa; Arthropoda; Hexapoda; Insecta; Pterygota; Neoptera; Endopterygota; Lepidoptera; Glossata; Ditrysia; Yponomeutoidea; Plutellidae; Plutella<br>chromosome: Unknown, Exon count: 10 | cd08190<br>XP_011568694<br>(LOC105398318)<br>437 aa |
| *Amyelois transitella*<br>Eukaryota; Metazoa; Ecdysozoa; Arthropoda; Hexapoda; Insecta; Pterygota; Neoptera; Endopterygota; Lepidoptera; Glossata; Ditrysia; Pyraloidea; Pyralidae; Phycitinae; Amyelois<br>chromosome: Unknown, Exon count: 11 | cd08190<br>XP_013194708<br>(LOC106138185)<br>470 aa |
| *Operophtera brumata* (winter moth)<br>Eukaryota; Metazoa; Ecdysozoa; Arthropoda; Hexapoda; Insecta; Pterygota; Neoptera; Endopterygota; Lepidoptera; Glossata; Ditrysia; Geometroidea; Geometridae; Larentiinae; Operophtera.<br>Chromosome: Unknown, Exon count: >5 | cd08190<br>KOB67867<br>(OBRU01_17854)<br>244 aa (fragment) |
| *Bombyx mori* (domestic silkworm)<br>Eukaryota; Metazoa; Ecdysozoa; Arthropoda; Hexapoda; Insecta; Pterygota; Neoptera; Endopterygota; Lepidoptera; Glossata; Ditrysia; Bombycoidea; Bombycidae; Bombycinae; Bombyx<br>chromosome: Unknown, Exon count: 7 | cd08190<br>XP_004932439<br>(LOC101738633)<br>462 aa |
| *Anopheles darlingi*<br>Eukaryota; Metazoa; Ecdysozoa; Arthropoda; Hexapoda; Insecta; Pterygota; Neoptera; Endopterygota; Diptera; Nematocera; Culicoidea; Culicidae; Anophelinae; Anopheles.<br>Chromosome: Unknown, Exon count: 4 | cd08190<br>ETN60619<br>(AND_007754)<br>465 aa |
| *Anopheles gambiae* str. PEST<br>Eukaryota; Metazoa; Arthropoda; Hexapoda; Insecta; Pterygota; Neoptera; Endopterygota; Diptera; Nematocera; Culicoidea; Culicidae; Anophelinae; Anopheles<br>chromosome: 2L Exon count: 4 | cd08190<br>XP_316676<br>(AgaP_AGAP006646)<br>464 aa |
| *Anopheles sinensis*<br>Eukaryota; Metazoa; Ecdysozoa; Arthropoda; Hexapoda; Insecta; Pterygota; Neoptera; Endopterygota; Diptera; Nematocera; Culicoidea; Culicidae; Anophelinae; Anopheles.<br>Chromosome: Unknown, Exon count: 6<br>Reported sequence contain 2 different domains (probable sequencing error) | cd08190<br>KFB51377<br>(ZHAS_00019665)<br>777 aa |
| *Culex quinquefasciatus* (southern house mosquito)<br>Eukaryota; Metazoa; Ecdysozoa; Arthropoda; Hexapoda; Insecta; Pterygota; Neoptera; Endopterygota; Diptera; Nematocera; Culicoidea; Culicidae; Culicinae; Culicini; Culex; Culex<br>Chromosome: Un Exon count: 4 | cd08190<br>XP_001847094<br>(CpipJ_CPIJ005432)<br>462 aa |
| *Aedes aegypti* (yellow fever mosquito)<br>Eukaryota; Metazoa; Ecdysozoa; Arthropoda; Hexapoda; Insecta; Pterygota; Neoptera; Endopterygota; Diptera; Nematocera; Culicoidea; Culicidae; Culicinae; Aedini; Aedes; Stegomyia<br>Chromosome: 2 Exon count: 5 | cd08190<br>XP_001664064<br>(AaeL_AAEL003729)<br>462 aa |
| *Aedes albopictus* (Asian tiger mosquito)<br>Eukaryota; Metazoa; Ecdysozoa; Arthropoda; Hexapoda; Insecta; Pterygota; Neoptera; Endopterygota; Diptera; Nematocera; Culicoidea; Culicidae; Culicinae; Aedini; Aedes; Stegomyia.<br>Chromosome: Unknown, Exon count: 3 | cd08190<br>KXJ73055<br>(RP20_CCG016645)<br>419 aa |
| *Musca domestica* (house fly)<br>Eukaryota; Metazoa; Ecdysozoa; Arthropoda; Hexapoda; Insecta; Pterygota; Neoptera; Endopterygota; Diptera; Brachycera; Muscomorpha; Muscoidea; Muscidae; Musca.<br>Chromosome: Unknown, Exon count: 3 | cd08190<br>XP_005181246<br>(LOC101898360)<br>462 aa |
| *Stomoxys calcitrans* (stable fly)<br>Eukaryota; Metazoa; Ecdysozoa; Arthropoda; Hexapoda; Insecta; Pterygota; Neoptera; Endopterygota; Diptera; Brachycera; Muscomorpha; Muscoidea; Muscidae; Stomoxys.<br>Chromosome:Unknown, Exon count: 2 | cd08190<br>XP_013104265<br>(LOC106084871)<br>464 aa |
| *Lucilia cuprina* (Australian sheep blowfly)<br>Eukaryota; Metazoa; Ecdysozoa; Arthropoda; Hexapoda; Insecta; Pterygota; Neoptera; Endopterygota; Diptera; Brachycera; Muscomorpha; Oestroidea; Calliphoridae; Luciliinae; Lucilia.<br>Chromosome: Unknown, Exon count: 5 | cd08190<br>KNC23008<br>(FF38_07943)<br>464 aa |

| Organism[1]<br>Lineage<br>Chromosome location and exon count | Fe-ADH subfamily (conserved Domain Database)<br>Protein accesion number<br>(Gene locus)<br>Amino acid sequence lenght |
|---|---|
| *Ceratitis capitata* (Mediterranean fruit fly)<br>Eukaryota; Metazoa; Ecdysozoa; Arthropoda; Hexapoda; Insecta; Pterygota; Neoptera; Endopterygota; Diptera; Brachycera; Muscomorpha; Tephritoidea; Tephritidae; Ceratitis; Ceratitis.<br>Chromosome: Unknown, Exon count: 5 | cd08190<br>XP_004520706<br>(LOC101452868)<br>463 aa |
| *Bactrocera cucurbitae* (melon fly)<br>Eukaryota; Metazoa; Ecdysozoa; Arthropoda; Hexapoda; Insecta; Pterygota; Neoptera; Endopterygota; Diptera; Brachycera; Muscomorpha; Tephritoidea; Tephritidae; Bactrocera; Zeugodacus<br>chromosome: Unknown, Exon count: 5 | cd08190<br>XP_011177942<br>(LOC105209298)<br>467 aa |
| *Bactrocera oleae* (olive fruit fly)<br>Eukaryota; Metazoa; Ecdysozoa; Arthropoda; Hexapoda; Insecta; Pterygota; Neoptera; Endopterygota; Diptera; Brachycera; Muscomorpha; Tephritoidea; Tephritidae; Bactrocera; Daculus<br>chromosome: Unknown, Exon count: 5 | cd08190<br>XP_014103578<br>(LOC106627825)<br>467 aa |
| *Bactrocera dorsalis* (oriental fruit fly)<br>Eukaryota; Metazoa; Ecdysozoa; Arthropoda; Hexapoda; Insecta; Pterygota; Neoptera; Endopterygota; Diptera; Brachycera; Muscomorpha; Tephritoidea; Tephritidae; Bactrocera; Bactrocera<br>chromosome: Unknown, Exon count: 5 | cd08190<br>XP_011208880<br>(LOC105230013)<br>467 aa |
| *Drosophila mojavensis*<br>Eukaryota; Metazoa; Ecdysozoa; Arthropoda; Hexapoda; Insecta; Pterygota; Neoptera; Endopterygota; Diptera; Brachycera; Muscomorpha; Ephydroidea; Drosophilidae; Drosophila<br>chromosome: Unknown, Exon count: 5 | cd08190<br>XP_002005588<br>(Dmoj_GI18989)<br>466 aa |
| *Drosophila virilis*<br>Eukaryota; Metazoa; Ecdysozoa; Arthropoda; Hexapoda; Insecta; Pterygota; Neoptera; Endopterygota; Diptera; Brachycera; Muscomorpha; Ephydroidea; Drosophilidae; Drosophila<br>chromosome: Unknown, Exon count: 5 | cd08190<br>XP_002050894<br>(Dvir_GJ19950)<br>464 aa |
| *Drosophila grimshawi*<br>Eukaryota; Metazoa; Arthropoda; Hexapoda; Insecta; Pterygota; Neoptera; Endopterygota; Diptera; Brachycera; Muscomorpha; Ephydroidea; Drosophilidae; Drosophila; Hawaiian Drosophila<br>Chromosome: Unknown, Exon count: 5 | cd08190<br>XP_001987221<br>(Dgri_GH20091)<br>464 aa |
| *Drosophila willistoni*<br>Eukaryota; Metazoa; Ecdysozoa; Arthropoda; Hexapoda; Insecta; Pterygota; Neoptera; Endopterygota; Diptera; Brachycera; Muscomorpha; Ephydroidea; Drosophilidae; Drosophila; Sophophora<br>Chromosome: Unknown, Exon count: 6 | cd08190<br>XP_002061275<br>(Dwil_GK20828)<br>463 aa |
| *Drosophila busckii*<br>Eukaryota; Metazoa; Ecdysozoa; Arthropoda; Hexapoda; Insecta; Pterygota; Neoptera; Endopterygota; Diptera; Brachycera; Muscomorpha; Ephydroidea; Drosophilidae; Drosophila.<br>Chromosome: 2R, Exon count: 2 | cd08190<br>ALC40938<br>(Dbus_chr2Rg517)<br>419 aa |
| *Drosophila persimilis*<br>Eukaryota; Metazoa; Arthropoda; Hexapoda; Insecta; Pterygota; Neoptera; Endopterygota; Diptera; Brachycera; Muscomorpha; Ephydroidea; Drosophilidae; Drosophila; Sophophora<br>Chromosome: Unknown, Exon count: 5 | cd08190<br>XP_002018122<br>(Dper_GL16930)<br>464 aa |
| *Drosophila ananassae*<br>Eukaryota; Metazoa; Ecdysozoa; Arthropoda; Hexapoda; Insecta; Pterygota; Neoptera; Endopterygota; Diptera; Brachycera; Muscomorpha; Ephydroidea; Drosophilidae; Drosophila; Sophophora<br>Chromosome: Unknown, Exon count: 5 | cd08190<br>XP_001959750<br>(Dana_GF13025)<br>470 aa |
| *Drosophila melanogaster* (fruit fly)<br>Eukaryota; Metazoa; Ecdysozoa; Arthropoda; Hexapoda; Insecta; Pterygota; Neoptera; Endopterygota; Diptera; Brachycera; Muscomorpha; Ephydroidea; Drosophilidae; Drosophila; Sophophora<br>Chromosome: 2R, Exon count: 5 | cd08190<br>NP_477209<br>(Dmel_CG3425)<br>464 aa |
| *Drosophila yakuba*<br>Eukaryota; Metazoa; Ecdysozoa; Arthropoda; Hexapoda; Insecta; Pterygota; Neoptera; Endopterygota; Diptera; Brachycera; Muscomorpha; Ephydroidea; Drosophilidae; Drosophila; Sophophora<br>Chromosome: 2R, Exon count: 5 | cd08190<br>XP_002092406<br>(Dyak_GE11684)<br>464 aa |
| *Drosophila erecta*<br>Eukaryota; Metazoa; Ecdysozoa; Arthropoda; Hexapoda; Insecta; Pterygota; Neoptera; Endopterygota; Diptera; Brachycera; Muscomorpha; Ephydroidea; Drosophilidae; Drosophila; Sophophora<br>Chromosome: Unknown, Exon count: 5 | cd08190<br>XP_001975186<br>(Dere_GG20700)<br>464 aa |

| Organism[1]<br>Lineage<br>Chromosome location and exon count | Fe-ADH subfamily (conserved Domain Database)<br>Protein accesion number<br>(Gene locus)<br>Amino acid sequence lenght |
|---|---|
| *Drosophila simulans*<br>Eukaryota; Metazoa; Ecdysozoa; Arthropoda; Hexapoda; Insecta; Pterygota; Neoptera; Endopterygota; Diptera; Brachycera; Muscomorpha; Ephydroidea; Drosophilidae; Drosophila; Sophophora<br>Chromosome: 2R, Exon count:5 | cd08190<br>XP_002082603<br>(Dsimw501_GD25134)<br>464 aa |
| *Drosophila sechellia*<br>Eukaryota; Metazoa; Arthropoda; Hexapoda; Insecta; Pterygota; Neoptera; Endopterygota; Diptera; Brachycera; Muscomorpha; Ephydroidea; Drosophilidae; Drosophila; Sophophora<br>Chromosome: Unknown, Exon count: 5 | cd08190<br>XP_002039891<br>(Dsec_GM15646)<br>464 aa |
| *Diaphorina citri* (Asian citrus psyllid)<br>Eukaryota; Metazoa; Ecdysozoa; Arthropoda; Hexapoda; Insecta; Pterygota; Neoptera; Paraneoptera; Hemiptera; Sternorrhyncha; Psylliformes; Psylloidea; Psyllidae; Diaphorina<br>Chromosome: Unknown, Exon count: 10 | cd08190<br>XP_008475568<br>(LOC103512568)<br>468 aa |
| *Halyomorpha halys* (brown marmorated stink bug)<br>Eukaryota; Metazoa; Ecdysozoa; Arthropoda; Hexapoda; Insecta; Pterygota; Neoptera; Paraneoptera; Hemiptera; Euhemiptera; Heteroptera; Panheteroptera; Pentatomomorpha; Pentatomoidea; Pentatomidae; Pentatominae; Halyomorpha<br>Chromosome: Unknown, Exon count:3 | cd08190<br>XP_014273839<br>(LOC106679286)<br>471 aa |
| *Cimex lectularius* (bed bug)<br>Eukaryota; Metazoa; Ecdysozoa; Arthropoda; Hexapoda; Insecta; Pterygota; Neoptera; Paraneoptera; Hemiptera; Euhemiptera; Heteroptera; Panheteroptera; Cimicomorpha; Cimicidae; Cimex<br>Chromosome: Unknown, Exon count: 10 | cd08190<br>XP_014246816<br>(LOC106665119)<br>468 aa |
| *Limulus polyphemus* (Atlantic horseshoe crab)<br>Eukaryota; Metazoa; Ecdysozoa; Arthropoda; Chelicerata; Merostomata; Xiphosura; Limulidae; Limulus<br>chromosome: Unknown, Exon count: 14 | cd08190<br>XP_013791172<br>(LOC106475021)<br>462 aa |
| *Parasteatoda tepidariorum* (common house spider)<br>Eukaryota; Metazoa; Ecdysozoa; Arthropoda; Chelicerata; Arachnida; Araneae; Araneomorphae; Entelegynae; Araneoidea; Theridiidae; Parasteatoda<br>chromosome: Unknown, Exon count: 12 | cd08190<br>XP_015922580<br>(LOC107451106)<br>468 aa |
| *Stegodyphus mimosarum*<br>Eukaryota; Metazoa; Ecdysozoa; Arthropoda; Chelicerata; Arachnida; Araneae; Araneomorphae; Entelegynae; Eresoidea; Eresidae; Stegodyphus.<br>Chromosome: Unknown, Exon count: 14 | cd08190<br>KFM70354<br>(X975_03909)<br>464 aa |
| *Ixodes scapularis* (black-legged tick)<br>Eukaryota; Metazoa; Arthropoda; Chelicerata; Arachnida; Acari; Parasitiformes; Ixodida; Ixodoidea; Ixodidae; Ixodinae; Ixodes<br>Chromosome: Unknown, Exon count: 10 | cd08190<br>XP_002411587<br>(IscW_ISCW021748)<br>426 aa |
| Mollusca | |
| *Lottia gigantea* (owl limpet)<br>Eukaryota; Metazoa; Lophotrochozoa; Mollusca; Gastropoda; Patellogastropoda; Lottioidea; Lottiidae; Lottia<br>chromosome: Unknown, Exon count: 11 | cd08190<br>XP_009046637<br>(LOTGIDRAFT_138069)<br>419 aa |
| *Aplysia californica* (California sea hare)<br>Eukaryota; Metazoa; Lophotrochozoa; Mollusca; Gastropoda; Heterobranchia; Euthyneura; Euopisthobranchia; Aplysiomorpha; Aplysioidea; Aplysiidae; Aplysia<br>chromosome: Unknown, Exon count: 13 | cd08190<br>XP_012944844<br>(LOC101848927)<br>469 aa |
| *Biomphalaria glabrata*<br>Eukaryota; Metazoa; Lophotrochozoa; Mollusca; Gastropoda; Heterobranchia; Euthyneura; Panpulmonata; Hygrophila; Planorboidea; Planorbidae; Biomphalaria<br>chromosome: Unknown, Exon count: 14 | cd08190<br>XP_013081733<br>(LOC106067138)<br>470 aa |
| *Octopus bimaculoides*<br>Eukaryota; Metazoa; Lophotrochozoa; Mollusca; Cephalopoda; Coleoidea; Neocoleoidea; Octopodiformes; Octopoda; Incirrata; Octopodidae; Octopus<br>chromosome: Unknown, Exon count: 14 | cd08190<br>XP_014781117<br>(LOC106876888)<br>475 aa |
| *Crassostrea gigas* (Pacific oyster)<br>Eukaryota; Metazoa; Lophotrochozoa; Mollusca; Bivalvia; Pteriomorphia; Ostreoida; Ostreoidea; Ostreidae; Crassostrea<br>chromosome: Unknown, Exon count: 14 | cd08190<br>XP_011450767<br>(LOC105344644)<br>468 aa |
| Annelida | |
| *Capitella teleta*<br>Eukaryota; Metazoa; Lophotrochozoa; Annelida; Polychaeta; Scolecida; Capitellida; Capitellidae; Capitella.<br>Chromosome: Unknown, Exon count: 11 | cd08190<br>ELT90559<br>(ELT90559)<br>419 aa |
| Brachiopoda | |

| Organism[1]<br>Lineage<br>Chromosome location and exon count | Fe-ADH subfamily (conserved Domain Database)<br>Protein accesion number<br>(Gene locus)<br>Amino acid sequence lenght |
|---|---|
| *Lingula anatina*<br>Eukaryota; Metazoa; Lophotrochozoa; Brachiopoda; Linguliformea; Lingulata; Lingulida; Linguloidea; Lingulidae; Lingula.<br>Chromosome: Unknown Exon count: 14 | cd08190<br>XP_013381875<br>(LOC106152715)<br>474 aa |
| Echinodermata | |
| *Strongylocentrotus purpuratus* (purple sea urchin)<br>Eukaryota; Metazoa; Echinodermata; Eleutherozoa; Echinozoa; Echinoidea; Euechinoidea; Echinacea; Echinoida; Strongylocentrotidae; Strongylocentrotus<br>chromosome: Unknown, Exon count: 14 | cd08190<br>XP_011665376<br>(LOC575433)<br>479 aa |
| Chordata | |
| Tunicata | |
| *Oikopleura dioica*<br>Eukaryota; Metazoa; Chordata; Tunicata; Appendicularia; Oikopleuridae; Oikopleura.<br>chromosome: Unknown, Exon count: 4 | cd08190<br>CBY37636<br>CBY32066<br>(GSOID_T00031111001)<br>(GSOID_T00029363001)<br>435aa |
| *Ciona intestinalis* (vase tunicate)<br>Eukaryota; Metazoa; Chordata; Tunicata; Ascidiacea; Enterogona; Phlebobranchia; Cionidae; Ciona<br>chromosome: 1 Exon count: 12 | cd08190<br>XP_002126466<br>(LOC100186144)<br>465 aa |
| Cephalochordata | |
| *Branchiostoma floridae* (Florida lancelet)<br>Eukaryota; Metazoa; Chordata; Cephalochordata; Branchiostomidae; Branchiostoma<br>Chromosome: Unknown, Exon count: 15 | cd08190<br>XP_002603113<br>(BRAFLDRAFT_274863)<br>423 aa |
| Craniata | |
| Fishes | |
| *Callorhinchus milii* (elephant shark)<br>Eukaryota; Metazoa; Chordata; Craniata; Vertebrata; Chondrichthyes; Holocephali; Chimaeriformes; Callorhinchidae; Callorhinchus.<br>Chromosome: Unknown, Exon count: 14 | cd08190<br>XP_007894941<br>(adhfe1)<br>464 aa |
| *Scleropages formosus* (Asian bonytongue)<br>Eukaryota; Metazoa; Chordata; Craniata; Vertebrata; Euteleostomi; Actinopterygii; Neopterygii; Teleostei; Osteoglossocephala; Osteoglossomorpha; Osteoglossiformes; Osteoglossidae; Scleropages.<br>Chromosome: Unknown, Exon count: >10 | cd08190<br>KPP73706<br>(Z043_107188)<br>361 aa (fragment) |
| *Oreochromis niloticus* (Nile tilapia)<br>Eukaryota; Metazoa; Chordata; Craniata; Vertebrata; Euteleostomi; Actinopterygii; Neopterygii; Teleostei; Neoteleostei; Acanthomorphata; Ovalentaria; Cichlomorphae; Cichliformes; Cichlidae; African cichlids; Pseudocrenilabrinae; Oreochromini; Oreochromis.<br>Chromosome: Unknown, Exon count: 14 | cd08190<br>XP_003457387<br>(adhfe1)<br>466 aa |
| *Maylandia zebra* (zebra mbuna)<br>Eukaryota; Metazoa; Chordata; Craniata; Vertebrata; Euteleostomi; Actinopterygii; Neopterygii; Teleostei; Neoteleostei; Acanthomorphata; Ovalentaria; Cichlomorphae; Cichliformes; Cichlidae; African cichlids; Pseudocrenilabrinae; Haplochromini; Maylandia; Maylandia zebra complex<br>Chromosome: Unknown, Exon count: 14 | cd08190<br>XP_014263949<br>(LOC101480322)<br>466 aa |
| *Haplochromis burtoni* (Burton's mouthbrooder)<br>Eukaryota; Metazoa; Chordata; Craniata; Vertebrata; Euteleostomi; Actinopterygii; Neopterygii; Teleostei; Neoteleostei; Acanthomorphata; Ovalentaria; Cichlomorphae; Cichliformes; Cichlidae; African cichlids; Pseudocrenilabrinae; Haplochromini; Haplochromis.<br>Chromosome: Unknown, Exon count: 14 | cd08190<br>XP_005950463<br>(adhfe1)<br>466 aa |
| *Pundamilia nyererei*<br>Eukaryota; Metazoa; Chordata; Craniata; Vertebrata; Euteleostomi; Actinopterygii; Neopterygii; Teleostei; Neoteleostei; Acanthomorphata; Ovalentaria; Cichlomorphae; Cichliformes; Cichlidae; African cichlids; Pseudocrenilabrinae; Haplochromini; Pundamilia.<br>Chromosome: Unknown, Exon count: 13 | cd08190<br>XP_013770705<br>(adhfe1)<br>419 aa |
| *Neolamprologus brichardi*<br>Eukaryota; Metazoa; Chordata; Craniata; Vertebrata; Euteleostomi; Actinopterygii; Neopterygii; Teleostei; Neoteleostei; Acanthomorphata; Ovalentaria; Cichlomorphae; Cichliformes; Cichlidae; African cichlids; Pseudocrenilabrinae; Lamprologini; Neolamprologus.<br>Chromosome: Unknown, Exon count: 6 | cd08190<br>XP_006803497<br>(LOC102776229)<br>240 aa |

| Organism[1]<br>Lineage<br>Chromosome location and exon count | Fe-ADH subfamily (conserved Domain Database)<br>Protein accesion number<br>(Gene locus)<br>Amino acid sequence lenght |
|---|---|
| *Nothobranchius furzeri* (turquoise killifish)<br>Eukaryota; Metazoa; Chordata; Craniata; Vertebrata; Euteleostomi; Actinopterygii; Neopterygii; Teleostei; Neoteleostei; Acanthomorphata; Ovalentaria; Atherinomorphae; Cyprinodontiformes; Nothobranchiidae; Nothobranchius.<br>Chromosome: Unknown, Exon count: 11 | cd08190<br>XP_015796356-XP_015796357<br>(LOC107372658)<br>449 aa (corrected sequence) |
| *Stegastes partitus* (bicolor damselfish)<br>Eukaryota; Metazoa; Chordata; Craniata; Vertebrata; Euteleostomi; Actinopterygii; Neopterygii; Teleostei; Neoteleostei; Acanthomorphata; Ovalentaria; Pomacentridae; Stegastes<br>Chromosome: Unknown, Exon count: 14 | cd08190<br>XP_008304159<br>(adhfe1)<br>466 aa |
| *Austrofundulus limnaeus*<br>Eukaryota; Metazoa; Chordata; Craniata; Vertebrata; Euteleostomi; Actinopterygii; Neopterygii; Teleostei; Neoteleostei; Acanthomorphata; Ovalentaria; Atherinomorphae; Cyprinodontiformes; Rivulidae; Austrofundulus.<br>Chromosome: Unknown Exon count: >4 | cd08190<br>XP_013856312<br>(LOC106512190)<br>297 aa (fragment) |
| *Notothenia coriiceps* (black rockcod)<br>Eukaryota; Metazoa; Chordata; Craniata; Vertebrata; Euteleostomi; Actinopterygii; Neopterygii; Teleostei; Neoteleostei; Acanthomorphata; Eupercaria; Perciformes; Notothenioidei; Nototheniidae; Notothenia<br>Chromosome: Unknown, Exon count: >9 | cd08190<br>XP_010773217<br>(adhfe1)<br>367 aa (fragment) |
| *Oryzias latipes* (Japanese medaka)<br>Eukaryota; Metazoa; Chordata; Craniata; Vertebrata; Euteleostomi; Actinopterygii; Neopterygii; Teleostei; Neoteleostei; Acanthomorphata; Ovalentaria; Atherinomorphae; Beloniformes; Adrianichthyidae; Oryziinae; Oryzias.<br>Chromosome: 17, Exon count: 14 | cd08190<br>XP_004078811<br>(adhfe1)<br>466 aa |
| *Cynoglossus semilaevis* (tongue sole)<br>Eukaryota; Metazoa; Chordata; Craniata; Vertebrata; Euteleostomi; Actinopterygii; Neopterygii; Teleostei; Neoteleostei; Acanthomorphata; Carangaria; Pleuronectiformes; Pleuronectoidei; Cynoglossidae; Cynoglossinae; Cynoglossus.<br>Chromosome: Unknown, Exon count: 14 | cd08190<br>XP_008335662<br>(adhfe1)<br>466 aa |
| *Larimichthys crocea* (large yellow croaker)<br>Eukaryota; Metazoa; Chordata; Craniata; Vertebrata; Euteleostomi; Actinopterygii; Neopterygii; Teleostei; Neoteleostei; Acanthomorphata; Eupercaria; Sciaenidae; Larimichthys<br>Chromosome: Unknown, Exon count: 14 | cd08190<br>XP_010751258<br>(adhfe1)<br>464 aa |
| *Tetraodon nigroviridis* (spotted green pufferfish)<br>Eukaryota; Metazoa; Chordata; Craniata; Vertebrata; Euteleostomi; Actinopterygii; Neopterygii; Teleostei; Neoteleostei; Acanthomorphata; Eupercaria; Tetraodontiformes; Tetradontoidea; Tetraodontidae; Tetraodon<br>Chromosome: 15 Exon count: 11 | cd08190<br>CAF93117<br>(GSTEN:00008613:G:001)<br>457 aa |
| *Takifugu rubripes*<br>Eukaryota; Metazoa; Chordata; Craniata; Vertebrata; Euteleostomi; Actinopterygii; Neopterygii; Teleostei; Neoteleostei; Acanthomorphata; Eupercaria; Tetraodontiformes; Tetradontoidea; Tetraodontidae; Takifugu<br>Chromosome: Unknown, Exon count: 11 | cd08190<br>XP_003976246<br>(LOC101064882)<br>455 aa |
| *Fundulus heteroclitus* (mummichog)<br>Eukaryota; Metazoa; Chordata; Craniata; Vertebrata; Euteleostomi; Actinopterygii; Neopterygii; Teleostei; Neoteleostei; Acanthomorphata; Ovalentaria; Atherinomorphae; Cyprinodontiformes; Fundulidae; Fundulus.<br>Chromosome: Unknown, Exon count: 3 | cd08190<br>XP_012715137<br>(LOC105923734)<br>419 aa |
| *Cyprinodon variegatus* (sheepshead minnow)<br>Eukaryota; Metazoa; Chordata; Craniata; Vertebrata; Euteleostomi; Actinopterygii; Neopterygii; Teleostei; Neoteleostei; Acanthomorphata; Ovalentaria; Atherinomorphae; Cyprinodontiformes; Cyprinodontidae; Cyprinodon<br>Chromosome. Unknown, Exon count: 13 | cd08190<br>XP_015242477<br>(adhfe1)<br>419 aa |
| *Poecilia mexicana*<br>Eukaryota; Metazoa; Chordata; Craniata; Vertebrata; Euteleostomi; Actinopterygii; Neopterygii; Teleostei; Neoteleostei; Acanthomorphata; Ovalentaria; Atherinomorphae; Cyprinodontiformes; Poeciliidae; Poeciliinae; Poecilia.<br>Chromosome: Unknown, Exon count: 14 | cd08190<br>XP_014828835<br>(adhfe1)<br>464 aa |
| *Poecilia formosa* (Amazon molly)<br>Eukaryota; Metazoa; Chordata; Craniata; Vertebrata; Euteleostomi; Actinopterygii; Neopterygii; Teleostei; Neoteleostei; Acanthomorphata; Ovalentaria; Atherinomorphae; Cyprinodontiformes; Poeciliidae; Poeciliinae; Poecilia<br>Chromosome: Unknown, Exon count: 14 | cd08190<br>XP_007541233<br>(adhfe1)<br>464 aa |
| *Xiphophorus maculatus* (southern platyfish)<br>Eukaryota; Metazoa; Chordata; Craniata; Vertebrata; Euteleostomi; Actinopterygii; Neopterygii; Teleostei; Neoteleostei; Acanthomorphata; Ovalentaria; Atherinomorphae; Cyprinodontiformes; Poeciliidae; Poeciliinae; Xiphophorus.<br>Chromosome: Unknown, Exon count: 13 | cd08190<br>XP_014331166<br>(adhfe1)<br>434 aa |

| Organism[1]<br>Lineage<br>Chromosome location and exon count | Fe-ADH subfamily (conserved Domain Database)<br>Protein accesion number<br>(Gene locus)<br>Amino acid sequence lenght |
|---|---|
| *Poecilia reticulata* (guppy)<br>Eukaryota; Metazoa; Chordata; Craniata; Vertebrata; Euteleostomi; Actinopterygii; Neopterygii; Teleostei; Neoteleostei; Acanthomorphata; Ovalentaria; Atherinomorphae; Cyprinodontiformes; Poeciliidae; Poeciliinae; Poecilia<br>Chromosome: Unknown, Exon count: 14 | cd08190<br>XP_008400883<br>(adhfe1)<br>456 aa |
| *Poecilia latipinna* (sailfin molly)<br>Eukaryota; Metazoa; Chordata; Craniata; Vertebrata; Euteleostomi; Actinopterygii; Neopterygii; Teleostei; Neoteleostei; Acanthomorphata; Ovalentaria; Atherinomorphae; Cyprinodontiformes; Poeciliidae; Poeciliinae; Poecilia<br>Chromosome: Unknown, Exon count: 13 | cd08190<br>XP_014881949<br>(adhfe1)<br>452 aa |
| *Salmo salar* (Atlantic salmon)<br>Eukaryota; Metazoa; Chordata; Craniata; Vertebrata; Euteleostomi; Actinopterygii; Neopterygii; Teleostei; Protacanthopterygii; Salmoniformes; Salmonidae; Salmoninae; Salmo<br>Chromosome: ssa14, Exon count: 15 | cd08190<br>XP_013995459<br>(adhfe1)<br>464 aa |
| *Oncorhynchus mykiss* (rainbow trout)<br>Eukaryota; Metazoa; Chordata; Craniata; Vertebrata; Euteleostomi; Actinopterygii; Neopterygii; Teleostei; Protacanthopterygii; Salmoniformes; Salmonidae; Salmoninae; Oncorhynchus.<br>Chromosome: Unknown, Exon count: 15 | cd08190<br>CDQ73949<br>(GSONMT00049544001)<br>464 aa |
| *Esox lucius* (northern pike)<br>Eukaryota; Metazoa; Chordata; Craniata; Vertebrata; Euteleostomi; Actinopterygii; Neopterygii; Teleostei; Protacanthopterygii; Esociformes; Esocidae; Esox<br>Chromosome: LG03, Exon count: 14 | cd08190<br>XP_012994717<br>(adhfe1)<br>464 aa |
| *Lepisosteus oculatus* (spotted gar)<br>Eukaryota; Metazoa; Chordata; Craniata; Vertebrata; Euteleostomi; Actinopterygii; Neopterygii; Holostei; Semionotiformes; Lepisosteidae; Lepisosteus.<br>Chromosome: LG9, Exon count: 14 | cd08190<br>XP_006633963<br>(adhfe1)<br>465 aa |
| *Danio rerio* (zebrafish)<br>Eukaryota; Metazoa; Chordata; Craniata; Vertebrata; Euteleostomi; Actinopterygii; Neopterygii; Teleostei; Ostariophysi; Cypriniformes; Cyprinidae; Danio<br>Chromosome: 2, Exon count: 15 | cd08190<br>NP_996969<br>(adhfe1)<br>509 aa |
| *Cyprinus carpio* (common carp)<br>Eukaryota; Metazoa; Chordata; Craniata; Vertebrata; Euteleostomi; Actinopterygii; Neopterygii; Teleostei; Ostariophysi; Cypriniformes; Cyprinidae; Cyprinus.<br>Chromosome: Unknown, Exon count: 14 | cd08190<br>KTF94921<br>(cypCar_00005923)<br>519 aa |
| *Astyanax mexicanus* (Mexican tetra)<br>Eukaryota; Metazoa; Chordata; Craniata; Vertebrata; Euteleostomi; Actinopterygii; Neopterygii; Teleostei; Ostariophysi; Characiformes; Characoidei; Characidae; Characidae incertae sedis; Astyanax clade; Astyanax<br>Chromosome: Unknown, Exon count: 14 | cd08190<br>XP_007237697<br>(adhfe1)<br>469aa |
| Amphibia | |
| *Xenopus tropicalis* (tropical clawed frog)<br>Eukaryota; Metazoa; Chordata; Craniata; Vertebrata; Euteleostomi; Amphibia; Batrachia; Anura; Pipoidea; Pipidae; Xenopodinae; Xenopus; Silurana<br>Chromosome: Unknown, Exon count: 15 | cd08190<br>NP_989277<br>(adhfe1)<br>463 aa |
| *Xenopus laevis* (African clawed frog)<br>Eukaryota; Metazoa; Chordata; Craniata; Vertebrata; Euteleostomi; Amphibia; Batrachia; Anura; Pipoidea; Pipidae; Xenopodinae; Xenopus; Xenopus<br>Chromosome: Unknown, Exon count:11 | cd08190<br>NP_001121274<br>(adhfe1)<br>463 aa |
| Reptilia | |
| *Pelodiscus sinensis* (Chinese soft-shelled turtle)<br>Eukaryota; Metazoa; Chordata; Craniata; Vertebrata; Euteleostomi; Archelosauria; Testudines; Cryptodira; Trionychia; Trionychidae; Pelodiscus<br>Chromosome: Unknown, Exon count: 15 | cd08190<br>XP_014433250<br>(ADHFE1)<br>493 aa |
| *Chelonia mydas* (green sea turtle)<br>Eukaryota; Metazoa; Chordata; Craniata; Vertebrata; Euteleostomi; Archelosauria; Testudines; Cryptodira; Durocryptodira; Americhelydia; Chelonioidea; Cheloniidae; Chelonia<br>Chromosome: Unknown, Exon count: >9 | cd08190<br>EMP29794<br>(UY3_13077)<br>436 aa (fragment) |
| *Chrysemys picta bellii* (western painted turtle)<br>Eukaryota; Metazoa; Chordata; Craniata; Vertebrata; Euteleostomi; Archelosauria; Testudines; Cryptodira; Durocryptodira; Testudinoidea; Emydidae; Chrysemys<br>Chromosome: 2, Exon count: 14 | cd08190<br>XP_005288141<br>(ADHFE1)<br>465 aa |
| *Python bivittatus* (Burmese python)<br>Eukaryota; Metazoa; Chordata; Craniata; Vertebrata; Euteleostomi; Lepidosauria; Squamata; Bifurcata; Unidentata; Episquamata; Toxicofera; Serpentes; Henophidia; Pythonidae; Python<br>Chromosome: Unknown, Exon count: 14 | cd08190<br>XP_007426778<br>(ADHFE1)<br>466 aa |

| Organism[1]<br>Lineage<br>Chromosome location and exon count | Fe-ADH subfamily (conserved Domain Database)<br>Protein accesion number<br>(Gene locus)<br>Amino acid sequence lenght |
|---|---|
| *Thamnophis sirtalis*<br>Eukaryota; Metazoa; Chordata; Craniata; Vertebrata; Euteleostomi; Lepidosauria; Squamata; Bifurcata; Unidentata; Episquamata; Toxicofera; Serpentes; Colubroidea; Colubridae; Natricinae; Thamnophis<br>Chromosome: Unknown, Exon count: 7 | cd08190<br>XP_013916559<br>(LOC106544726)<br>419 aa |
| *Ophiophagus hannah* (king cobra)<br>Eukaryota; Metazoa; Chordata; Craniata; Vertebrata; Euteleostomi; Lepidosauria; Squamata; Bifurcata; Unidentata; Episquamata; Toxicofera; Serpentes; Colubroidea; Elapidae; Elapinae; Ophiophagus.<br>Chromosome: Unknown, Exon count 12: | cd08190<br>ETE70508<br>(Adhfe1)<br>(L345_03677)<br>443 aa |
| *Protobothrops mucrosquamatus*<br>Eukaryota; Metazoa; Chordata; Craniata; Vertebrata; Euteleostomi; Lepidosauria; Squamata; Bifurcata; Unidentata; Episquamata; Toxicofera; Serpentes; Colubroidea; Viperidae; Crotalinae; Protobothrops<br>Chromosome: Unknown, Exon count: 14 | cd08190<br>XP_015679893<br>(ADHFE1)<br>466 aa |
| *Anolis carolinensis* (green anole)<br>Eukaryota; Metazoa; Chordata; Craniata; Vertebrata; Euteleostomi; Lepidosauria; Squamata; Bifurcata; Unidentata; Episquamata; Toxicofera; Iguania; Iguanidae; Polychrotinae; Anolis<br>Chromosome: 4, Exon count: 14 | cd08190<br>XP_003219674<br>(adhfe1)<br>465 aa |
| *Gekko japonicus*<br>Eukaryota; Metazoa; Chordata; Craniata; Vertebrata; Euteleostomi; Lepidosauria; Squamata; Bifurcata; Gekkota; Gekkonidae; Gekkoninae; Gekko<br>Chromosome: Unknown, Exon count: 14 | cd08190<br>XP_015264450<br>(ADHFE1)<br>465 aa |
| *Alligator mississippiensis* (American alligator)<br>Eukaryota; Metazoa; Chordata; Craniata; Vertebrata; Euteleostomi; Archelosauria; Archosauria; Crocodylia; Alligatoridae; Alligatorinae; Alligator.<br>Chromosome: Unknown, Exon count: 15 | cd08190<br>XP_006262059<br>(ADHFE1)<br>465aa |
| *Alligator sinensis* (Chinese alligator)<br>Eukaryota; Metazoa; Chordata; Craniata; Vertebrata; Euteleostomi; Archelosauria; Archosauria; Crocodylia; Alligatoridae; Alligatorinae; Alligator.<br>Chromosome: Unknown, Exon count: 15 | cd08190<br>XP_006038200<br>(ADHFE1)<br>497aa |
| Aves | |
| *Tinamus guttatus* (white-throated tinamou)<br>Eukaryota; Metazoa; Chordata; Craniata; Vertebrata; Euteleostomi; Archelosauria; Archosauria; Dinosauria; Saurischia; Theropoda;Coelurosauria; Aves; Palaeognathae; Tinamiformes; Tinamidae; Tinamus.<br>Chromosome: Unknown, Exon count: 13 | cd08190<br>XP_010214354<br>(ADHFE1)<br>419aa |
| *Picoides pubescens* (downy woodpecker)<br>Eukaryota; Metazoa; Chordata; Craniata; Vertebrata; Euteleostomi; Archelosauria; Archosauria; Dinosauria; Saurischia; Theropoda; Coelurosauria; Aves; Neognathae; Piciformes; Picidae; Picoides.<br>Chromosome: Unknown, Exon count: 14 | cd08190<br>XP_009902394<br>(ADHFE1)<br>419 aa |
| *Colius striatus* (speckled mousebird)<br>Eukaryota; Metazoa; Chordata; Craniata; Vertebrata; Euteleostomi; Archelosauria; Archosauria; Dinosauria; Saurischia; Theropoda; Coelurosauria; Aves; Neognathae; Coliiformes; Coliidae; Colius.<br>Chromosome: Unknown, Exon count: 7 | cd08190<br>KFP28675<br>(ADHFE1)<br>257 aa |
| *Gallus gallus* (chicken)<br>Eukaryota; Metazoa; Chordata; Craniata; Vertebrata; Euteleostomi; Archelosauria; Archosauria; Dinosauria; Saurischia; Theropoda; Coelurosauria; Aves; Neognathae; Galloanserae; Galliformes; Phasianidae; Phasianinae; Gallus.<br>Chromosome: 2, Exon count: 15 | cd08190<br>XP_015138234<br>(ADHFE1)<br>465 aa |
| *Coturnix japonica* (Japanese quail)<br>Eukaryota; Metazoa; Chordata; Craniata; Vertebrata; Euteleostomi; Archelosauria; Archosauria; Dinosauria; Saurischia; Theropoda; Coelurosauria; Aves; Neognathae; Galloanserae; Galliformes; Phasianidae; Perdicinae; Coturnix.<br>Chromosome: 2, Exon count: 14 | cd08190<br>XP_015711224<br>(ADHFE1)<br>465 aa |
| *Aquila chrysaetos canadensis*<br>Eukaryota; Metazoa; Chordata; Craniata; Vertebrata; Euteleostomi; Archelosauria; Archosauria; Dinosauria; Saurischia; Theropoda; Coelurosauria; Aves; Neognathae; Falconiformes; Accipitridae; Accipitrinae; Aquila.<br>Chromosome: Unknown, Exon count: 18 | cd08190<br>XP_011582102<br>(ADHFE1)<br>465 aa |
| *Melopsittacus undulatus* (budgerigar)<br>Eukaryota; Metazoa; Chordata; Craniata; Vertebrata; Euteleostomi; Archelosauria; Archosauria; Dinosauria; Saurischia; Theropoda; Coelurosauria; Aves; Neognathae; Psittaciformes; Psittaculidae; Melopsittacus<br>Chromosome: Unknown, Exon count: 14 | cd08190<br>XP_005150839<br>(LOC101871861)<br>465 aa |

| Organism[1]<br>Lineage<br>Chromosome location and exon count | Fe-ADH subfamily (conserved Domain Database)<br>Protein accesion number<br>(Gene locus)<br>Amino acid sequence lenght |
|---|---|
| *Phoenicopterus ruber ruber*<br>Eukaryota; Metazoa; Chordata; Craniata; Vertebrata; Euteleostomi; Archelosauria; Archosauria; Dinosauria; Saurischia; Theropoda; Coelurosauria; Aves; Neognathae; Phoenicopteriformes; Phoenicopteridae; Phoenicopterus.<br>Chromosome: Unknown, Exon count: 11 | cd08190<br>KFQ87897<br>(N337_07503)<br>419 aa |
| *Calidris pugnax* (ruff)<br>Eukaryota; Metazoa; Chordata; Craniata; Vertebrata; Euteleostomi; Archelosauria; Archosauria; Dinosauria; Saurischia; Theropoda; Coelurosauria; Aves; Neognathae; Charadriiformes; Scolopacidae; Calidris.<br>Chromosome: Unknown, Exon count: 15 | cd08190<br>XP_014795156<br>(ADHFE1)<br>465 aa |
| *Ficedula albicollis* (collared flycatcher)<br>Eukaryota; Metazoa; Chordata; Craniata; Vertebrata; Euteleostomi;Archelosauria; Archosauria; Dinosauria; Saurischia; Theropoda; Coelurosauria; Aves; Neognathae; Passeriformes; Muscicapidae; Ficedula.<br>Chromosome: 2, Exon count: 14 | cd08190<br>XP_005042222<br>(ADHFE1)<br>497 aa |
| *Acanthisitta chloris* (rifleman)<br>Eukaryota; Metazoa; Chordata; Craniata; Vertebrata; Euteleostomi; Archelosauria; Archosauria; Dinosauria; Saurischia; Theropoda; Coelurosauria; Aves; Neognathae; Passeriformes; Acanthisittidae; Acanthisitta.<br>Chromosome: Unknown, Exon count: 11 | cd08190<br>XP_009073741<br>(ADHFE1)<br>446 aa |
| *Corvus cornix cornix*<br>Eukaryota; Metazoa; Chordata; Craniata; Vertebrata; Euteleostomi; Archelosauria; Archosauria; Dinosauria; Saurischia; Theropoda; Coelurosauria; Aves; Neognathae; Passeriformes; Corvoidea; Corvidae; Corvus.<br>Chromosome: Unknown, Exon count: 13 | cd08190<br>XP_010403438<br>(ADHFE1)<br>419 aa |
| *Corvus brachyrhynchos* (American crow)<br>Eukaryota; Metazoa; Chordata; Craniata; Vertebrata; Euteleostomi; Archelosauria; Archosauria; Dinosauria; Saurischia; Theropoda; Coelurosauria; Aves; Neognathae; Passeriformes; Corvoidea; Corvidae; Corvus.<br>Chromosome: Unknown, Exon count: 14 | cd08190<br>XP_008630332<br>(ADHFE1)<br>465 aa |
| *Parus major* (Great Tit)<br>Eukaryota; Metazoa; Chordata; Craniata; Vertebrata; Euteleostomi; Archelosauria; Archosauria; Dinosauria; Saurischia; Theropoda; Coelurosauria; Aves; Neognathae; Passeriformes; Paridae; Parus.<br>Chromosome: 2, Exon count: 15 | cd08190<br>XP_015474299<br>(ADHFE1)<br>465 aa |
| *Pseudopodoces humilis* (Tibetan ground-tit)<br>Eukaryota; Metazoa; Chordata; Craniata; Vertebrata; Euteleostomi; Archelosauria; Archosauria; Dinosauria; Saurischia; Theropoda; Coelurosauria; Aves; Neognathae; Passeriformes; Paridae; Pseudopodoces.<br>Chromosome: Unknown, Exon count:15 | cd08190<br>XP_005517174<br>(ADHFE1)<br>465 aa |
| *Sturnus vulgaris* (common starling)<br>Eukaryota; Metazoa; Chordata; Craniata; Vertebrata; Euteleostomi; Archelosauria; Archosauria; Dinosauria; Saurischia; Theropoda; Coelurosauria; Aves; Neognathae; Passeriformes; Sturnidae; Sturnus.<br>Chromosome: Unknown, Exon count: 14 | cd08190<br>XP_014728512<br>(ADHFE1)<br>465aa |
| *Taeniopygia guttata* (zebra finch)<br>Eukaryota; Metazoa; Chordata; Craniata; Vertebrata; Euteleostomi; Archelosauria; Archosauria; Dinosauria; Saurischia; Theropoda; Coelurosauria; Aves; Neognathae; Passeriformes; Passeroidea; Estrildidae; Estrildinae; Taeniopygia.<br>Chromosome: 2, Exon count: 11 | cd08190<br>XP_002198853<br>(ADHFE1)<br>465aa |
| *Geospiza fortis* (medium ground-finch)<br>Eukaryota; Metazoa; Chordata; Craniata; Vertebrata; Euteleostomi;Archelosauria; Archosauria; Dinosauria; Saurischia; Theropoda Coelurosauria; Aves; Neognathae;Passeriformes; Thraupidae;Geospiza.<br>Chromosome: Unknown, Exon count: 14 | cd08190<br>XP_014163326<br>(ADHFE1)<br>449aa |
| *Serinus canaria* (common canary)<br>Eukaryota; Metazoa; Chordata; Craniata; Vertebrata; Euteleostomi; Archelosauria; Archosauria; Dinosauria; Saurischia; Theropoda; Coelurosauria; Aves; Neognathae; Passeriformes; Passeroidea; Fringillidae; Carduelinae; Serinus.<br>Chromosome: Unknown, Exon count: 14 | cd08190<br>XP_009083936<br>(ADHFE1)<br>465aa |
| *Zonotrichia albicollis* (white-throated sparrow)<br>Eukaryota; Metazoa; Chordata; Craniata; Vertebrata; Euteleostomi; Archelosauria; Archosauria; Dinosauria; Saurischia; Theropoda; Coelurosauria; Aves; Neognathae; Passeriformes; Passerellidae; Zonotrichia.<br>Chromosome: Unknown, Exon count: 14 | cd08190<br>XP_005479403<br>(ADHFE1)<br>465aa |
| *Apaloderma vittatum* (bar-tailed trogon<br>Eukaryota; Metazoa; Chordata; Craniata; Vertebrata; Euteleostomi;Archelosauria; Archosauria; Dinosauria; Saurischia; Theropoda; Coelurosauria; Aves; Neognathae; Trogoniformes; Trogonidae; Apaloderma.<br>Chromosome: Unknown, Exon count: 13 | cd08190<br>XP_009863349<br>(ADHFE1)<br>418aa |

| Organism[1]<br>Lineage<br>Chromosome location and exon count | Fe-ADH subfamily (conserved Domain Database)<br>Protein accesion number<br>(Gene locus)<br>Amino acid sequence lenght |
|---|---|
| *Amazona aestiva* (blue-fronted amazon)<br>Eukaryota; Metazoa; Chordata; Craniata; Vertebrata; Euteleostomi; Archelosauria; Archosauria; Dinosauria; Saurischia; Theropoda; Coelurosauria; Aves; Neognathae; Psittaciformes; Psittacidae; Amazona<br>Chromosome: Unknown, Exon count: 11 | cd08190<br>KQK76336<br>(ADHFE1)<br>(AAES_136432)<br>419aa |
| *Balearica regulorum gibbericeps* (East African grey crowned-crane)<br>Eukaryota; Metazoa; Chordata; Craniata; Vertebrata; Euteleostomi; Archelosauria; Archosauria; Dinosauria; Saurischia; Theropoda; Coelurosauria; Aves; Neognathae; Gruiformes; Gruidae; Balearica.<br>Chromosome: Unknown, Exon count: >6 | cd08190<br>XP_010303426<br>(ADHFE1)<br>238aa (fragment) |
| *Anser cygnoides domesticus*<br>Eukaryota; Metazoa; Chordata; Craniata; Vertebrata; Euteleostomi; Archelosauria; Archosauria; Dinosauria; Saurischia; Theropoda; Coelurosauria; Aves; Neognathae; Galloanserae; Anseriformes; Anatidae; Anser<br>Chromosome: Unknown, Exon count: 14 | cd08190<br>XP_013028001<br>(ADHFE1)<br>471aa |
| *Gavia stellata* (red-throated loon)<br>Eukaryota; Metazoa; Chordata; Craniata; Vertebrata; Euteleostomi; Archelosauria; Archosauria; Dinosauria; Saurischia; Theropoda; Coelurosauria; Aves; Neognathae; Gaviiformes; Gaviidae; Gavia.<br>Chromosome: Unknown, Exon count: 13 | C8190D0<br>KFV52854<br>(ADHFE1)<br>422aa |
| *Egretta garzetta* (little egret)<br>Eukaryota; Metazoa; Chordata; Craniata; Vertebrata; Euteleostomi; Archelosauria; Archosauria; Dinosauria; Saurischia; Theropoda; Coelurosauria; Aves; Neognathae; Pelecaniformes; Ardeidae; Egretta<br>Chromosome: Unknown, Exon count: 13 | cd08190<br>XP_009632402<br>(ADHFE1)<br>421aa |
| *Opisthocomus hoazin*<br>Eukaryota; Metazoa; Chordata; Craniata; Vertebrata; Euteleostomi; Archelosauria; Archosauria; Dinosauria; Saurischia; Theropoda; Coelurosauria; Aves; Neognathae; Opisthocomiformes; Opisthocomidae; Opisthocomus.<br>Chromosome: Unknown, Exon count: 11 | cd08190<br>XP_009938795<br>(ADHFE1)<br>414aa |
| *Anas platyrhynchos* (mallard)<br>Eukaryota; Metazoa; Chordata; Craniata; Vertebrata; Euteleostomi; Archelosauria; Archosauria; Dinosauria; Saurischia; Theropoda; Coelurosauria; Aves; Neognathae; Galloanserae; Anseriformes; Anatidae; Anas.<br>Chromosome: Unknown, Exon count: 17 | cd08190<br>XP_012954771<br>(ADHFE1)<br>459aa |
| *Haliaeetus leucocephalus* (bald eagle)<br>Eukaryota; Metazoa; Chordata; Craniata; Vertebrata; Euteleostomi; Archelosauria; Archosauria; Dinosauria; Saurischia; Theropoda; Coelurosauria; Aves; Neognathae; Falconiformes; Accipitridae; Accipitrinae; Haliaeetus.<br>Chromosome: Unknown, Exon count: 13 | cd08190<br>XP_010574215<br>(ADHFE1)<br>419aa |
| *Haliaeetus albicilla* (white-tailed eagle)<br>Eukaryota; Metazoa; Chordata; Craniata; Vertebrata; Euteleostomi; Archelosauria; Archosauria; Dinosauria; Saurischia; Theropoda; Coelurosauria; Aves; Neognathae; Falconiformes; Accipitridae; Accipitrinae; Haliaeetus<br>Chromosome: Unknown, Exon count: 10 | cd08190<br>KFQ09702<br>(ADHFE1)<br>401aa |
| *Phaethon lepturus* (white-tailed tropicbird)<br>Eukaryota; Metazoa; Chordata; Craniata; Vertebrata; Euteleostomi; Archelosauria; Archosauria; Dinosauria; Saurischia; Theropoda; Coelurosauria; Aves; Neognathae; Pelecaniformes; Phaethontidae; Phaethon.<br>Chromosome: Unknown, Exon count: 13 | cd08190<br>XP_010285164<br>(ADHFE1)<br>423aa |
| *Fulmarus glacialis* (northern fulmar)<br>Eukaryota; Metazoa; Chordata; Craniata; Vertebrata; Euteleostomi; Archelosauria; Archosauria; Dinosauria; Saurischia; Theropoda; Coelurosauria; Aves; Neognathae; Procellariiformes; Procellariidae; Procellariinae; Fulmarus.<br>Chromosome: Unknown, Exon count: 13 | cd08190<br>XP_009579219<br>(ADHFE1)<br>419aa |
| *Eurypyga helias* (sunbittern)<br>Eukaryota; Metazoa; Chordata; Craniata; Vertebrata; Euteleostomi; Archelosauria; Archosauria; Dinosauria; Saurischia; Theropoda; Coelurosauria; Aves; Neognathae; Gruiformes; Eurypygidae; Eurypyga.<br>Chromosome: Unknown, Exon count: 13 | cd08190<br>XP_010145632<br>(ADHFE1)<br>417aa |
| *Tyto alba* (barn owl)<br>Eukaryota; Metazoa; Chordata; Craniata; Vertebrata; Euteleostomi; Archelosauria; Archosauria; Dinosauria; Saurischia; Theropoda; Coelurosauria; Aves; Neognathae; Strigiformes; Tytonidae; Tyto.<br>Chromosome: Unknown, Exon count: 12 | cd08190<br>XP_009965404<br>(ADHFE1)<br>419aa |
| *Charadrius vociferus* (killdeer)<br>Eukaryota; Metazoa; Chordata; Craniata; Vertebrata; Euteleostomi; Archelosauria; Archosauria; Dinosauria; Saurischia; Theropoda; Coelurosauria; Aves; Neognathae; Charadriiformes; Charadriidae; Charadrius.<br>Chromosome: Unknown, Exon count: 12 | cd08190<br>XP_009883874<br>(ADHFE1)<br>419aa |

| Organism[1]<br>Lineage<br>Chromosome location and exon count | Fe-ADH subfamily (conserved Domain Database)<br>Protein accesion number<br>(Gene locus)<br>Amino acid sequence lenght |
|---|---|
| *Pterocles gutturalis* (yellow-throated sandgrouse)<br>Eukaryota; Metazoa; Chordata; Craniata; Vertebrata; Euteleostomi; Archelosauria; Archosauria; Dinosauria; Saurischia; Theropoda; Coelurosauria; Aves; Neognathae; Ciconiiformes; Pteroclidae; Pterocles.<br>Chromosome: Unknown, Exon count: 13 | cd08190<br>XP_010084715<br>(ADHFE1)<br>419aa |
| *Podiceps cristatus* (great crested grebe)<br>Eukaryota; Metazoa; Chordata; Craniata; Vertebrata; Euteleostomi; Archelosauria; Archosauria; Dinosauria; Saurischia; Theropoda; Coelurosauria; Aves; Neognathae; Podicipediformes; Podicipedidae; Podiceps.<br>Chromosome: Unknown, Exon count: 11 | cd08190<br>KFZ56980<br>(ADHFE1)<br>(N338_04861)<br>419aa |
| *Phalacrocorax carbo* (great cormorant)<br>Eukaryota; Metazoa; Chordata; Craniata; Vertebrata; Euteleostomi; Archelosauria; Archosauria; Dinosauria; Saurischia; Theropoda; Coelurosauria; Aves; Neognathae; Pelecaniformes; Phalacrocoracidae; Phalacrocorax.<br>Chromosome: Unknown, Exon count: 14 | cd08190<br>XP_009513406<br>(ADHFE1)<br>419aa |
| *Pelecanus crispus* (Dalmatian pelican)<br>Eukaryota; Metazoa; Chordata; Craniata; Vertebrata; Euteleostomi; Archelosauria; Archosauria; Dinosauria; Saurischia; Theropoda; Coelurosauria; Aves; Neognathae; Pelecaniformes; Pelecanidae; Pelecanus.<br>Chromosome: Unknown, Exon count: 13 | cd08190<br>XP_009484909<br>(ADHFE1)<br>419aa |
| *Merops nubicus* (carmine bee-eater)<br>Eukaryota; Metazoa; Chordata; Craniata; Vertebrata; Euteleostomi; Archelosauria; Archosauria; Dinosauria; Saurischia; Theropoda; Coelurosauria; Aves; Neognathae; Coraciiformes; Meropidae; Merops<br>Chromosome: Unknown, Exon count: 13 | cd08190<br>XP_008943103<br>(ADHFE1)<br>446aa |
| *Falco cherrug* (Saker falcon)<br>Eukaryota; Metazoa; Chordata; Craniata; Vertebrata; Euteleostomi; Archelosauria; Archosauria; Dinosauria; Saurischia; Theropoda; Coelurosauria; Aves; Neognathae; Falconiformes; Falconidae; Falco<br>Chromosome: Unknown, Exon count: 13 | cd08190<br>XP_014138674<br>(ADHFE1)<br>511aa |
| *Falco peregrinus* (peregrine falcon)<br>Eukaryota; Metazoa; Chordata; Craniata; Vertebrata; Euteleostomi; Archelosauria; Archosauria; Dinosauria; Saurischia; Theropoda; Coelurosauria; Aves; Neognathae; Falconiformes; Falconidae; Falco.<br>Chromosome: Unknown, Exon count: 16 | cd08190<br>XP_013157465<br>(ADHFE1)<br>577aa |
| *Cariama cristata* (red-legged seriema)<br>Eukaryota; Metazoa; Chordata; Craniata; Vertebrata; Euteleostomi; Archelosauria; Archosauria; Dinosauria; Saurischia; Theropoda; Coelurosauria; Aves; Neognathae; Gruiformes; Cariamidae; Cariama.<br>Chromosome: Unknown, Exon count: 13 | cd08190<br>XP_009708659<br>(ADHFE1)<br>419aa |
| *Struthio camelus australis*<br>Eukaryota; Metazoa; Chordata; Craniata; Vertebrata; Euteleostomi; Archelosauria; Archosauria; Dinosauria; Saurischia; Theropoda; Coelurosauria; Aves; Palaeognathae; Struthioniformes; Struthionidae; Struthio.<br>Chromosome: Unknown, Exon count: 14 | cd08190<br>XP_009666702<br>(ADHFE1)<br>419aa |
| *Apteryx australis mantelli*<br>Eukaryota; Metazoa; Chordata; Craniata; Vertebrata; Euteleostomi; Archelosauria; Archosauria; Dinosauria; Saurischia; Theropoda; Coelurosauria; Aves; Palaeognathae; Apterygiformes; Apterygidae; Apteryx.<br>Chromosome: Unknown, Exon count: 12 | cd08190<br>XP_013798266<br>(ADHFE1)<br>419aa |
| *Tauraco erythrolophus* (red-crested turaco)<br>Eukaryota; Metazoa; Chordata; Craniata; Vertebrata; Euteleostomi; Archelosauria; Archosauria; Dinosauria; Saurischia; Theropoda; Coelurosauria; Aves; Neognathae; Musophagiformes; Musophagidae; Tauraco<br>Chromosome: Unknown, Exon count: 13 | cd08190<br>XP_009979269<br>(ADHFE1)<br>419aa |
| *Cuculus canorus* (common cuckoo)<br>Eukaryota; Metazoa; Chordata; Craniata; Vertebrata; Euteleostomi; Archelosauria; Archosauria; Dinosauria; Saurischia; Theropoda; Coelurosauria; Aves; Neognathae; Cuculiformes; Cuculidae; Cuculus.<br>Chromosome: Unknown, Exon count: 13 | cd08190<br>XP_009569179<br>(ADHFE1)<br>420aa |
| *Manacus vitellinus* (golden-collared manakin)<br>Eukaryota; Metazoa; Chordata; Craniata; Vertebrata; Euteleostomi; Archelosauria; Archosauria; Dinosauria; Saurischia; Theropoda; Coelurosauria; Aves; Neognathae; Passeriformes; Pipridae; Manacus.<br>Chromosome: Unknown, Exon count: 15 | cd08190<br>KFW88035<br>(ADHFE1)<br>419aa |
| *Nipponia nippon* (crested ibis)<br>Eukaryota; Metazoa; Chordata; Craniata; Vertebrata; Euteleostomi; Archelosauria; Archosauria; Dinosauria; Saurischia; Theropoda; Coelurosauria; Aves; Neognathae; Pelecaniformes; Threskiornithidae;Nipponia.<br>Chromosome: Unknown, Exon count: 13 | cd08190<br>XP_009469923<br>(ADHFE1)<br>414aa |

| Organism[1]<br>Lineage<br>Chromosome location and exon count | Fe-ADH subfamily (conserved Domain Database)<br>Protein accesion number<br>(Gene locus)<br>Amino acid sequence lenght |
|---|---|
| *Chlamydotis macqueenii* (Macqueen's bustard)<br>Eukaryota; Metazoa; Chordata; Craniata; Vertebrata; Euteleostomi; Archelosauria; Archosauria; Dinosauria; Saurischia; Theropoda; Coelurosauria; Aves; Neognathae; Gruiformes; Otididae; Chlamydotis.<br>Chromosome: Unknown, Exon count: 13 | cd08190<br>XP_010120823<br>(ADHFE1)<br>419aa |
| *Chaetura pelagica* (chimney swift)<br>Eukaryota; Metazoa; Chordata; Craniata; Vertebrata; Euteleostomi; Archelosauria; Archosauria; Dinosauria; Saurischia; Theropoda; Coelurosauria; Aves; Neognathae; Apodiformes; Apodidae; Chaetura.<br>Chromosome: Unknown, Exon count: 13 | cd08190<br>XP_009993856<br>(ADHFE1)<br>419aa |
| *Leptosomus discolor* (cuckoo roller)<br>Eukaryota; Metazoa; Chordata; Craniata; Vertebrata; Euteleostomi; Archelosauria; Archosauria; Dinosauria; Saurischia; Theropoda; Coelurosauria; Aves; Neognathae; Coraciiformes; Leptosomidae; Leptosomus.<br>Chromosome: Unknown, Exon count: 13 | cd08190<br>XP_009950900<br>(ADHFE1)<br>419aa |
| *Mesitornis unicolor* (brown roatelo)<br>Eukaryota; Metazoa; Chordata; Craniata; Vertebrata; Euteleostomi; Archelosauria; Archosauria; Dinosauria; Saurischia; Theropoda; Coelurosauria; Aves; Neognathae; Gruiformes; Mesitornithidae; Mesitornis.<br>Chromosome: Unknown, Exon count: >9 | cd08190<br>KFQ38188<br>(ADHFE1)<br>350aa (fragment) |
| *Caprimulgus carolinensis* (chuck-will's-widow)<br>Eukaryota; Metazoa; Chordata; Craniata; Vertebrata; Euteleostomi; Archelosauria; Archosauria; Dinosauria; Saurischia; Theropoda; Coelurosauria; Aves; Neognathae; Caprimulgiformes; Caprimulgidae; Caprimulginae; Caprimulgus.<br>Chromosome: Unknown, Exon count: 14 | cd08190<br>XP_010176049<br>(ADHFE1)<br>419aa |
| *Buceros rhinoceros silvestris*<br>Eukaryota; Metazoa; Chordata; Craniata; Vertebrata; Euteleostomi; Archelosauria; Archosauria; Dinosauria; Saurischia; Theropoda; Coelurosauria; Aves; Neognathae; Bucerotiformes; Bucerotidae; Buceros<br>Chromosome Unknown, exon count: 12 | cd08190<br>XP_010139145<br>(ADHFE1)<br>419 aa |
| *Nestor notabilis* (Kea)<br>Eukaryota; Metazoa; Chordata; Craniata; Vertebrata; Euteleostomi; Archelosauria; Archosauria; Dinosauria; Saurischia; Theropoda; Coelurosauria; Aves; Neognathae; Psittaciformes; Psittacidae; Nestor.<br>Chromosome: Unknown, Exon count: 13 | cd08190<br>XP_010011946<br>(ADHFE1)<br>419aa |
| *Pygoscelis adeliae* (Adelie penguin)<br>Eukaryota; Metazoa; Chordata; Craniata; Vertebrata; Euteleostomi; Archelosauria; Archosauria; Dinosauria; Saurischia; Theropoda; Coelurosauria; Aves; Neognathae; Sphenisciformes; Spheniscidae; Pygoscelis.<br>Chromosome: Unknown, Exon count: 14 | cd08190<br>XP_009333362<br>(ADHFE1)<br>474aa |
| *Aptenodytes forsteri* (emperor penguin)<br>Eukaryota; Metazoa; Chordata; Craniata; Vertebrata; Euteleostomi; Archelosauria; Archosauria; Dinosauria; Saurischia; Theropoda; Coelurosauria; Aves; Neognathae; Sphenisciformes; Spheniscidae; Aptenodytes<br>Chromosome: Unknown, Exon count: 14 | cd08190<br>XP_009274989<br>(ADHFE1)<br>419aa |
| *Columba livia* (rock pigeon)<br>Eukaryota; Metazoa; Chordata; Craniata; Vertebrata; Euteleostomi; Archelosauria; Archosauria; Dinosauria; Saurischia; Theropoda; Coelurosauria; Aves; Neognathae; Columbiformes; Columbidae; Columba.<br>Chromosome: Unknown, Exon count: 14 | cd08190<br>XP_005506575<br>(ADHFE1)<br>466aa |
| *Calypte anna* (Anna's hummingbird)<br>Eukaryota; Metazoa; Chordata; Craniata; Vertebrata; Euteleostomi; Archelosauria; Archosauria; Dinosauria; Saurischia; Theropoda; Coelurosauria; Aves; Neognathae; Trochiliformes; Trochilidae; Calypte.<br>Chromosome: Unknown, Exon count: 15 | cd08190<br>XP_008499982<br>(ADHFE1)<br>466aa |
| *Amazona aestiva* (blue-fronted amazon)<br>Eukaryota; Metazoa; Chordata; Craniata; Vertebrata; Euteleostomi; Archelosauria; Archosauria; Dinosauria; Saurischia; Theropoda; Coelurosauria; Aves; Neognathae; Psittaciformes; Psittacidae; Amazona<br>Chromosome: Unknown, Exon count: 11 | cd08190<br>KQK76336<br>(ADHFE1)<br>(AAES_136432)<br>419 aa |
| *Apaloderma vittatum* (bar-tailed trogon)<br>Eukaryota; Metazoa; Chordata; Craniata; Vertebrata; Euteleostomi; Archelosauria; Archosauria; Dinosauria; Saurischia; Theropoda; Coelurosauria; Aves; Neognathae; Trogoniformes; Trogonidae; Apaloderma<br>Chromosome: Unknown, Exon count: 13 | cd08190<br>XP_009863349<br>(ADHFE1)<br>418 aa |
| *Meleagris gallopavo* (turkey)<br>Eukaryota; Metazoa; Chordata; Craniata; Vertebrata; Euteleostomi; Archelosauria; Archosauria; Dinosauria; Saurischia; Theropoda; Coelurosauria; Aves; Neognathae; Galloanserae; Galliformes; Phasianidae; Meleagridinae; Meleagri<br>Chromosome: Unknown, Exon count: 13 | cd08190<br>XP_003205137-XP010707303<br>(ADHFE1)<br>470 aa |
| Mammalia | |

| Organism[1]<br>Lineage<br>Chromosome location and exon count | Fe-ADH subfamily (conserved Domain Database)<br>Protein accesion number<br>(Gene locus)<br>Amino acid sequence lenght |
|---|---|
| *Ornithorhynchus anatinus* (platypus)<br>Eukaryota; Metazoa; Chordata; Craniata; Vertebrata; Euteleostomi; Mammalia; Monotremata; Ornithorhynchidae; Ornithorhynchus<br>Chromosome: 7, Exon count: 14 | cd08190<br>XP_007665155<br>(ADHFE1)<br>452 aa |
| *Sarcophilus harrisii* (Tasmanian devil)<br>Eukaryota; Metazoa; Chordata; Craniata; Vertebrata; Euteleostomi; Mammalia; Metatheria; Dasyuromorphia; Dasyuridae; Sarcophilus.<br>Chromosome: 2, Exon count: 14 | cd08190<br>XP_003759787<br>(ADHFE1)<br>467aa |
| *Monodelphis domestica* (gray short-tailed opossum)<br>Eukaryota; Metazoa; Chordata; Craniata; Vertebrata; Euteleostomi; Mammalia; Metatheria; Didelphimorphia; Didelphidae; Monodelphis.<br>Chromosome: 3, Exon count: 15 | cd08190<br>XP_007487099<br>(ADHFE1)<br>467aa |
| *Dipodomys ordii* (Ord's kangaroo rat)<br>Eukaryota; Metazoa; Chordata; Craniata; Vertebrata; Euteleostomi; Mammalia; Eutheria; Euarchontoglires; Glires; Rodentia; Sciurognathi; Heteromyidae; Dipodomyinae; Dipodomys.<br>Chromosome: Unknown, Exon count: 14 | cd08190<br>XP_012889925<br>(ADHFE1)<br>466aa |
| *Dasypus novemcinctus* (nine-banded armadillo)<br>Eukaryota; Metazoa; Chordata; Craniata; Vertebrata; Euteleostomi; Mammalia; Eutheria; Xenarthra; Cingulata; Dasypodidae; Dasypus.<br>Chromosome: Unknown, Exon count: 13 | cd08190<br>XP_004477845<br>(ADHFE1)<br>513aa |
| *Tupaia chinensis* (Chinese tree shrew)<br>Eukaryota; Metazoa; Chordata; Craniata; Vertebrata; Euteleostomi; Mammalia; Eutheria; Euarchontoglires; Scandentia; Tupaiidae; Tupaia.<br>Chromosome: Unknown, Exon count: 13 | cd08190<br>XP_014444069<br>(ADHFE1)<br>466aa |
| *Marmota marmota marmota* (Alpine marmot)<br>Eukaryota; Metazoa; Chordata; Craniata; Vertebrata; Euteleostomi; Mammalia; Eutheria; Euarchontoglires; Glires; Rodentia; Sciurognathi; Sciuridae; Xerinae; Marmotini; Marmota.<br>Chromosome: Unknown, Exon count: 14 | cd08190<br>XP_015341313<br>(ADHFE1)<br>467aa |
| *Trichechus manatus latirostris* (Florida manatee)<br>Eukaryota; Metazoa; Chordata; Craniata; Vertebrata; Euteleostomi; Mammalia; Eutheria; Afrotheria; Sirenia; Trichechidae; Trichechus.<br>Chromosome: Unknown, Exon count: >12 | cd08190<br>XP_004372658<br>(ADHFE1)<br>434aa (fragment) |
| *Loxodonta africana* (African savanna elephant)<br>Eukaryota; Metazoa; Chordata; Craniata; Vertebrata; Euteleostomi; Mammalia; Eutheria; Afrotheria; Proboscidea; Elephantidae; Loxodonta.<br>Chromosome: Unknown, Exon count: 14 | cd08190<br>XP_003408255<br>(ADHFE1)<br>455aa |
| *Elephantulus edwardii* (Cape elephant shrew)<br>Eukaryota; Metazoa; Chordata; Craniata; Vertebrata; Euteleostomi; Mammalia; Eutheria; Afrotheria; Macroscelidea; Macroscelididae; Elephantulus.<br>Chromosome: Unknown Exon count: 14 | cd08190<br>XP_006880489<br>(ADHFE1)<br>419aa |
| *Chrysochloris asiatica* (Cape golden mole)<br>Eukaryota; Metazoa; Chordata; Craniata; Vertebrata; Euteleostomi; Mammalia; Eutheria; Afrotheria; Chrysochloridae; Chrysochlorinae; Chrysochloris.<br>Chromosome: Unknown, Exon count: 14 | cd08190<br>XP_006860107<br>(ADHFE1)<br>467aa |
| *Orycteropus afer afer*<br>Eukaryota; Metazoa; Chordata; Craniata; Vertebrata; Euteleostomi; Mammalia; Eutheria; Afrotheria; Tubulidentata; Orycteropodidae; Orycteropus.<br>Chromosome: Unknown, Exon count: 14 | cd08190<br>XP_007947917<br>(ADHFE1)<br>467aa |
| *Echinops telfairi* (small Madagascar hedgehog)<br>Eukaryota; Metazoa; Chordata; Craniata; Vertebrata; Euteleostomi; Mammalia; Eutheria; Afrotheria; Tenrecidae; Tenrecinae; Echinops<br>Chromosome: Unknown, Exon count: 14 | cd08190<br>XP_004697595<br>(ADHFE1)<br>466 aa (gen sequence corrected from XM004697538) |
| *Pan troglodytes* (chimpanzee)<br>Eukaryota; Metazoa; Chordata; Craniata; Vertebrata; Euteleostomi; Mammalia; Eutheria; Euarchontoglires; Primates; Haplorrhini; Catarrhini; Hominidae; Pan<br>Chromosome Unknown, exon count: Unknown | cd08190<br>XP_009453861<br>(ADHFE1)<br>481 aa |
| *Pan paniscus* (pygmy_chimpanzee)<br>Eukaryota; Metazoa; Chordata; Craniata; Vertebrata; Euteleostomi; Mammalia; Eutheria; Euarchontoglires; Primates; Haplorrhini; Catarrhini; Hominidae; Pan<br>Chromosome 8, exon count: 14 | cd08190<br>XP_003831412<br>(ADHFE1)<br>467 aa |
| *Gorilla gorilla* (western lowland gorilla)<br>Eukaryota; Metazoa; Chordata; Craniata; Vertebrata; Euteleostomi; Mammalia; Eutheria; Euarchontoglires; Primates; Haplorrhini; Catarrhini; Hominidae; Gorilla<br>Chromosome 8, exon count: 14 | cd08190<br>XP_004047159<br>(ADHFE1)<br>467 aa |

| Organism[1]<br>Lineage<br>Chromosome location and exon count | Fe-ADH subfamily (conserved Domain Database)<br>Protein accesion number<br>(Gene locus)<br>Amino acid sequence lenght |
|---|---|
| *Homo sapiens* (human)<br>Eukaryota; Metazoa; Chordata; Craniata; Vertebrata; Euteleostomi; Mammalia; Eutheria; Euarchontoglires; Primates; Haplorrhini; Catarrhini; Hominidae; Homo<br>Chromosome 8, exon count: 14 | cd08190<br>NP_653251<br>NP_001071061<br>Q8IWW8<br>(ADHFE1)<br>467 aa |
| *Pongo abelii* (Sumatran_orangutan)<br>Eukaryota; Metazoa; Chordata; Craniata; Vertebrata; Euteleostomi; Mammalia; Eutheria; Euarchontoglires; Primates; Haplorrhini; Catarrhini; Hominidae; Pongo.<br>Chromosome 8, exon count: 14 | cd08190<br>Q5RF11<br>NP_001128888<br>(ADHFE1)<br>467 aa |
| *Nomascus leucogenys* (northern white-cheeked gibbon)<br>Eukaryota; Metazoa; Chordata; Craniata; Vertebrata; Euteleostomi; Mammalia; Eutheria; Euarchontoglires; Primates; Haplorrhini; Catarrhini; Hylobatidae; Nomascus<br>Chromosome 16, exon count: 14 | cd08190<br>XP_003274823<br>(ADHFE1)<br>467 aa |
| *Rhinopithecus roxellana* (golden snub-nosed monkey)<br>Eukaryota; Metazoa; Chordata; Craniata; Vertebrata; Euteleostomi; Mammalia; Eutheria; Euarchontoglires; Primates; Haplorrhini; Catarrhini; Cercopithecidae; Colobinae; Rhinopithecus<br>Chromosome Unknown, exon count: 14 | cd08190<br>XP_010369880<br>(ADHFE1)<br>467 aa |
| *Chlorocebus sabaeus* (green monkey)<br>Eukaryota; Metazoa; Chordata; Craniata; Vertebrata; Euteleostomi; Mammalia; Eutheria; Euarchontoglires; Primates; Haplorrhini; Catarrhini; Cercopithecidae; Cercopithecinae; Chlorocebus<br>Chromosome 8, exon count: 14 | cd08190<br>XP_007998962<br>(ADHFE1)<br>467 aa |
| *Colobus angolensis palliatus*<br>Eukaryota; Metazoa; Chordata; Craniata; Vertebrata; Euteleostomi; Mammalia; Eutheria; Euarchontoglires; Primates; Haplorrhini; Catarrhini; Cercopithecidae; Colobinae; Colobus<br>Chromosome Unknown, exon count: 14 | cd08190<br>XP_011796278<br>(ADHFE1)<br>467 aa |
| *Papio anubis* (olive_baboon)<br>Eukaryota; Metazoa; Chordata; Craniata; Vertebrata; Euteleostomi; Mammalia; Eutheria; Euarchontoglires; Primates; Haplorrhini; Catarrhini; Cercopithecidae; Cercopithecinae; Papio<br>Chromosome 8, exon count: 15 | cd08190<br>XP_003902867<br>(ADHFE1)<br>467 aa |
| *Cercocebus atys* (sooty mangabey)<br>Eukaryota; Metazoa; Chordata; Craniata; Vertebrata; Euteleostomi; Mammalia; Eutheria; Euarchontoglires; Primates; Haplorrhini; Catarrhini; Cercopithecidae; Cercopithecinae; Cercocebus<br>Chromosome Unknown, exon count: 14 | cd08190<br>XP_011914039<br>(ADHFE1)<br>467 aa |
| *Mandrillus leucophaeus* (drill)<br>Eukaryota; Metazoa; Chordata; Craniata; Vertebrata; Euteleostomi; Mammalia; Eutheria; Euarchontoglires; Primates; Haplorrhini; Catarrhini; Cercopithecidae; Cercopithecinae; Mandrillus<br>Chromosome Unknown, exon count: 14 | cd08190<br>XP_011831862<br>(ADHFE1)<br>467 aa |
| *Macaca fascicularis* (crab-eating macaque)<br>Eukaryota; Metazoa; Chordata; Craniata; Vertebrata; Euteleostomi; Mammalia; Eutheria; Euarchontoglires; Primates; Haplorrhini; Catarrhini; Cercopithecidae; Cercopithecinae; Macaca.<br>Chromosome 8, exon count: 14 | cd08190<br>XP_005563504<br>EHH64206<br>(ADHFE1)<br>467 aa |
| *Macaca nemestrina* (pig-tailed_macaque)<br>Eukaryota; Metazoa; Chordata; Craniata; Vertebrata; Euteleostomi; Mammalia; Eutheria; Euarchontoglires; Primates; Haplorrhini; Catarrhini; Cercopithecidae; Cercopithecinae; Macaca.<br>Chromosome Unknown, exon count: 17 | cd08190<br>XP_011740484<br>(ADHFE1)<br>467 aa |
| *Macaca mulatta* (Rhesus monkey)<br>Eukaryota; Metazoa; Chordata; Craniata; Vertebrata; Euteleostomi; Mammalia; Eutheria; Euarchontoglires; Primates; Haplorrhini; Catarrhini; Cercopithecidae; Cercopithecinae; Macaca.<br>Chromosome 8, exon count: 16 | cd08190<br>XP_001094680<br>EHH28543<br>(ADHFE1)<br>467 aa |
| *Aotus nancymaae* (Ma's_night_monkey)<br>Eukaryota; Metazoa; Chordata; Craniata; Vertebrata; Euteleostomi; Mammalia; Eutheria; Euarchontoglires; Primates; Haplorrhini; Platyrrhini; Aotidae; Aotus<br>Chromosome Unknown, exon count: 14 | cd08190<br>XP_012319117<br>(ADHFE1)<br>467 aa |
| *Callithrix jacchus* (white-tufted-ear marmoset)<br>Eukaryota; Metazoa; Chordata; Craniata; Vertebrata; Euteleostomi; Mammalia; Eutheria; Euarchontoglires; Primates; Haplorrhini; Platyrrhini; Cebidae; Callitrichinae; Callithrix<br>Chromosome 16, exon count: 15 | cd08190<br>XP_002759026<br>(ADHFE1)<br>467 aa |

| Organism[1]<br>Lineage<br>Chromosome location and exon count | Fe-ADH subfamily (conserved Domain Database)<br>Protein accesion number<br>(Gene locus)<br>Amino acid sequence lenght |
|---|---|
| *Saimiri boliviensis boliviensis* (Bolivian squirrel monkey)<br>Eukaryota; Metazoa; Chordata; Craniata; Vertebrata; Euteleostomi; Mammalia; Eutheria; Euarchontoglires; Primates; Haplorrhini; Platyrrhini; Cebidae; Saimiriinae; Saimiri<br>Chromosome Unknown, exon count: 14 | cd08190<br>XP_003942935<br>(ADHFE1)<br>467 aa |
| *Otolemur garnettii* (small-eared galago)<br>Eukaryota; Metazoa; Chordata; Craniata; Vertebrata; Euteleostomi; Mammalia; Eutheria; Euarchontoglires; Primates; Strepsirrhini; Lorisiformes; Galagidae; Otolemur<br>Chromosome Unknown, exon count: 12 | cd08190<br>XP_012665272<br>XP_003797287<br>(ADHFE1)<br>419 aa |
| *Propithecus coquereli* (Coquerel's sifaka)<br>Eukaryota; Metazoa; Chordata; Craniata; Vertebrata; Euteleostomi; Mammalia; Eutheria; Euarchontoglires; Primates; Strepsirrhini; Lemuriformes; Indriidae; Propithecus<br>Chromosome Unknown, exon count: 13 | cd08190<br>XP_012518621<br>(ADHFE1)<br>419 aa |
| *Microcebus murinus* (gray mouse lemur)<br>Eukaryota; Metazoa; Chordata; Craniata; Vertebrata; Euteleostomi; Mammalia; Eutheria; Euarchontoglires; Primates; Strepsirrhini; Lemuriformes; Cheirogaleidae; Microcebus<br>Chromosome Unknown, exon count: 15 | cd08190<br>XP_012592635<br>(ADHFE1)<br>467 aa |
| *Condylura cristata* (star-nosed mole)<br>Eukaryota; Metazoa; Chordata; Craniata; Vertebrata; Euteleostomi; Mammalia; Eutheria; Laurasiatheria; Insectivora; Talpidae; Condylura<br>Chromosome Unknown, exon count: 14 | cd08190<br>XP_004679887<br>(ADHFE1)<br>467 aa |
| *Ochotona princeps* (American pika)<br>Eukaryota; Metazoa; Chordata; Craniata; Vertebrata; Euteleostomi; Mammalia; Eutheria; Euarchontoglires; Glires; Lagomorpha; Ochotonidae; Ochotona.<br>Chromosome Unknown, exon count: 12 | cd08190<br>XP_004588158<br>(ADHFE1)<br>461aa |
| *Tarsius syrichta* (Philippine tarsier)<br>Eukaryota; Metazoa; Chordata; Craniata; Vertebrata; Euteleostomi; Mammalia; Eutheria; Euarchontoglires; Primates; Haplorrhini; Tarsiiformes; Tarsiidae; Tarsius<br>Chromosome Unknown, exon count: 12 | cd08190<br>XP_008072504<br>(ADHFE1)<br>419 aa |
| *Oryctolagus cuniculus* (rabbit)<br>Eukaryota; Metazoa; Chordata; Craniata; Vertebrata; Euteleostomi; Mammalia; Eutheria; Euarchontoglires; Glires; Lagomorpha; Leporidae; Oryctolagus.<br>Chromosome 3, exon count: 12 | cd08190<br>XP_008253810<br>(ADHFE1)<br>466aa |
| *Rousettus aegyptiacus* (Egyptian rousette)<br>Eukaryota; Metazoa; Chordata; Craniata; Vertebrata; Euteleostomi; Mammalia; Eutheria; Laurasiatheria; Chiroptera; Megachiroptera; Pteropodidae; Pteropodinae; Rousettus<br>Chromosome Unknown, exon count: 14 | cd08190<br>XP_016008386<br>(ADHFE1)<br>467 aa |
| *Pteropus alecto* (black flying fox)<br>Eukaryota; Metazoa; Chordata; Craniata; Vertebrata; Euteleostomi; Mammalia; Eutheria; Laurasiatheria; Chiroptera; Megachiroptera; Pteropodidae; Pteropodinae; Pteropus<br>Chromosome Unknown, exon count: 14 | cd08190<br>XP_015454185<br>(ADHFE1)<br>467 aa |
| *Pteropus vampyrus* (large flying fox)<br>Eukaryota; Metazoa; Chordata; Craniata; Vertebrata; Euteleostomi; Mammalia; Eutheria; Laurasiatheria; Chiroptera; Megachiroptera; Pteropodidae; Pteropodinae; Pteropus<br>Chromosome Unknown, exon count: 17 | cd08190<br>XP_011362925<br>(ADHFE1)<br>551 aa |
| *Eptesicus fuscus* (big brown bat)<br>Eukaryota; Metazoa; Chordata; Craniata; Vertebrata; Euteleostomi; Mammalia; Eutheria; Laurasiatheria; Chiroptera; Microchiroptera; Vespertilionidae; Eptesicus.<br>Chromosome Unknown, exon count: 14 | cd08190<br>XP_008148595<br>(ADHFE1)<br>466aa |
| *Myotis lucifugus* (little brown bat)<br>Eukaryota; Metazoa; Chordata; Craniata; Vertebrata; Euteleostomi; Mammalia; Eutheria; Laurasiatheria; Chiroptera; Microchiroptera; Vespertilionidae; Myotis<br>Chromosome Unknown, exon count: 19 | cd08190<br>XP_006090674<br>(ADHFE1)<br>466 aa |
| *Myotis brandtii* (Brandt's_bat)<br>Eukaryota; Metazoa; Chordata; Craniata; Vertebrata; Euteleostomi; Mammalia; Eutheria; Laurasiatheria; Chiroptera; Microchiroptera; Vespertilionidae; Myotis<br>Chromosome Unknown, exon count: 16 | cd08190<br>EPQ07854<br>(ADHFE1)<br>444 aa (fragment) |
| *Myotis davidii*<br>Eukaryota; Metazoa; Chordata; Craniata; Vertebrata; Euteleostomi; Mammalia; Eutheria; Laurasiatheria; Chiroptera; Microchiroptera; Vespertilionidae; Myotis<br>Chromosome Unknown, exon count: 18 | cd08190<br>XP_006756981<br>(ADHFE1)<br>466 aa |
| *Cricetulus griseus* (Chinese hamster)<br>Eukaryota; Metazoa; Chordata; Craniata; Vertebrata; Euteleostomi; Mammalia; Eutheria; Euarchontoglires; Glires; Rodentia; Sciurognathi; Muroidea; Cricetidae; Cricetinae; Cricetulus.<br>Chromosome Unknown, exon count: 14 | cd08190<br>XP_007634255<br>(ADHFE1)<br>467aa |

| Organism[1]<br>Lineage<br>Chromosome location and exon count | Fe-ADH subfamily (conserved Domain Database)<br>Protein accesion number<br>(Gene locus)<br>Amino acid sequence lenght |
|---|---|
| *Microtus ochrogaster* (prairie vole)<br>Eukaryota; Metazoa; Chordata; Craniata; Vertebrata; Euteleostomi; Mammalia; Eutheria; Euarchontoglires; Glires; Rodentia; Sciurognathi; Muroidea; Cricetidae; Arvicolinae; Microtus.<br>Chromosome Unknown, exon count: 14 | cd08190<br>XP_005361952<br>(ADHFE1)<br>467aa |
| *Mesocricetus auratus* (golden hamster)<br>Eukaryota; Metazoa; Chordata; Craniata; Vertebrata; Euteleostomi; Mammalia; Eutheria; Euarchontoglires; Glires; Rodentia; Sciurognathi; Muroidea; Cricetidae; Cricetinae; Mesocricetus.<br>Chromosome Unknown, exon count: 14 | cd08190<br>XP_005066801<br>(ADHFE1)<br>467aa |
| *Peromyscus maniculatus bairdii* (prairie deer mouse)<br>Eukaryota; Metazoa; Chordata; Craniata; Vertebrata; Euteleostomi; Mammalia; Eutheria; Euarchontoglires; Glires; Rodentia; Sciurognathi; Muroidea; Cricetidae; Neotominae; Peromyscus.<br>Chromosome Unknown exon count: 14 | cd08190<br>XP_006974739<br>(ADHFE1)<br>467 aa |
| *Rattus norvegicus* (Norway rat)<br>Eukaryota; Metazoa; Chordata; Craniata; Vertebrata; Euteleostomi; Mammalia; Eutheria; Euarchontoglires; Glires; Rodentia; Sciurognathi; Muroidea; Muridae; Murinae; Rattus<br>Chromosome 5, exon count: 16 | cd08190<br>Q4QQW3<br>NP_001020594<br>(ADHFE1)<br>467 aa |
| *Mus musculus* (house mouse)<br>Eukaryota; Metazoa; Chordata; Craniata; Vertebrata; Euteleostomi; Mammalia; Eutheria; Euarchontoglires; Glires; Rodentia; Sciurognathi; Muroidea; Muridae; Murinae; Mus; Mus<br>Chromosome 1, exon count: 15 | cd08190<br>Q8R0N6<br>NP_780445<br>(ADHFE1)<br>465 aa |
| *Nannospalax galili* (Upper Galilee mountains blind mole rat)<br>Eukaryota; Metazoa; Chordata; Craniata; Vertebrata; Euteleostomi; Mammalia; Eutheria; Euarchontoglires; Glires; Rodentia; Sciurognathi; Muroidea; Spalacidae; Spalacinae; Nannospalax.<br>Chromosome Unknown, exon count: 14 | cd08190<br>XP_008852984<br>(ADHFE1)<br>467aa |
| *Jaculus jaculus* (lesser Egyptian jerboa)<br>Eukaryota; Metazoa; Chordata; Craniata; Vertebrata; Euteleostomi; Mammalia; Eutheria; Euarchontoglires; Glires; Rodentia; Sciurognathi; Dipodidae; Dipodinae; Jaculus.<br>Chromosome Unknown, exon count: 14 | cd08190<br>XP_004653368<br>(ADHFE1)<br>467aa |
| *Fukomys damarensis* (Damara mole-rat)<br>Eukaryota; Metazoa; Chordata; Craniata; Vertebrata; Euteleostomi; Mammalia; Eutheria; Euarchontoglires; Glires; Rodentia; Hystricognathi; Bathyergidae; Fukomys.<br>Chromosome Unknown, exon count: 15 | cd08190<br>XP_010609784<br>(ADHFE1)<br>475aa |
| *Octodon degus* (degu)<br>Eukaryota; Metazoa; Chordata; Craniata; Vertebrata; Euteleostomi; Mammalia; Eutheria; Euarchontoglires; Glires; Rodentia; Hystricognathi; Octodontidae; Octodon.<br>Chromosome Unknown, exon count: 14 | cd08190<br>XP_004637582<br>(ADHFE1)<br>467aa |
| *Chinchilla lanigera* (long-tailed chinchilla)<br>Eukaryota; Metazoa; Chordata; Craniata; Vertebrata; Euteleostomi; Mammalia; Eutheria; Euarchontoglires; Glires; Rodentia; Hystricognathi; Chinchillidae; Chinchilla.<br>Chromosome Unknown, exon count: 13 | cd08190<br>XP_005392210<br>(ADHFE1)<br>467aa |
| *Heterocephalus glaber* (naked mole-rat)<br>Eukaryota; Metazoa; Chordata; Craniata; Vertebrata; Euteleostomi; Mammalia; Eutheria; Euarchontoglires; Glires; Rodentia; Hystricognathi; Bathyergidae; Heterocephalus.<br>Chromosome Unknown, exon count: 15 | cd08190<br>XP_004842165<br>(ADHFE1)<br>467aa |
| *Cavia porcellus* (domestic guinea pig)<br>Eukaryota; Metazoa; Chordata; Craniata; Vertebrata; Euteleostomi; Mammalia; Eutheria; Euarchontoglires; Glires; Rodentia; Hystricognathi; Caviidae; Cavia<br>Chromosome Unknown, exon count: 14 | cd08190<br>XP_003480118<br>(ADHFE1)<br>466 aa |
| *Ceratotherium simum simum* (southern white rhinoceros)<br>Eukaryota; Metazoa; Chordata; Craniata; Vertebrata; Euteleostomi; Mammalia; Eutheria; Laurasiatheria; Perissodactyla; Rhinocerotidae; Ceratotherium<br>Chromosome Unknown, exon count: 14 | cd08190<br>XP_004435805<br>(LOC101400099)<br>466 aa |
| *Mustela putorius furo* (domestic ferret)<br>Eukaryota; Metazoa; Chordata; Craniata; Vertebrata; Euteleostomi; Mammalia; Eutheria; Laurasiatheria; Carnivora; Caniformia; Mustelidae; Mustelinae; Mustela.<br>Chromosome Unknown, exon count: 16 | cd08190<br>XP_004773031<br>(ADHFE1)<br>469aa |
| *Equus asinus* (ass)<br>Eukaryota; Metazoa; Chordata; Craniata; Vertebrata; Euteleostomi; Mammalia; Eutheria; Laurasiatheria; Perissodactyla; Equidae; Equus.<br>Chromosome Unknown, exon count: 14 | cd08190<br>XP_014694005<br>(ADHFE1)<br>419aa |
| *Equus przewalskii* (Przewalski's horse)<br>Eukaryota; Metazoa; Chordata; Craniata; Vertebrata; Euteleostomi; Mammalia; Eutheria; Laurasiatheria; Perissodactyla; Equidae; Equus.<br>Chromosome Unknown, exon count: 12 | cd08190<br>XP_008524892<br>(ADHFE1)<br>419aa |

| Organism[1]<br>Lineage<br>Chromosome location and exon count | Fe-ADH subfamily (conserved Domain Database)<br>Protein accesion number<br>(Gene locus)<br>Amino acid sequence lenght |
|---|---|
| *Odobenus rosmarus divergens* (Pacific walrus)<br>Eukaryota; Metazoa; Chordata; Craniata; Vertebrata; Euteleostomi; Mammalia; Eutheria; Laurasiatheria; Carnivora; Caniformia; Odobenidae; Odobenus.<br>Chromosome Unknown, exon count: 14 | cd08190<br>XP_004402347<br>(ADHFE1)<br>467aa |
| *Leptonychotes weddellii* (Weddell seal)<br>Eukaryota; Metazoa; Chordata; Craniata; Vertebrata; Euteleostomi; Mammalia; Eutheria; Laurasiatheria; Carnivora; Caniformia; Phocidae; Leptonychotes.<br>Chromosome Unknown, exon count: 14 | cd08190<br>XP_006749542<br>(ADHFE1)<br>467aa |
| *Ursus maritimus* (polar bear)<br>Eukaryota; Metazoa; Chordata; Craniata; Vertebrata; Euteleostomi; Mammalia; Eutheria; Laurasiatheria; Carnivora; Caniformia; Ursidae; Ursus.<br>Chromosome Unknown, exon count: 14 | cd08190<br>XP_008704993<br>(ADHFE1)<br>457aa |
| *Ailuropoda melanoleuca* (giant panda)<br>Eukaryota; Metazoa; Chordata; Craniata; Vertebrata; Euteleostomi; Mammalia; Eutheria; Laurasiatheria; Carnivora; Caniformia; Ursidae; Ailuropoda.<br>Chromosome Unknown, exon count: 14 | cd08190<br>XP_002916417<br>(ADHFE1)<br>467aa |
| *Canis lupus familiaris* (dog)<br>Eukaryota; Metazoa; Chordata; Craniata; Vertebrata; Euteleostomi; Mammalia; Eutheria; Laurasiatheria; Carnivora; Caniformia; Canidae; Canis.<br>Chromosome 29, exon count: 15 | cd08190<br>XP_849448<br>(ADHFE1)<br>467aa |
| *Acinonyx jubatus* (cheetah)<br>Eukaryota; Metazoa; Chordata; Craniata; Vertebrata; Euteleostomi; Mammalia; Eutheria; Laurasiatheria; Carnivora; Feliformia; Felidae; Acinonychinae; Acinonyx<br>Chromosome Unknown, exon count: 14 | cd08190<br>XP_014933192<br>(ADHFE1)<br>467 aa |
| *Panthera tigris altaica* (Amur tiger)<br>Eukaryota; Metazoa; Chordata; Craniata; Vertebrata; Euteleostomi; Mammalia; Eutheria; Laurasiatheria; Carnivora; Feliformia; Felidae; Pantherinae; Panthera<br>Chromosome Unknown, exon count: 14 | cd08190<br>XP_007085083<br>(ADHFE1)<br>467 aa |
| *Equus caballus* (horse)<br>Eukaryota; Metazoa; Chordata; Craniata; Vertebrata; Euteleostomi; Mammalia; Eutheria; Laurasiatheria; Perissodactyla; Equidae; Equus.<br>Chromosome 9, exon count: 13 | cd08190<br>XP_001915713<br>(LOC100063782)<br>480aa |
| *Felis catus* (domestic cat)<br>Eukaryota; Metazoa; Chordata; Craniata; Vertebrata; Euteleostomi; Mammalia; Eutheria; Laurasiatheria; Carnivora; Feliformia; Felidae; Felinae; Felis<br>Chromosome F2, exon count: 14 | cd08190<br>XP_003999906<br>(ADHFE1)<br>467 aa |
| *Sorex araneus* (European shrew)<br>Eukaryota; Metazoa; Chordata; Craniata; Vertebrata; Euteleostomi; Mammalia; Eutheria; Laurasiatheria; Insectivora; Soricidae; Soricinae; Sorex<br>Chromosome Unknown, exon count: >13 | cd08190<br>XP_004602475<br>(ADHFE1)<br>445 aa (corrected fragment) |
| *Galeopterus variegatus* (Sunda flying lemur)<br>Eukaryota; Metazoa; Chordata; Craniata; Vertebrata; Euteleostomi; Mammalia; Eutheria; Euarchontoglires; Dermoptera; Cynocephalidae; Galeopterus.<br>Chromosome Unknown, exon count: 15 | cd08190<br>XP_008573951<br>(ADHFE1)<br>419aa |
| *Sus scrofa* (pig)<br>Eukaryota; Metazoa; Chordata; Craniata; Vertebrata; Euteleostomi; Mammalia; Eutheria; Laurasiatheria; Cetartiodactyla; Suina; Suidae; Sus.<br>Chromosome 4, exon count: 14 | cd08190<br>XP_003125651<br>(ADHFE1)<br>467aa |
| *Camelus ferus* (Wild Bactrian camel)<br>Eukaryota; Metazoa; Chordata; Craniata; Vertebrata; Euteleostomi; Mammalia; Eutheria; Laurasiatheria; Cetartiodactyla; Tylopoda; Camelidae; Camelus.<br>Chromosome Unknown, exon count: 16 | cd08190<br>XP_014422386<br>(ADHFE1)<br>478aa |
| *Camelus bactrianus* (Bactrian camel)<br>Eukaryota; Metazoa; Chordata; Craniata; Vertebrata; Euteleostomi; Mammalia; Eutheria; Laurasiatheria; Cetartiodactyla; Tylopoda; Camelidae; Camelus.<br>Chromosome Unknown, exon count: 16 | cd08190<br>XP_010968945<br>(ADHFE1)<br>478aa |
| *Vicugna pacos* (alpaca)<br>Eukaryota; Metazoa; Chordata; Craniata; Vertebrata; Euteleostomi; Mammalia; Eutheria; Laurasiatheria; Cetartiodactyla; Tylopoda; Camelidae; Vicugna.<br>Chromosome Unknown, exon count: 16 | cd08190<br>XP_006204760<br>(ADHFE1)<br>457aa |
| *Camelus dromedarius* (Arabian camel)<br>Eukaryota; Metazoa; Chordata; Craniata; Vertebrata; Euteleostomi; Mammalia; Eutheria; Laurasiatheria; Cetartiodactyla; Tylopoda; Camelidae; Camelus.<br>Chromosome Unknown, exon count: 13 | cd08190<br>XP_010986162<br>(ADHFE1)<br>467aa |
| *Tursiops truncatus* (bottlenosed dolphin)<br>Eukaryota; Metazoa; Chordata; Craniata; Vertebrata; Euteleostomi; Mammalia; Eutheria; Laurasiatheria; Cetartiodactyla; Cetacea; Odontoceti; Delphinidae; Tursiops.<br>Chromosome Unknown, exon count: 14 | cd08190<br>XP_004326596<br>(ADHFE1)<br>466aa |
| *Orcinus orca* (killer whale)<br>Eukaryota; Metazoa; Chordata; Craniata; Vertebrata; Euteleostomi; Mammalia; Eutheria; Laurasiatheria; Cetartiodactyla; Cetacea; Odontoceti; Delphinidae; Orcinus.<br>Chromosome Unknown, exon count: 14 | cd08190<br>XP_004275009<br>(ADHFE1)<br>466aa |

| Organism[1]<br>Lineage<br>Chromosome location and exon count | Fe-ADH subfamily (conserved Domain Database)<br>Protein accesion number<br>(Gene locus)<br>Amino acid sequence lenght |
|---|---|
| *Physeter catodon* (sperm whale)<br>Eukaryota; Metazoa; Chordata; Craniata; Vertebrata; Euteleostomi; Mammalia; Eutheria; Laurasiatheria; Cetartiodactyla; Cetacea; Odontoceti; Physeteridae; Physeter.<br>Chromosome Unknown, exon count: 15 | cd08190<br>XP_007119729<br>(ADHFE1)<br>466aa |
| *Balaenoptera acutorostrata scammoni*<br>Eukaryota; Metazoa; Chordata; Craniata; Vertebrata; Euteleostomi; Mammalia; Eutheria; Laurasiatheria; Cetartiodactyla; Cetacea; Mysticeti; Balaenopteridae; Balaenoptera.<br>Chromosome Unknown, exon count: 14 | cd08190<br>XP_007185067<br>(ADHFE1)<br>419aa |
| *Lipotes vexillifer* (Yangtze River dolphin)<br>Eukaryota; Metazoa; Chordata; Craniata; Vertebrata; Euteleostomi; Mammalia; Eutheria; Laurasiatheria; Cetartiodactyla; Cetacea; Odontoceti; Lipotidae; Lipotes.<br>Chromosome Unknown, exon count: 14 | cd08190<br>XP_007448540<br>(ADHFE1)<br>466aa |
| *Bubalus bubalis* (water buffalo)<br>Eukaryota; Metazoa; Chordata; Craniata; Vertebrata; Euteleostomi; Mammalia; Eutheria; Laurasiatheria; Cetartiodactyla; Ruminantia; Pecora; Bovidae; Bovinae; Bubalus.<br>Chromosome Unknown, exon count: 15 | cd08190<br>XP_006044636<br>(ADHFE1)<br>502aa |
| *Bos mutus* (wild yak)<br>Eukaryota; Metazoa; Chordata; Craniata; Vertebrata; Euteleostomi; Mammalia; Eutheria; Laurasiatheria; Cetartiodactyla; Ruminantia; Pecora; Bovidae; Bovinae; Bos<br>Chromosome Unknown, exon count: 14 | cd08190<br>XP_005893003<br>(ADHFE1)<br>466 aa |
| *Bos taurus* (cattle)<br>Eukaryota; Metazoa; Chordata; Craniata; Vertebrata; Euteleostomi; Mammalia; Eutheria; Laurasiatheria; Cetartiodactyla; Ruminantia; Pecora; Bovidae; Bovinae; Bos<br>Chromosome 14, exon count: 14 | cd08190<br>NP_001095357<br>XP_584372<br>(ADHFE1)<br>466 aa |
| *Ictidomys tridecemlineatus* (thirteen-lined ground squirrel)<br>Eukaryota; Metazoa; Chordata; Craniata; Vertebrata; Euteleostomi; Mammalia; Eutheria; Euarchontoglires; Glires; Rodentia; Sciurognathi; Sciuridae; Xerinae; Marmotini; Ictidomys.<br>Chromosome Unknown, exon count: 13 | cd08190<br>XP_005323004<br>(ADHFE1)<br>465aa |
| *Capra hircus* (goat)<br>Eukaryota; Metazoa; Chordata; Craniata; Vertebrata; Euteleostomi; Mammalia; Eutheria; Laurasiatheria; Cetartiodactyla; Ruminantia; Pecora; Bovidae; Caprinae; Capra.<br>Chromosome 14, exon count: 13 | cd08190<br>XP_005689087<br>(ADHFE1)<br>466aa |
| *Ovis aries musimon* (Ovis orientalis musimon)<br>Eukaryota; Metazoa; Chordata; Craniata; Vertebrata; Euteleostomi; Mammalia; Eutheria; Laurasiatheria; Cetartiodactyla; Ruminantia; Pecora; Bovidae; Caprinae; Ovis<br>Chromosome 9, exon count: 12 | cd08190<br>XP_011994307<br>(ADHFE1)<br>502 aa |
| *Pantholops hodgsonii* (chiru)<br>Eukaryota; Metazoa; Chordata; Craniata; Vertebrata; Euteleostomi; Mammalia; Eutheria; Laurasiatheria; Cetartiodactyla; Ruminantia; Pecora; Bovidae; Antilopinae; Pantholops.<br>Chromosome Unknown, exon count: 13 | cd08190<br>XP_005977655<br>(ADHFE1)<br>419aa |
| *Bison bison bison*<br>Eukaryota; Metazoa; Chordata; Craniata; Vertebrata; Euteleostomi; Mammalia; Eutheria; Laurasiatheria; Cetartiodactyla; Ruminantia; Pecora; Bovidae; Bovinae; Bison.<br>Chromosome Unknown, exon count: 13 | cd08190<br>XP_010846794<br>(ADHFE1)<br>457aa |
| *Ovis aries* (sheep)<br>Eukaryota; Metazoa; Chordata; Craniata; Vertebrata; Euteleostomi; Mammalia; Eutheria; Laurasiatheria; Cetartiodactyla; Ruminantia; Pecora; Bovidae; Caprinae; Ovis<br>Chromosome 9, exon count: 12 | cd08190<br>XP_014953274<br>(ADHFE1)<br>442 aa |

| Organism[1]<br>Lineage<br>Chromosome location and exon count | Fe-ADH family (conserved Domain Database)<br>Protein accesion number<br>(Gene locus)<br>Amino acid sequence lenght |
|---|---|
| Fungi | |
| Ascomycota | |
| *Diplodia seriata*<br>Eukaryota; Fungi; Dikarya; Ascomycota; Pezizomycotina; Dothideomycetes; Dothideomycetes incertae sedis; Botryosphaeriales; Botryosphaeriaceae; Diplodia<br>Chromosome Unknown, exon count: 3 (KKY15219)<br>Chromosome Unknown, exon count: 3 (KKY14168) | cd08177<br>KKY15219<br>(UCDDS831_g07760)<br>329aa<br><br>cd08190<br>KKY14168<br>(UCDDS831_g08383)<br>508 aa |
| *Macrophomina phaseolina* MS6<br>Eukaryota; Fungi; Dikarya; Ascomycota; Pezizomycotina; Dothideomycetes; Dothideomycetes incertae sedis; Botryosphaeriales; Botryosphaeriaceae; Macrophomina<br>Chromosome Unknown, exon count: 2 (EKG10069)<br>Chromosome Unknown, exon count: 3 (EKG12804) | cd08177<br>EKG10069<br>(MPH_12849)<br>378 aa<br><br>cd08190<br>EKG12804<br>(MPH_10047)<br>501 aa |
| *Pyrenophora teres f. teres* 0-1<br>Eukaryota; Fungi; Dikarya; Ascomycota; Pezizomycotina; Dothideomycetes; Pleosporomycetidae; Pleosporales; Pleosporineae; Pleosporaceae; Pyrenophora<br>Chromosome Unknown, exon count: 1 (XP_003296277) | cd08190<br>XP_003296277<br>(PTRG_06823)<br>494 aa |
| *Pyrenophora tritici-repentis* Pt-1C-BFP<br>Eukaryota; Fungi; Dikarya; Ascomycota; Pezizomycotina; Dothideomycetes; Pleosporomycetidae; Pleosporales; Pleosporineae; Pleosporaceae; Pyrenophora<br>Chromosome Unknown, exon count: 3 (XP_001936260)<br>Chromosome Unknown, exon count: 7 (XP_001938016)<br>Chromosome Unknown, exon count: 1 (XP_001937156) | cd08177<br>XP_001936260<br>(PTRG_05927)<br>552 aa (FeADH domain located at residues 1-475)<br><br>cd08177<br>XP_001938016<br>(PTRG_07684)<br>383 aa<br><br>cd08190<br>XP_001937156<br>(PTRG_06823)<br>494 aa |
| fungal sp. No.11243<br>Eukaryota; Fungi<br>Chromosome Unknown, exon count: 1 (GAM83494) | cd08190<br>GAM83494<br>(ANO11243_014820)<br>491 aa |
| *Bipolaris oryzae* ATCC 44560<br>Eukaryota; Fungi; Dikarya; Ascomycota; Pezizomycotina; Dothideomycetes; Pleosporomycetidae; Pleosporales; Pleosporineae; Pleosporaceae; Bipolaris<br>Chromosome Unknown, exon count: 1 (XP_007686952) | cd08190<br>XP_007686952<br>(COCMIDRAFT_25462)<br>494 aa |
| *Bipolaris maydis* ATCC 48331<br>Eukaryota; Fungi; Dikarya; Ascomycota; Pezizomycotina; Dothideomycetes; Pleosporomycetidae; Pleosporales; Pleosporineae; Pleosporaceae; Bipolaris<br>Chromosome Unknown, exon count: 12 (ENH99351)<br>Chromosome Unknown, exon count: 1 (XP_014073096) | cd08177<br>ENH99351<br>(COCC4DRAFT_85213)<br>919 aa (FeADH domain located at residues 1-375)<br><br>cd08190<br>XP_014073096<br>(COCC4DRAFT_153980)<br>494 aa |
| *Bipolaris maydis* C5<br>Eukaryota; Fungi; Dikarya; Ascomycota; Pezizomycotina; Dothideomycetes; Pleosporomycetidae; Pleosporales; Pleosporineae; Pleosporaceae; Bipolaris<br>Chromosome Unknown, exon count: 1 (EMD88543) | cd08190<br>EMD88543<br>(COCHEDRAFT_1142227)<br>494 aa |
| *Bipolaris victoriae* FI3 (*Cochliobolus victoriae* FI3)<br>Eukaryota; Fungi; Dikarya; Ascomycota; Pezizomycotina; Dothideomycetes; Pleosporomycetidae; Pleosporales; Pleosporineae; Pleosporaceae; Bipolaris<br>Chromosome Unknown, exon count: 1 (XP_014554696) | cd08190<br>XP_014554696<br>(COCVIDRAFT_28270)<br>494 aa |
| *Bipolaris zeicola* 26-R-13 (*Cochliobolus carbonum* 26-R-13)<br>Eukaryota; Fungi; Dikarya; Ascomycota; Pezizomycotina; Dothideomycetes; Pleosporomycetidae; Pleosporales; Pleosporineae; Pleosporaceae; Bipolaris<br>Chromosome Unknown, exon count: 1 (XP_007718106) | cd08190<br>XP_007718106<br>(COCCADRAFT_30954)<br>494 aa |

| Organism[1]<br>Lineage<br>Chromosome location and exon count | Fe-ADH family (conserved Domain Database)<br>Protein accesion number<br>(Gene locus)<br>Amino acid sequence lenght |
|---|---|
| *Bipolaris sorokiniana* ND90Pr (*Cochliobolus sativus* ND90Pr)<br>Eukaryota; Fungi; Dikarya; Ascomycota; Pezizomycotina; Dothideomycetes; Pleosporomycetidae; Pleosporales; Pleosporineae; Pleosporaceae; Bipolaris<br>Chromosome Unknown, exon count: 12 (EMD62832)<br>Chromosome Unknown, exon count: 1 (XP_007699877) | cd08177<br>EMD62832<br>(COCSADRAFT_200493)<br>938 aa (FeADH domain located at residues 1-375)<br><br>cd08190<br>XP_007699877<br>(COCSADRAFT_37131)<br>494 aa |
| *Setosphaeria turcica* Et28A<br>Eukaryota; Fungi; Dikarya; Ascomycota; Pezizomycotina; Dothideomycetes; Pleosporomycetidae; Pleosporales; Pleosporineae; Pleosporaceae; Setosphaeria<br>Chromosome Unknown, exon count: 1 (XP_008024327) | cd08190<br>XP_008024327<br>(SETTUDRAFT_107983)<br>494 aa |
| *Stemphylium lycopersici* CIDEFI-216<br>Eukaryota; Fungi; Dikarya; Ascomycota; Pezizomycotina; Dothideomycetes; Pleosporomycetidae; Pleosporales; Pleosporineae; Pleosporaceae; Stemphylium<br>Chromosome Unknown, exon count: 1 (KNG47877) | cd08190<br>KNG47877<br>(TW65_05376)<br>494 aa |
| *Leptosphaeria maculans JN3*<br>Eukaryota; Fungi; Dikarya; Ascomycota; Pezizomycotina; Dothideomycetes; Pleosporomycetidae; Pleosporales; Pleosporineae; Leptosphaeriaceae; Leptosphaeria; Leptosphaeria maculans complex<br>Chromosome Unknown, exon count: 13 (XP_003839285)<br>Chromosome Unknown, exon count: 2 (XP_003842490) | cd08177<br>XP_003839285<br>(LEMA_P029580.1)<br>990 aa (FeADH domain located at residues 1-375)<br><br>cd08190<br>XP_003842490<br>(LEMA_P082500.1)<br>610 aa (FeADH domain located at residues 116-610) |
| *Parastagonospora nodorum* SN15<br>Eukaryota; Fungi; Dikarya; Ascomycota; Pezizomycotina; Dothideomycetes; Pleosporomycetidae; Pleosporales; Pleosporineae; Phaeosphaeriaceae; Parastagonospora<br>Chromosome Unknown, exon count: 2 (XP_001803791)<br>Chromosome Unknown, exon count: 1 (XP_001803274) | Cd08177<br>XP_001803791<br>(SNOG_13584)<br>383 aa<br><br>cd08190<br>XP_001803274<br>(SNOG_13060)<br>425 aa |
| | |
| *Fonsecaea pedrosoi* CBS 271.37<br>Eukaryota; Fungi; Dikarya; Ascomycota; Pezizomycotina; Eurotiomycetes; Chaetothyriomycetidae; Chaetothyriales; Herpotrichiellaceae; Fonsecaea<br>Chromosome Unknown, exon count: 2 (XP_013284940)<br>Chromosome Unknown, exon count: 1 (XP_013283961) | cd08190<br>XP_013284940<br>(Z517_04155)<br>488 aa<br><br>cd08190<br>XP_013283961<br>(Z517_06768)<br>493 aa |
| *Fonsecaea multimorphosa* CBS 102226<br>Eukaryota; Fungi; Dikarya; Ascomycota; Pezizomycotina; Eurotiomycetes; Chaetothyriomycetidae; Chaetothyriales; Herpotrichiellaceae; Fonsecaea<br>Chromosome Unknown, exon count: 1 (KIX98432) | cd08190<br>KIX98432<br>(Z520_05733)<br>493 aa |
| *Capronia coronata* CBS 617.96<br>Eukaryota; Fungi; Dikarya; Ascomycota; Pezizomycotina; Eurotiomycetes; Chaetothyriomycetidae; Chaetothyriales; Herpotrichiellaceae; Capronia<br>Chromosome Unknown, exon count: 1 (XP_007725696)<br>Chromosome Unknown, exon count: 1 (XP_007722996) | cd08177<br>XP_007725696<br>(A1O1_06628)<br>417 aa<br><br>cd08190<br>XP_007722996<br>(A1O1_03907)<br>493 aa |
| *Capronia epimyces* CBS 606.96<br>Eukaryota; Fungi; Dikarya; Ascomycota; Pezizomycotina; Eurotiomycetes; Chaetothyriomycetidae; Chaetothyriales; Herpotrichiellaceae; Capronia<br>Chromosome Unknown, exon count: 1 (XP_007734780) | cd08190<br>XP_007734780<br>(A1O3_06471)<br>493 aa |
| *Capronia semi-immersa* (*Phialophora americana*)<br>Eukaryota; Fungi; Dikarya; Ascomycota; Pezizomycotina; Eurotiomycetes; Chaetothyriomycetidae; Chaetothyriales; Herpotrichiellaceae; Capronia<br>Chromosome Unknown, exon count: 1 (KIW63023) | cd08190<br>KIW63023<br>(PV04_09904)<br>493 aa |
| *Cladophialophora bantiana* CBS 173.52<br>Eukaryota; Fungi; Dikarya; Ascomycota; Pezizomycotina; Eurotiomycetes; Chaetothyriomycetidae; Chaetothyriales; Herpotrichiellaceae; Cladophialophora<br>Chromosome Unknown, exon count: 1 (KIW99194) | cd08190<br>KIW99194<br>(Z519_00857)<br>493 aa |

| Organism[1]<br>Lineage<br>Chromosome location and exon count | Fe-ADH family (conserved Domain Database)<br>Protein accesion number<br>(Gene locus)<br>Amino acid sequence lenght |
|---|---|
| *Cladophialophora carrionii* CBS 160.54<br>Eukaryota; Fungi; Dikarya; Ascomycota; Pezizomycotina; Eurotiomycetes; Chaetothyriomycetidae; Chaetothyriales; Herpotrichiellaceae; Cladophialophora<br>Chromosome Unknown, exon count: 1 (XP_008731740) | cd08190<br>XP_008731740<br>(G647_09214)<br>493 aa |
| *Cladophialophora immunda* CBS 83496<br>Eukaryota; Fungi; Dikarya; Ascomycota; Pezizomycotina; Eurotiomycetes; Chaetothyriomycetidae; Chaetothyriales; Herpotrichiellaceae; Cladophialophora<br>Chromosome Unknown, exon count: 1 (KIW29584) | cd08190<br>KIW29584<br>(PV07_05390)<br>493 aa |
| *Cladophialophora psammophila* CBS 110553<br>Eukaryota; Fungi; Dikarya; Ascomycota; Pezizomycotina; Eurotiomycetes; Chaetothyriomycetidae; Chaetothyriales; Herpotrichiellaceae; Cladophialophora<br>Chromosome Unknown, exon count: 1 (XP_007739731) | cd08190<br>XP_007739731<br>(A1O5_00922)<br>493 aa |
| *Cladophialophora yegresii* CBS 114405<br>Eukaryota; Fungi; Dikarya; Ascomycota; Pezizomycotina; Eurotiomycetes; Chaetothyriomycetidae; Chaetothyriales; Herpotrichiellaceae; Cladophialophora<br>Chromosome Unknown, exon count: 1 (XP_007761445) | cd08190<br>XP_007761445<br>(A1O7_09267)<br>493 aa |
| *Coniosporium apollinis* CBS 100218<br>Eukaryota; Fungi; Dikarya; Ascomycota; Pezizomycotina; Eurotiomycetes; Chaetothyriomycetidae; Chaetothyriales; Herpotrichiellaceae; Coniosporium<br>Chromosome Unknown, exon count: 2 (XP_007777870)<br>Chromosome Unknown, exon count: 2 (XP_007783426) | CD08177<br>XP_007777870<br>(W97_01777)<br>357 aa<br><br>cd08190<br>XP_007783426<br>(W97_07257)<br>496 aa |
| *Exophiala aquamarina* CBS 119918<br>Eukaryota; Fungi; Dikarya; Ascomycota; Pezizomycotina; Eurotiomycetes; Chaetothyriomycetidae; Chaetothyriales; Herpotrichiellaceae; Exophiala<br>Chromosome Unknown, exon count: 1 (XP_013255054) | cd08190<br>XP_013255054<br>(A1O9_11306)<br>493 aa |
| *Exophiala dermatitidis* NIH/UT8656<br>Eukaryota; Fungi; Dikarya; Ascomycota; Pezizomycotina; Eurotiomycetes; Chaetothyriomycetidae; Chaetothyriales; Herpotrichiellaceae; Exophiala<br>Chromosome Unknown, exon count: 1 (XP_009160820) | cd08190<br>XP_009160820<br>(HMPREF1120_08324)<br>493 aa |
| *Exophiala mesophila* CBS40295<br>Eukaryota; Fungi; Dikarya; Ascomycota; Pezizomycotina; Eurotiomycetes; Chaetothyriomycetidae; Chaetothyriales; Herpotrichiellaceae; Exophiala<br>Chromosome Unknown, exon count: 1 (KIV93161) | cd08190<br>KIV93161<br>(PV10_04399)<br>493 aa |
| *Exophiala oligosperma* CBS 72588<br>Eukaryota; Fungi; Dikarya; Ascomycota; Pezizomycotina; Eurotiomycetes; Chaetothyriomycetidae; Chaetothyriales; Herpotrichiellaceae; Exophiala<br>Chromosome Unknown, exon count: 1 (KIW41019) | cd08190<br>KIW41019<br>(PV06_06617)<br>493 aa |
| *Exophiala sideris* CBS121828<br>Eukaryota; Fungi; Dikarya; Ascomycota; Pezizomycotina; Eurotiomycetes; Chaetothyriomycetidae; Chaetothyriales; Herpotrichiellaceae; Exophiala<br>Chromosome Unknown, exon count: 1 (KIV83436) | cd08190<br>KIV83436<br>(PV11_05463)<br>493 aa |
| *Exophiala spinifera* CBS89968<br>Eukaryota; Fungi; Dikarya; Ascomycota; Pezizomycotina; Eurotiomycetes; Chaetothyriomycetidae; Chaetothyriales; Herpotrichiellaceae; Exophiala<br>Chromosome Unknown, exon count: 1 (KIW14704) | cd08190<br>KIV83436<br>(PV08_07488)<br>493 aa |
| *Exophiala xenobiotica* CBS 118157<br>Eukaryota; Fungi; Dikarya; Ascomycota; Pezizomycotina; Eurotiomycetes; Chaetothyriomycetidae; Chaetothyriales; Herpotrichiellaceae; Exophiala<br>Chromosome Unknown, exon count: 1 (XP_013320170) | cd08190<br>XP_013320170<br>(PV05_04028)<br>493 aa |
| *Phialophora attae*<br>Eukaryota; Fungi; Dikarya; Ascomycota; Pezizomycotina; Eurotiomycetes; Chaetothyriomycetidae; Chaetothyriales; Herpotrichiellaceae; Phialophora<br>Chromosome Unknown, exon count: 1 (KPI39934) | cd08190<br>KPI39934<br>(AB675_11606)<br>493 aa |
| *Rhinocladiella mackenziei* CBS 650.93<br>Eukaryota; Fungi; Dikarya; Ascomycota; Pezizomycotina; Eurotiomycetes; Chaetothyriomycetidae; Chaetothyriales; Herpotrichiellaceae; Rhinocladiella<br>Chromosome Unknown, exon count: 1 (XP_013270838) | cd08190<br>XP_013270838<br>(Z518_07255)<br>493 aa |
| *Cyphellophora europaea* CBS 101466 (*Phialophora europaea* CBS 101466)<br>Eukaryota; Fungi; Dikarya; Ascomycota; Pezizomycotina; Eurotiomycetes; Chaetothyriomycetidae; Chaetothyriales; Cyphellophoraceae; Cyphellophora<br>Chromosome Unknown, exon count: 1 (XP_008718007) | cd08190<br>XP_008718007<br>(HMPREF1541_05445)<br>493 aa |
| *Phaeomoniella chlamydospora*<br>Eukaryota; Fungi; Dikarya; Ascomycota; Pezizomycotina; Eurotiomycetes; Chaetothyriomycetidae; Phaeomoniellales; Phaeomoniellales incertae sedis; Phaeomoniella<br>Chromosome Unknown, exon count: >2 (KKY18931) | cd08190<br>KKY18931<br>(UCRPC4_g04691)<br>380 aa (fragment) |

| Organism[1]<br>Lineage<br>Chromosome location and exon count | Fe-ADH family (conserved Domain Database)<br>Protein accesion number<br>(Gene locus)<br>Amino acid sequence lenght |
|---|---|
| *Endocarpon pusillum* Z07020<br>Eukaryota; Fungi; Dikarya; Ascomycota; Pezizomycotina; Eurotiomycetes; Chaetothyriomycetidae; Verrucariales; Verrucariaceae; Endocarpon<br>Chromosome Unknown, exon count: 4 (XP_007802552) | cd08190<br>XP_007802552<br>(EPUS_01756)<br>492 aa |
| *Rasamsonia emersonii* CBS 393.64<br>Eukaryota; Fungi; Dikarya; Ascomycota; Pezizomycotina; Eurotiomycetes; Eurotiomycetidae; Eurotiales; Trichocomaceae; Rasamsonia<br>Chromosome Unknown, exon count: 3 (XP_013332422) | cd08190<br>XP_013332422<br>(T310_0196)<br>499 aa |
| *Talaromyces cellulolyticus*<br>Eukaryota; Fungi; Dikarya; Ascomycota; Pezizomycotina; Eurotiomycetes; Eurotiomycetidae; Eurotiales; Trichocomaceae; Talaromyces<br>Chromosome Unknown, exon count: 3 (GAM34277) | cd08190<br>GAM34277<br>(TCE0_015f01748)<br>497 aa |
| *Talaromyces islandicus*<br>Eukaryota; Fungi; Dikarya; Ascomycota; Pezizomycotina; Eurotiomycetes; Eurotiomycetidae; Eurotiales; Trichocomaceae; Talaromyces<br>Chromosome Unknown, exon count: 2 (CRG90176)<br>Chromosome Unknown, exon count: 3 (CRG84097) | CD08177<br>CRG90176<br>(PISL3812_07219)<br>357 aa<br><br>cd08190<br>CRG84097<br>(PISL3812_01431)<br>497 aa |
| *Talaromyces marneffei* ATCC 18224 (*Penicillium marneffei* ATCC 18224)<br>Eukaryota; Fungi; Dikarya; Ascomycota; Pezizomycotina; Eurotiomycetes; Eurotiomycetidae; Eurotiales; Trichocomaceae; Talaromyces<br>Chromosome Unknown, exon count: 3 (XP_002144749) | cd08190<br>XP_002144749<br>(PMAA_030510)<br>497 aa |
| *Talaromyces stipitatus* ATCC 10500<br>Eukaryota; Fungi; Dikarya; Ascomycota; Pezizomycotina; Eurotiomycetes; Eurotiomycetidae; Eurotiales; Trichocomaceae; Talaromyces<br>Chromosome Unknown, exon count: 2 (XP_002478063)<br>Chromosome Unknown, exon count: 3 (XP_002340727) | cd08177<br>XP_002478063<br>(TSTA_083320)<br>357 aa<br><br>cd08190<br>XP_002340727<br>(TSTA_067720)<br>497 aa |
| *Talaromyces verruculosus* TS63-9<br>Eukaryota; Fungi; Dikarya; Ascomycota; Pezizomycotina; Eurotiomycetes; Eurotiomycetidae; Eurotiales; Trichocomaceae; Talaromyces<br>Chromosome Unknown, exon count: 3 (KUL91107) | cd08190<br>KUL91107<br>(ZTR_00973)<br>497 aa |
| *Aspergillus ruber* CBS 135680<br>Eukaryota; Fungi; Dikarya; Ascomycota; Pezizomycotina; Eurotiomycetes; Eurotiomycetidae; Eurotiales; Aspergillaceae; Aspergillus<br>Chromosome Unknown, exon count: 2 (EYE99642)<br>Chromosome Unknown, exon count: 2 (EYE95237) | CD08177<br>EYE99642<br>(EURHEDRAFT_373566)<br>357 aa<br><br>cd08190<br>EYE95237<br>(EURHEDRAFT_479137)<br>491 aa |
| *Aspergillus clavatus* NRRL 1<br>Eukaryota; Fungi; Dikarya; Ascomycota; Pezizomycotina; Eurotiomycetes; Eurotiomycetidae; Eurotiales; Aspergillaceae; Aspergillus<br>Chromosome Unknown, exon count: 3 (XP_001272741) | cd08190<br>XP_001272741<br>(ACLA_090080)<br>494 aa |
| *Aspergillus flavus* AF70<br>Eukaryota; Fungi; Dikarya; Ascomycota; Pezizomycotina; Eurotiomycetes; Eurotiomycetidae; Eurotiales; Aspergillaceae; Aspergillus<br>Chromosome Unknown, exon count: 3 (KOC08334) | cd08190<br>KOC08334<br>(AFLA70_23g004720)<br>495 aa |
| *Aspergillus flavus* NRRL3357<br>Eukaryota; Fungi; Dikarya; Ascomycota; Pezizomycotina; Eurotiomycetes; Eurotiomycetidae; Eurotiales; Aspergillaceae; Aspergillus<br>Chromosome Unknown, exon count: 3 (XP_002375086) | cd08190<br>XP_002375086<br>(AFLA_035760)<br>495 aa |
| *Aspergillus lentulus*<br>Eukaryota; Fungi; Dikarya; Ascomycota; Pezizomycotina; Eurotiomycetes; Eurotiomycetidae; Eurotiales; Aspergillaceae; Aspergillus<br>Chromosome Unknown, exon count: 2 (GAQ03430)<br>Chromosome Unknown, exon count: 2 (GAQ07106) | cd08177<br>GAQ03430<br>(ALT_0751)<br>357 aa<br><br>cd08190<br>GAQ07106<br>(ALT_4427)<br>488 aa |

| Organism[1]<br>Lineage<br>Chromosome location and exon count | Fe-ADH family (conserved Domain Database)<br>Protein accesion number<br>(Gene locus)<br>Amino acid sequence lenght |
|---|---|
| *Aspergillus fischeri* NRRL 181 (*Neosartorya fischeri* NRRL 181)<br>Eukaryota; Fungi; Dikarya; Ascomycota; Pezizomycotina; Eurotiomycetes; Eurotiomycetidae; Eurotiales; Aspergillaceae; Aspergillus<br>Chromosome Unknown, exon count: 2 (XP_001257825)<br>Chromosome Unknown, exon count: 2 (XP_001260080) | cd08177<br>XP_001257825<br>(NFIA_052740)<br>357 aa<br><br>cd08190<br>XP_001260080<br>(NFIA_081270)<br>488 aa |
| *Aspergillus fumigatus* var. RP-2014<br>Eukaryota; Fungi; Dikarya; Ascomycota; Pezizomycotina; Eurotiomycetes; Eurotiomycetidae; Eurotiales; Aspergillaceae; Aspergillus<br>Chromosome Unknown, exon count: >2 (KEY77326-KEY84112) | cd08190<br>KEY77326-KEY84112<br>(BA78_8197 and BA78_8028)<br>324 aa (fragment) |
| *Aspergillus fumigatus* Af293<br>Eukaryota; Fungi; Dikarya; Ascomycota; Pezizomycotina; Eurotiomycetes; Eurotiomycetidae; Eurotiales; Aspergillaceae; Aspergillus<br>Chromosome 2, exon count: 2 (XP_749583) | cd08190<br>XP_749583<br>(AFUA_2G04520)<br>488 aa |
| *Aspergillus kawachii* IFO 4308<br>Eukaryota; Fungi; Dikarya; Ascomycota; Pezizomycotina; Eurotiomycetes; Eurotiomycetidae; Eurotiales; Aspergillaceae; Aspergillus<br>Chromosome Unknown, exon count: 3 (GAA83926) | cd08190<br>GAA83926<br>(AKAW_02041)<br>494 aa |
| *Aspergillus luchuensis*<br>Eukaryota; Fungi; Dikarya; Ascomycota; Pezizomycotina; Eurotiomycetes; Eurotiomycetidae; Eurotiales; Aspergillaceae; Aspergillus<br>Chromosome Unknown, exon count: 3 (GAT22293) | cd08190<br>GAT22293<br>(RIB2604_01503260)<br>508 aa |
| *Aspergillus nidulans* FGSC A4<br>Eukaryota; Fungi; Dikarya; Ascomycota; Pezizomycotina; Eurotiomycetes; Eurotiomycetidae; Eurotiales; Aspergillaceae; Aspergillus<br>Chromosome V, exon count: 2 (XP_662782)<br>Chromosome VII, exon count: 3 (XP_659472) | cd08177<br>XP_662782<br>(AN5178.2)<br>357 aa<br><br>cd08190<br>XP_659472<br>(AN1868.2)<br>494 aa |
| *Aspergillus niger* CBS 513.88<br>Eukaryota; Fungi; Dikarya; Ascomycota; Pezizomycotina; Eurotiomycetes; Eurotiomycetidae; Eurotiales; Trichocomaceae; mitosporic Trichocomaceae; Aspergillus<br>Chromosome Unknown, exon count: 2 (XP_001389108)<br>Chromosome Unknown, exon count: 1 (XP_001390430)<br>Chromosome Unknown, exon count: 3 (XP_001394313) | cd08177<br>XP_001389108<br>(ANI_1_2660014)<br>420 aa<br><br>cd08177<br>XP_001390430<br>(ANI_1_1442034)<br>383 aa<br><br>cd08190<br>XP_001394313<br>(ANI_1_430094)<br>494 aa |
| *Aspergillus niger* ATCC 1015<br>Eukaryota; Fungi; Dikarya; Ascomycota; Pezizomycotina; Eurotiomycetes; Eurotiomycetidae; Eurotiales; Aspergillaceae; Aspergillus<br>Chromosome Unknown, exon count: 3 (EHA19504) | cd08190<br>EHA19504<br>(ASPNIDRAFT_55964)<br>495 aa |
| *Aspergillus nomius* NRRL 13137<br>Eukaryota; Fungi; Dikarya; Ascomycota; Pezizomycotina; Eurotiomycetes; Eurotiomycetidae; Eurotiales; Aspergillaceae; Aspergillus<br>Chromosome Unknown, exon count: 3 (XP_015407952) | cd08190<br>XP_015407952<br>(ANOM_004685)<br>516 aa |
| *Aspergillus calidoustus*<br>Eukaryota; Fungi; Dikarya; Ascomycota; Pezizomycotina; Eurotiomycetes; Eurotiomycetidae; Eurotiales; Aspergillaceae; Aspergillus<br>Chromosome Unknown, exon count: 2 (CEN61391)<br>Chromosome Unknown, exon count: 3 (CEL05214) | cd08177<br>CEN61391<br>(ASPCAL08045)<br>357 aa<br><br>cd08190<br>CEL05214<br>(ASPCAL06333)<br>512 aa |
| *Aspergillus ochraceoroseus*<br>Eukaryota; Fungi; Dikarya; Ascomycota; Pezizomycotina; Eurotiomycetes; Eurotiomycetidae; Eurotiales; Aspergillaceae; Aspergillus<br>Chromosome Unknown, exon count: 4 (KKK15465) | cd08190<br>KKK15465<br>(AOCH_004544)<br>420 aa |
| *Aspergillus oryzae* RIB40<br>Eukaryota; Fungi; Dikarya; Ascomycota; Pezizomycotina; Eurotiomycetes; Eurotiomycetidae; Eurotiales; Aspergillaceae; Aspergillus<br>Chromosome Unknown, exon count: 3 (XP_001819453) | cd08190<br>XP_001819453<br>(AOR_1_406154)<br>495 aa |

| Organism[1]<br>Lineage<br>Chromosome location and exon count | Fe-ADH family (conserved Domain Database)<br>Protein accesion number<br>(Gene locus)<br>Amino acid sequence lenght |
|---|---|
| *Aspergillus oryzae* 3.042<br>Eukaryota; Fungi; Dikarya; Ascomycota; Pezizomycotina; Eurotiomycetes; Eurotiomycetidae; Eurotiales; Aspergillaceae; Aspergillus<br>Chromosome Unknown, exon count: 3 (EIT73331) | cd08190<br>EIT73331<br>(Ao3042_10714)<br>495 aa |
| *Aspergillus parasiticus* SU-1<br>Eukaryota; Fungi; Dikarya; Ascomycota; Pezizomycotina; Eurotiomycetes; Eurotiomycetidae; Eurotiales; Aspergillaceae; Aspergillus<br>Chromosome Unknown, exon count: 3 (KJK64599) | cd08190<br>KJK64599<br>(P875_00011109)<br>495 aa |
| *Aspergillus rambellii*<br>Eukaryota; Fungi; Dikarya; Ascomycota; Pezizomycotina; Eurotiomycetes; Eurotiomycetidae; Eurotiales; Aspergillaceae; Aspergillus<br>Chromosome Unknown, exon count: 3 (KKK12300) | cd08190<br>KKK12300<br>(ARAM_001384)<br>402 aa |
| *Aspergillus terreus* NIH2624<br>Eukaryota; Fungi; Dikarya; Ascomycota; Pezizomycotina; Eurotiomycetes; Eurotiomycetidae; Eurotiales; Aspergillaceae; Aspergillus<br>Chromosome Unknown, exon count: 3 (XP_001215236) | cd08190<br>XP_001215236<br>(ATEG_06058)<br>841 aa (FeADH domain located at residues 1-500 aa) |
| *Aspergillus udagawae* (*Neosartorya udagawae*)<br>Eukaryota; Fungi; Dikarya; Ascomycota; Pezizomycotina; Eurotiomycetes; Eurotiomycetidae; Eurotiales; Aspergillaceae; Aspergillus<br>Chromosome Unknown, exon count: 3 (GAO90864) | cd08190<br>GAO90864<br>(AUD_9824)<br>663 aa aa (FeADH domain located at residues 174-663 aa) |
| *Penicillium rubens Wisconsin* 54-1255 (*Penicillium chrysogenum Wisconsin* 54-1255)<br>Eukaryota; Fungi; Dikarya; Ascomycota; Pezizomycotina; Eurotiomycetes; Eurotiomycetidae; Eurotiales; Trichocomaceae; mitosporic Trichocomaceae; Penicillium; Penicillium chrysogenum complex<br>Chromosome Unknown, exon count: 2 (XP_002562381)<br>Chromosome Unknown, exon count: 3 (XP_ 002558613)<br>Chromosome Unknown, exon count: 3 (XP_002557879) | cd08177<br>XP_002562381<br>(Pc18g05540)<br>357 aa<br><br>cd08177<br>XP_ 002558613<br>CAP91237<br>(Pc13g01680)<br>408 aa<br><br>cd08190<br>XP_002557879<br>(Pc12g10580)<br>496 aa |
| *Penicillium brasilianum*<br>Eukaryota; Fungi; Dikarya; Ascomycota; Pezizomycotina; Eurotiomycetes; Eurotiomycetidae; Eurotiales; Aspergillaceae; Penicillium<br>Chromosome Unknown, exon count: 2 (CEO61086) | cd08190<br>CEO61086<br>(PMG11_05451)<br>495 aa |
| *Penicillium camemberti*<br>Eukaryota; Fungi; Dikarya; Ascomycota; Pezizomycotina; Eurotiomycetes; Eurotiomycetidae; Eurotiales; Aspergillaceae; Penicillium<br>Chromosome Unknown, exon count: 3 (CRL24280) | cd08190<br>CRL24280<br>(PCAMFM013_S011g000274)<br>496 aa |
| *Penicillium italicum*<br>Eukaryota; Fungi; Dikarya; Ascomycota; Pezizomycotina; Eurotiomycetes; Eurotiomycetidae; Eurotiales; Aspergillaceae; Penicillium<br>Chromosome Unknown, exon count: 2 (KGO77308)<br>Chromosome Unknown, exon count: 2 (KGO74750) | cd08177<br>KGO77308<br>(PITC_092680)<br>357 aa<br><br>cd08190<br>KGO74750<br>(PITC_083590)<br>515 aa |
| *Penicillium digitatum* Pd1<br>Eukaryota; Fungi; Dikarya; Ascomycota; Pezizomycotina; Eurotiomycetes; Eurotiomycetidae; Eurotiales; Aspergillaceae; Penicillium<br>Chromosome Unknown, exon count: 1 (XP_014531255)<br>Chromosome Unknown, exon count: 3 (XP_014532156) | cd08177<br>XP_014531255<br>(PDIP_70980)<br>378 aa<br><br>cd08190<br>XP_014532156<br>(PDIP_79990)<br>603 aa |
| *Penicillium expansum*<br>Eukaryota; Fungi; Dikarya; Ascomycota; Pezizomycotina; Eurotiomycetes; Eurotiomycetidae; Eurotiales; Aspergillaceae; Penicillium<br>Chromosome Unknown, exon count: 2 (KGO41250)<br>Chromosome Unknown, exon count: 5 (KGO57084) | cd08177<br>KGO41250<br>(PEXP_106300)<br>357 aa<br><br>cd08190<br>KGO57084<br>(PEX2_004380)<br>677 aa |

| Organism[1]<br>Lineage<br>Chromosome location and exon count | Fe-ADH family (conserved Domain Database)<br>Protein accesion number<br>(Gene locus)<br>Amino acid sequence lenght |
|---|---|
| *Penicillium freii*<br>Eukaryota; Fungi; Dikarya; Ascomycota; Pezizomycotina; Eurotiomycetes; Eurotiomycetidae; Eurotiales; Aspergillaceae; Penicillium<br>Chromosome Unknown, exon count: 3 (KUM62516) | cd08190<br>KUM62516<br>(ACN42_g4594)<br>496 aa |
| *Penicillium griseofulvum*<br>Eukaryota; Fungi; Dikarya; Ascomycota; Pezizomycotina; Eurotiomycetes; Eurotiomycetidae; Eurotiales; Trichocomaceae; mitosporic Trichocomaceae; Penicillium; Penicillium chrysogenum complex<br>Chromosome Unknown, exon count: 2 (KXG45472)<br>Chromosome Unknown, exon count: 3 (KXG48305) | cd08177<br>KXG45472<br>(PGRI_040210)<br>357 aa<br><br>cd08190<br>KXG48305<br>(PGRI_021750)<br>496 aa |
| *Penicillium nordicum*<br>Eukaryota; Fungi; Dikarya; Ascomycota; Pezizomycotina; Eurotiomycetes; Eurotiomycetidae; Eurotiales; Aspergillaceae; Penicillium<br>Chromosome Unknown, exon count: 3 (KOS46450) | cd08190<br>KOS46450<br>(ACN38_g2644)<br>496 aa |
| *Penicillium oxalicum* 114-2<br>Eukaryota; Fungi; Dikarya; Ascomycota; Pezizomycotina; Eurotiomycetes; Eurotiomycetidae; Eurotiales; Aspergillaceae; Penicillium<br>Chromosome Unknown, exon count: 2 (EPS34076) | cd08190<br>EPS34076<br>(PDE_09038)<br>495 aa |
| *Penicillium roqueforti* FM164<br>Eukaryota; Fungi; Dikarya; Ascomycota; Pezizomycotina; Eurotiomycetes; Eurotiomycetidae; Eurotiales; Aspergillaceae; Penicillium<br>Chromosome Unknown, exon count: 3 (CDM34552) | cd08190<br>CDM34552<br>(PROQFM164_S03g001276)<br>496 aa |
| *Penicillium solitum* RS1<br>Eukaryota; Fungi; Dikarya; Ascomycota; Pezizomycotina; Eurotiomycetes; Eurotiomycetidae; Eurotiales; Aspergillaceae; Penicillium<br>Chromosome Unknown, exon count: 3 (KJJ28324) | cd08190<br>KJJ28324<br>(U727_00430140261)<br>495 aa |
| *Byssochlamys spectabilis* No. 5<br>Eukaryota; Fungi; Dikarya; Ascomycota; Pezizomycotina; Eurotiomycetes; Eurotiomycetidae; Eurotiales; Thermoascaceae; Byssochlamys<br>Chromosome Unknown, exon count: 3 (GAD96117) | cd08190<br>GAD96117<br>(PVAR5_4766)<br>495 aa |
| *Coccidioides posadasii* str. *Silveira*<br>Eukaryota; Fungi; Dikarya; Ascomycota; Pezizomycotina; Eurotiomycetes; Eurotiomycetidae; Onygenales; Onygenales incertae sedis; Coccidioides<br>Chromosome Unknown, exon count: 3 (EFW15892) | CD08177<br>EFW15892<br>(CPSG_07519)<br>399 aa |
| *Paracoccidioides brasiliensis* Pb03<br>Eukaryota; Fungi; Dikarya; Ascomycota; Pezizomycotina; Eurotiomycetes; Eurotiomycetidae; Onygenales; mitosporic Onygenales; Paracoccidioides<br>Chromosome Unknown, exon count: 3 (EEH18707) | cd08190<br>EEH18707<br>(PABG_07767)<br>495 aa |
| *Paracoccidioides brasiliensis* Pb18<br>Eukaryota; Fungi; Dikarya; Ascomycota; Pezizomycotina; Eurotiomycetes; Eurotiomycetidae; Onygenales; mitosporic Onygenales; Paracoccidioides<br>Chromosome Unknown, exon count: 3 (XP_010763965) | cd08190<br>XP_010763965<br>EEH45018<br>(PADG_08649)<br>495 aa |
| *Paracoccidioides lutzii* Pb01<br>Eukaryota; Fungi; Dikarya; Ascomycota; Pezizomycotina; Eurotiomycetes; Eurotiomycetidae; Onygenales; mitosporic Onygenales; Paracoccidioides<br>Chromosome Unknown, exon count: 3 (XP_002789179) | cd08190<br>XP_002789179<br>(PAAG_08911)<br>495 aa |
| *Ajellomyces dermatitidis* SLH14081 (*Blastomyces gilchristii* SLH14081)<br>Eukaryota; Fungi; Dikarya; Ascomycota; Pezizomycotina; Eurotiomycetes; Eurotiomycetidae; Onygenales; Ajellomycetaceae; Ajellomyces<br>Chromosome Unknown, exon count: 3 (XP_002625467) | cd08190<br>XP_002625467<br>(BDBG_04336)<br>495 aa |
| *Emmonsia crescens* UAMH 3008<br>Eukaryota; Fungi; Dikarya; Ascomycota; Pezizomycotina; Eurotiomycetes; Eurotiomycetidae; Onygenales; Ajellomycetaceae; Emmonsia<br>Chromosome Unknown, exon count: 3 (KKZ60228) | cd08190<br>KKZ60228<br>(EMCG_05038)<br>495 aa |
| *Histoplasma capsulatum* NAm1 (Ajellomyces capsulatus NAm1)<br>Eukaryota; Fungi; Dikarya; Ascomycota; Pezizomycotina; Eurotiomycetes; Eurotiomycetidae; Onygenales; Ajellomycetaceae; Histoplasma<br>Chromosome Unknown, exon count: 3 (XP_001536070) | cd08190<br>XP_001536070<br>(HCAG_09021)<br>495 aa |
| *Histoplasma capsulatum* G186AR (Ajellomyces capsulatus G186AR)<br>Eukaryota; Fungi; Dikarya; Ascomycota; Pezizomycotina; Eurotiomycetes; Eurotiomycetidae; Onygenales; Ajellomycetaceae; Histoplasma<br>Chromosome Unknown, exon count: 3 (EEH09553) | cd08190<br>EEH09553<br>(HCBG_03090)<br>495 aa |
| *Histoplasma capsulatum* H88 (*Ajellomyces capsulatus* H88)<br>Eukaryota; Fungi; Dikarya; Ascomycota; Pezizomycotina; Eurotiomycetes; Eurotiomycetidae; Onygenales; Ajellomycetaceae; Histoplasma<br>Chromosome Unknown, exon count: 3 (EGC49241) | cd08190<br>EGC49241<br>(HCEG_08456)<br>495 aa |

| Organism[1]<br>Lineage<br>Chromosome location and exon count | Fe-ADH family (conserved Domain Database)<br>Protein accesion number<br>(Gene locus)<br>Amino acid sequence lenght |
|---|---|
| *Histoplasma capsulatum* H143 (*Ajellomyces capsulatus* H143)<br>Eukaryota; Fungi; Dikarya; Ascomycota; Pezizomycotina; Eurotiomycetes; Eurotiomycetidae; Onygenales; Ajellomycetaceae; Histoplasma<br>Chromosome Unknown, exon count: 3 (EER41381) | cd08190<br>EER41381<br>(HCDG_04028)<br>473 aa |
| *Emmonsia parva* UAMH 139<br>Eukaryota; Fungi; Dikarya; Ascomycota; Pezizomycotina; Eurotiomycetes; Eurotiomycetidae; Onygenales; Ajellomycetaceae; Emmonsia<br>Chromosome Unknown, exon count: 3 (KLJ07611) | cd08190<br>KLJ07611<br>(EMPG_10003)<br>495 aa |
| *Arthroderma benhamiae* CBS 112371<br>Eukaryota; Fungi; Dikarya; Ascomycota; Pezizomycotina; Eurotiomycetes; Eurotiomycetidae; Onygenales; Arthrodermataceae; Arthroderma<br>Chromosome Unknown, exon count: 3 (XP_003013778) | cd08190<br>XP_003013778<br>(ARB_07890)<br>500 aa |
| *Arthroderma gypseum* CBS 118893 (*Microsporum gypseum* CBS 118893)<br>Eukaryota; Fungi; Dikarya; Ascomycota; Pezizomycotina; Eurotiomycetes; Eurotiomycetidae; Onygenales; Arthrodermataceae; Arthroderma<br>Chromosome Unknown, exon count: 3 (XP_003169258) | cd08190<br>XP_003169258<br>(MGYG_08804)<br>498 aa |
| *Arthroderma otae* CBS 113480 (Microsporum canis CBS 113480)<br>Eukaryota; Fungi; Dikarya; Ascomycota; Pezizomycotina; Eurotiomycetes; Eurotiomycetidae; Onygenales; Arthrodermataceae; Arthroderma<br>Chromosome Unknown, exon count: 2 (XP_002849222)<br>Chromosome Unknown, exon count: 7 (EEQ30646)<br>Chromosome Unknown, exon count: 3 (XP_002842820) | CD08177<br>XP_002849222<br>(MCYG_02156)<br>357 aa<br><br>Cd08177<br>EEQ30646<br>XP_002847959<br>(MCYG_03465)<br>381 aa<br><br>cd08190<br>XP_002842820<br>(MCYG_08651)<br>497 aa |
| *Trichophyton equinum* CBS 127.97<br>Eukaryota; Fungi; Dikarya; Ascomycota; Saccharomyceta; Pezizomycotina; Leotiomyceta; Eurotiomycetes; Eurotiomycetidae; Onygenales; Arthrodermataceae; mitosporic Arthrodermataceae; Trichophyton<br>Chromosome Unknown, exon count: 3 (EGE05553) | cd08190<br>EGE05553<br>(TEQG_04562)<br>501 aa |
| *Trichophyton interdigitale* H6<br>Eukaryota; Fungi; Dikarya; Ascomycota; Saccharomyceta; Pezizomycotina; Leotiomyceta; Eurotiomycetes; Eurotiomycetidae; Onygenales; Arthrodermataceae; mitosporic Arthrodermataceae; Trichophyton<br>Chromosome Unknown, exon count: 3 (EZF35166) | cd08190<br>EZF35166<br>(H101_01311)<br>500 aa |
| *Trichophyton rubrum* CBS 118892<br>Eukaryota; Fungi; Dikarya; Ascomycota; Saccharomyceta; Pezizomycotina; Leotiomyceta; Eurotiomycetes; Eurotiomycetidae; Onygenales; Arthrodermataceae; mitosporic Arthrodermataceae; Trichophyton<br>Chromosome Unknown, exon count: 3 (XP_003235688) | cd08190<br>XP_003235688<br>(TERG_02744)<br>499 aa |
| *Trichophyton tonsurans* CBS 112818<br>Eukaryota; Fungi; Dikarya; Ascomycota; Saccharomyceta; Pezizomycotina; Leotiomyceta; Eurotiomycetes; Eurotiomycetidae; Onygenales; Arthrodermataceae; mitosporic Arthrodermataceae; Trichophyton<br>Chromosome Unknown, exon count: 3 (EGD98486) | cd08190<br>EGD98486<br>(TESG_05859)<br>501 aa |
| *Trichophyton verrucosum* HKI 0517<br>Eukaryota; Fungi; Dikarya; Ascomycota; Saccharomyceta; Pezizomycotina; Leotiomyceta; Eurotiomycetes; Eurotiomycetidae; Onygenales; Arthrodermataceae; mitosporic Arthrodermataceae; Trichophyton<br>Chromosome Unknown, exon count: 3 (XP_003024877) | cd08190<br>XP_003024877<br>(TRV_00952)<br>500 aa |
| *Dactylellina haptotyla* CBS 200.50<br>Eukaryota; Fungi; Dikarya; Ascomycota; Pezizomycotina; Orbiliomycetes; Orbiliales; Orbiliaceae; mitosporic Orbiliaceae; Dactylellina<br>Chromosome Unknown, exon count: 4 (XP_011115711) | cd08190<br>XP_011115711<br>(H072_10229)<br>495 aa |
| *Drechslerella stenobrocha* 248<br>Eukaryota; Fungi; Dikarya; Ascomycota; Pezizomycotina; Orbiliomycetes; Orbiliales; Orbiliaceae; mitosporic Orbiliaceae; Drechslerella<br>Chromosome Unknown, exon count: 5 (EWC46397) | cd08190<br>EWC46397<br>(DRE_04340)<br>496 aa |
| *Arthrobotrys oligospora* ATCC 24927<br>Eukaryota; Fungi; Dikarya; Ascomycota; Pezizomycotina; Orbiliomycetes; Orbiliales; Orbiliaceae; Orbilia<br>Chromosome Unknown, exon count: 4 (XP_011127448) | cd08190<br>XP_011127448<br>(AOL_s00210g345)<br>495 aa |
| *Tuber melanosporum* Mel28<br>Eukaryota; Fungi; Dikarya; Ascomycota; Pezizomycotina; Pezizomycetes; Pezizales; Tuberaceae; Tuber<br>Chromosome Unknown, exon count: 4 (XP_002838425) | cd08190<br>XP_002838425<br>(GSTUM_00001074001)<br>492 aa |

| Organism[1]<br>Lineage<br>Chromosome location and exon count | Fe-ADH family (conserved Domain Database)<br>Protein accesion number<br>(Gene locus)<br>Amino acid sequence lenght |
|---|---|
| *Uncinocarpus reesii* 1704<br>Eukaryota; Fungi; Dikarya; Ascomycota; Pezizomycotina; Eurotiomycetes; Eurotiomycetidae; Onygenales; Onygenaceae; Uncinocarpus<br>Chromosome Unknown, exon count: 3 (XP_002541656) | cd08190<br>XP_002541656<br>(UREG_01172)<br>496 aa |
| *Coccidioides immitis* RMSCC 2394<br>Eukaryota; Fungi; Dikarya; Ascomycota; Pezizomycotina; Eurotiomycetes; Eurotiomycetidae; Onygenales; Onygenales incertae sedis; Coccidioides<br>Chromosome Unknown, exon count: 4 (KMP01178) | cd08190<br>KMP01178<br>(CIRG_01318)<br>583 aa (FeADH domain located at residues 88-583 aa) |
| *Coccidioides immitis* RMSCC 3703<br>Eukaryota; Fungi; Dikarya; Ascomycota; Pezizomycotina; Eurotiomycetes; Eurotiomycetidae; Onygenales; Onygenales incertae sedis; Coccidioides<br>Chromosome Unknown, exon count: 3 (KMU74236) | cd08190<br>KMU74236<br>(CISG_04586)<br>496 aa |
| *Coccidioides immitis* RS<br>Eukaryota; Fungi; Dikarya; Ascomycota; Pezizomycotina; Eurotiomycetes; Eurotiomycetidae; Onygenales; Onygenales incertae sedis; Coccidioides<br>Chromosome Unknown, exon count: 3 (XP_001247471) | cd08190<br>XP_001247471<br>(CIMG_01242)<br>496 aa |
| *Colletotrichum fioriniae* PJ7<br>Eukaryota; Fungi; Dikarya; Ascomycota; Pezizomycotina; Sordariomycetes; Hypocreomycetidae; Glomerellales; Glomerellaceae; Colletotrichum<br>Chromosome Unknown, exon count: 3 (XP_007595740) | cd08190<br>XP_007595740<br>(CFIO01_06370)<br>498 aa |
| *Colletotrichum gloeosporioides* Cg-14<br>Eukaryota; Fungi; Dikarya; Ascomycota; Pezizomycotina; Sordariomycetes; Hypocreomycetidae; Glomerellales; Glomerellaceae; Colletotrichum<br>Chromosome Unknown, exon count: 3 (EQB56649) | cd08190<br>EQB56649<br>(CGLO_03324)<br>496 aa |
| *Colletotrichum graminicola* M1.001<br>Eukaryota; Fungi; Dikarya; Ascomycota; Pezizomycotina; Sordariomycetes; Hypocreomycetidae; Glomerellales; Glomerellaceae; Colletotrichum<br>Chromosome Unknown, exon count: 3 (XP_008094325) | cd08190<br>XP_008094325<br>(GLRG_05449)<br>496 aa |
| *Colletotrichum higginsianum* IMI 34906<br>Eukaryota; Fungi; Dikarya; Ascomycota; Pezizomycotina; Sordariomycetes; Hypocreomycetidae; Glomerellales; Glomerellaceae; Colletotrichum<br>Chromosome Unknown, exon count: 3 (CCF36507) | cd08190<br>CCF36507<br>(CH063_00226)<br>499 aa |
| *Colletotrichum nymphaeae* SA-01<br>Eukaryota; Fungi; Dikarya; Ascomycota; Pezizomycotina; Sordariomycetes; Hypocreomycetidae; Glomerellales; Glomerellaceae; Colletotrichum<br>Chromosome Unknown, exon count: 3 (KXH44801) | cd08190<br>KXH44801<br>(CNYM01_10111)<br>498 aa |
| *Colletotrichum orbiculare* MAFF 240422<br>Eukaryota; Fungi; Dikarya; Ascomycota; Pezizomycotina; Sordariomycetes; Hypocreomycetidae; Glomerellales; Glomerellaceae; Colletotrichum<br>Chromosome Unknown, exon count: 3 (ENH80057) | cd08190<br>ENH80057<br>(Cob_01119)<br>498 aa |
| *Colletotrichum salicis* CBS 607.94 (*Glomerella miyabeana*)<br>Eukaryota; Fungi; Dikarya; Ascomycota; Pezizomycotina; Sordariomycetes; Hypocreomycetidae; Glomerellales; Glomerellaceae; Colletotrichum<br>Chromosome Unknown, exon count: 3 (KXH67768) | cd08190<br>KXH67768<br>(CSAL01_12668)<br>482 aa |
| *Colletotrichum simmondsii* CBS122122<br>Eukaryota; Fungi; Dikarya; Ascomycota; Pezizomycotina; Sordariomycetes; Hypocreomycetidae; Glomerellales; Glomerellaceae; Colletotrichum<br>Chromosome Unknown, exon count: 3 (KXH25791) | cd08190<br>KXH25791<br>(CSIM01_07222)<br>498 aa |
| *Colletotrichum sublineola*<br>Eukaryota; Fungi; Dikarya; Ascomycota; Pezizomycotina; Sordariomycetes; Hypocreomycetidae; Glomerellales; Glomerellaceae; Colletotrichum<br>Chromosome Unknown, exon count: 3 (KDN61515) | cd08190<br>KDN61515<br>(CSUB01_00598)<br>499 aa |
| *Verticillium alfalfae* VaMs.102 (*Verticillium albo-atrum* VaMs.102)<br>Eukaryota; Fungi; Dikarya; Ascomycota; Pezizomycotina; Sordariomycetes; Hypocreomycetidae; Glomerellales; Plectosphaerellaceae; mitosporic Plectosphaerellaceae; Verticillium<br>Chromosome Unknown, exon count: 3 (XP_003001667) | cd08190<br>XP_003001667<br>(VDBG_07926)<br>448 aa |
| *Verticillium dahliae* VdLs.17<br>Eukaryota; Fungi; Dikarya; Ascomycota; Pezizomycotina; Sordariomycetes; Hypocreomycetidae; Glomerellales; Plectosphaerellaceae; mitosporic Plectosphaerellaceae; Verticillium<br>Chromosome 2, exon count: 2 (XP_009650688) | cd08190<br>XP_009650688<br>(VDAG_05498)<br>498 aa |
| *Verticillium longisporum* VL1<br>Eukaryota; Fungi; Dikarya; Ascomycota; Pezizomycotina; Sordariomycetes; Hypocreomycetidae; Glomerellales; Plectosphaerellaceae; mitosporic Plectosphaerellaceae; Verticillium<br>Chromosome Unknown, exon count: 2 (CRK12066) | cd08190<br>CRK12066<br>(BN1708_010322)<br>498 aa |
| *Claviceps purpurea* 20.1<br>Eukaryota; Fungi; Dikarya; Ascomycota; Pezizomycotina; Sordariomycetes; Hypocreomycetidae; Hypocreales; Clavicipitaceae; Claviceps<br>Chromosome Unknown, exon count: 4 (CCE30352) | cd08190<br>CCE30352<br>(CPUR_04200<br>497 aa |

| Organism[1]<br>Lineage<br>Chromosome location and exon count | Fe-ADH family (conserved Domain Database)<br>Protein accesion number<br>(Gene locus)<br>Amino acid sequence lenght |
|---|---|
| *Torrubiella hemipterigena* (anamorph: Verticillium hemipterigenum)<br>Eukaryota; Fungi; Dikarya; Ascomycota; Pezizomycotina; Sordariomycetes; Hypocreomycetidae; Hypocreales; Clavicipitaceae; Torrubiella<br>Chromosome Unknown, exon count: 1 (CEJ83026)<br>Chromosome Unknown, exon count: 4 (CEJ94220) | CD08177<br>CEJ83026<br>(VHEMI03059)<br>360 aa<br><br>cd08190<br>CEJ94220<br>(VHEMI09763)<br>495 aa |
| *Beauveria bassiana* ARSEF 2860<br>Eukaryota; Fungi; Dikarya; Ascomycota; Pezizomycotina; Sordariomycetes; Hypocreomycetidae; Hypocreales; Cordycipitaceae; mitosporic Cordycipitaceae; Beauveria<br>Chromosome Unknown, exon count: 4 (XP_008596701) | cd08190<br>XP_008596701<br>(BBA_03382)<br>499 aa |
| *Beauveria bassiana* D1-5<br>Eukaryota; Fungi; Dikarya; Ascomycota; Pezizomycotina; Sordariomycetes; Hypocreomycetidae; Hypocreales; Cordycipitaceae; mitosporic Cordycipitaceae; Beauveria<br>Chromosome Unknown, exon count: 4 (KGQ09708) | cd08190<br>KGQ09708<br>(BBAD15_g4943)<br>502 aa |
| *Cordyceps militaris* CM01<br>Eukaryota; Fungi; Dikarya; Ascomycota; Pezizomycotina; Sordariomycetes; Hypocreomycetidae; Hypocreales; Cordycipitaceae; Cordyceps<br>Chromosome Unknown, exon count: 4 (XP_006665579) | cd08190<br>XP_006665579<br>(CCM_00356)<br>497 aa |
| *Escovopsis weberi*<br>Eukaryota; Fungi; Dikarya; Ascomycota; Pezizomycotina; Sordariomycetes; Hypocreomycetidae; Hypocreales; Hypocreaceae; Escovopsis<br>Chromosome Unknown, exon count: >4 (KOS22693) | cd08190<br>KOS22693<br>(ESCO_003740)<br>218 aa (fragment) |
| *Trichoderma atroviride* IMI 206040<br>Eukaryota; Fungi; Dikarya; Ascomycota; Pezizomycotina; Sordariomycetes; Hypocreomycetidae; Hypocreales; Hypocreaceae; Hypocrea; mitosporic Hypocrea; Trichoderma<br>Chromosome Unknown, exon count: 2 (XP_013949133)<br>Chromosome Unknown, exon count: 5 (XP_013939012) | CD08177<br>XP_013949133<br>(TRIATDRAFT_54661)<br>357 aa<br><br>cd08190<br>XP_013939012<br>EHK40535<br>(TRIATDRAFT_301384)<br>493 aa |
| *Trichoderma gamsii*<br>Eukaryota; Fungi; Dikarya; Ascomycota; Pezizomycotina; Sordariomycetes; Hypocreomycetidae; Hypocreales; Hypocreaceae; Hypocrea; mitosporic Hypocrea; Trichoderma<br>Chromosome Unknown, exon count: 2 (KUE94885)<br>Chromosome Unknown, exon count: 4 (KUE98387) | CD08177<br>KUE94885<br>(TGAM01_10627)<br>357 aa<br><br>cd08190<br>KUE98387<br>(TGAM01_07191)<br>493 aa |
| *Trichoderma harzianum* (Hypocrea lixii)<br>Eukaryota; Fungi; Dikarya; Ascomycota; Pezizomycotina; Sordariomycetes; Hypocreomycetidae; Hypocreales; Hypocreaceae; Hypocrea; mitosporic Hypocrea; Trichoderma<br>Chromosome Unknown, exon count: 2 (KKP04410)<br>Chromosome Unknown, exon count: 4 (KKP01192) | CD08177<br>KKP04410<br>(THAR02_03486)<br>361 aa<br><br>cd08190<br>KKP01192<br>(THAR02_06718)<br>494 aa |
| *Trichoderma reesei* QM6a<br>Eukaryota; Fungi; Dikarya; Ascomycota; Pezizomycotina; Sordariomycetes; Hypocreomycetidae; Hypocreales; Hypocreaceae; Hypocrea; mitosporic Hypocrea; Trichoderma<br>Chromosome Unknown, exon count: 2 (XP_006960945)<br>Chromosome Unknown, exon count: 4 (XP_006962805) | CD08177<br>XP_006960945<br>(TRIREDRAFT_2038)<br>357 aa<br><br>cd08190<br>XP_006962805<br>(TRIREDRAFT_104606)<br>495 aa |
| *Trichoderma reesei* RUT C-30<br>Eukaryota; Fungi; Dikarya; Ascomycota; Pezizomycotina; Sordariomycetes; Hypocreomycetidae; Hypocreales; Hypocreaceae; Hypocrea; mitosporic Hypocrea; Trichoderma<br>Chromosome Unknown, exon count: 4 (ETS04301) | cd08190<br>ETS04301<br>(M419DRAFT_33138)<br>499 aa |
| *Trichoderma virens* Gv29-8<br>Eukaryota; Fungi; Dikarya; Ascomycota; Pezizomycotina; Sordariomycetes; Hypocreomycetidae; Hypocreales; Hypocreaceae; Hypocrea; mitosporic Hypocrea; Trichoderma<br>Chromosome Unknown, exon count: 4 (XP_013955690) | cd08190<br>XP_013955690<br>(TRIVIDRAFT_78522)<br>494 aa |

| Organism[1]<br>Lineage<br>Chromosome location and exon count | Fe-ADH family (conserved Domain Database)<br>Protein accesion number<br>(Gene locus)<br>Amino acid sequence lenght |
|---|---|
| *Fusarium avenaceum* (*Gibberella avenacea*)<br>Eukaryota; Fungi; Dikarya; Ascomycota; Pezizomycotina; Sordariomycetes; Hypocreomycetidae; Hypocreales; Nectriaceae; Fusarium; Fusarium tricinctum species complex<br>Chromosome Unknown, exon count: 4 (KIL93347) | cd08190<br>KIL93347<br>(FAVG1_03327)<br>495 |
| *Fusarium langsethiae*<br>Eukaryota; Fungi; Dikarya; Ascomycota; Pezizomycotina; Sordariomycetes; Hypocreomycetidae; Hypocreales; Nectriaceae; Fusarium<br>Chromosome Unknown, exon count: 4 (KPA44512) | cd08190<br>KPA44512<br>(FLAG1_02562)<br>495 aa |
| *Fusarium verticillioides* M-3125<br>Eukaryota; Fungi; Dikarya; Ascomycota; Pezizomycotina; Sordariomycetes; Hypocreomycetidae; Hypocreales; Nectriaceae; Fusarium; Fusarium fujikuroi species complex.<br>Chromosome Unknown, exon count: 1 (AAG27129) | cd08177<br>AAG27129<br>(FUM7)<br>424 aa (Characterized protein) |
| *Fusarium verticillioides* 7600<br>Eukaryota; Fungi; Dikarya; Ascomycota; Pezizomycotina; Sordariomycetes; Hypocreomycetidae; Hypocreales; Nectriaceae; Fusarium; Fusarium fujikuroi species complex.<br>Chromosome Unknown, exon count: 1 (EWG36197)<br>Chromosome Unknown, exon count: 4 (EWG41865) | cd08177<br>EWG36197<br>(FVEG_00319)<br>424 aa (identical to AAG27129)<br><br>cd08190<br>EWG41865<br>(FVEG_03863)<br>495 aa |
| *Fusarium oxysporum* O-1890<br>Eukaryota; Fungi; Dikarya; Ascomycota; Pezizomycotina; Sordariomycetes; Hypocreomycetidae; Hypocreales; Nectriaceae; Fusarium; Fusarium oxysporum species complex<br>Chromosome Unknown, exon count: 1 (ACB12554) | cd08177<br>ACB12554<br>(Fum7)<br>419 aa (characterized protein) |
| *Fusarium oxysporum* Fo5176<br>Eukaryota; Fungi; Dikarya; Ascomycota; Pezizomycotina; Sordariomycetes; Hypocreomycetidae; Hypocreales; Nectriaceae; Fusarium; Fusarium oxysporum species complex<br>Chromosome Unknown, exon count: 4 (EGU81371) | cd08190<br>EGU81371<br>(FOXB_08100)<br>495 aa |
| *Fusarium oxysporum* f. sp. *vasinfectum* 25433<br>Eukaryota; Fungi; Dikarya; Ascomycota; Pezizomycotina; Sordariomycetes; Hypocreomycetidae; Hypocreales; Nectriaceae; Fusarium; Fusarium oxysporum species complex<br>Chromosome Unknown, exon count: 4 (EXM23065) | cd08190<br>EXM23065<br>(FOTG_09394)<br>495 aa |
| *Fusarium oxysporum* f. sp. *cubense tropical race* 4 54006<br>Eukaryota; Fungi; Dikarya; Ascomycota; Pezizomycotina; Sordariomycetes; Hypocreomycetidae; Hypocreales; Nectriaceae; Fusarium; Fusarium oxysporum species complex<br>Chromosome Unknown, exon count: 4 (EXM03274) | cd08190<br>EXM03274<br>(FOIG_06127)<br>495 aa |
| *Fusarium oxysporum* f. sp. *cubense race* 1<br>Eukaryota; Fungi; Dikarya; Ascomycota; Pezizomycotina; Sordariomycetes; Hypocreomycetidae; Hypocreales; Nectriaceae; Fusarium; Fusarium oxysporum species complex<br>Chromosome Unknown, exon count: 4 (ENH69661) | cd08190<br>ENH69661<br>(FOC1_g10012018)<br>495 aa |
| *Fusarium fujikuroi* IMI 58289<br>Eukaryota; Fungi; Dikarya; Ascomycota; Pezizomycotina; Sordariomycetes; Hypocreomycetidae; Hypocreales; Nectriaceae; Fusarium; Fusarium fujikuroi species complex<br>Chromosome Unknown, exon count: 4 (CCT64597) | cd08190<br>CCT64597<br>(FFUJ_04166)<br>495 aa |
| *Fusarium graminearum* PH-1 (anamorph: *Fusarium graminearum*)<br>Eukaryota; Fungi; Dikarya; Ascomycota; Pezizomycotina; Sordariomycetes; Hypocreomycetidae; Hypocreales; Nectriaceae; Fusarium<br>Chromosome Unknown, exon count: Unknown | cd08177<br>XP_384039<br>(FG03863.1)<br>893 aa (FeADH domain located at residues 470-893 aa) |
| *Fusarium pseudograminearum* CS3096<br>Eukaryota; Fungi; Dikarya; Ascomycota; Pezizomycotina; Sordariomycetes; Hypocreomycetidae; Hypocreales; Nectriaceae; Fusarium<br>Chromosome Unknown, exon count: 1 (EKJ73192)<br>Chromosome Unknown, exon count: 4 (XP_009261027) | cd08177<br>EKJ73192<br>XP_009258009<br>(FPSE_06616)<br>413 aa<br><br>cd08190<br>XP_009261027<br>EKJ70109<br>(FPSE_09635)<br>496 aa |
| *Nectria haematococca* mpVI 77-13-4<br>Eukaryota; Fungi; Dikarya; Ascomycota; Pezizomycotina; Sordariomycetes; Hypocreomycetidae; Hypocreales; Nectriaceae; Nectria; Nectria haematococca complex<br>Chromosome Unknown, exon count: 4 (XP_003046821) | cd08190<br>XP_003046821<br>(NECHADRAFT_58609)<br>495 aa |

| Organism[1]<br>Lineage<br>Chromosome location and exon count | Fe-ADH family (conserved Domain Database)<br>Protein accesion number<br>(Gene locus)<br>Amino acid sequence lenght |
|---|---|
| *Neonectria ditissima*<br>Eukaryota; Fungi; Dikarya; Ascomycota; Pezizomycotina; Sordariomycetes; Hypocreomycetidae; Hypocreales; Nectriaceae; Neonectria<br>Chromosome Unknown, exon count: 4 (KPM34751) | cd08190<br>KPM34751<br>(AK830_g11830)<br>495 aa |
| *Metarhizium acridum* CQMa 102<br>Eukaryota; Fungi; Dikarya; Ascomycota; Pezizomycotina; Sordariomycetes; Hypocreomycetidae; Hypocreales; Clavicipitaceae; Metarhizium.<br>Chromosome Unknown, exon count: 4 (XP_007809734) | cd08190<br>XP_007809734<br>(MAC_03394)<br>497 aa |
| *Metarhizium album* ARSEF 1941<br>Eukaryota; Fungi; Dikarya; Ascomycota; Pezizomycotina; Sordariomycetes; Hypocreomycetidae; Hypocreales; Clavicipitaceae; Metarhizium.<br>Chromosome Unknown, exon count: 4 (KHN99027) | cd08190<br>KHN99027<br>(MAM_02725)<br>497 aa |
| *Metarhizium anisopliae*<br>Eukaryota; Fungi; Dikarya; Ascomycota; Pezizomycotina; Sordariomycetes; Hypocreomycetidae; Hypocreales; Clavicipitaceae; Metarhizium.<br>Chromosome Unknown, exon count: 4 (KFG85816) | cd08190<br>KFG85816<br>(MANI_007637)<br>497 aa |
| *Metarhizium anisopliae* BRIP 53284<br>Eukaryota; Fungi; Dikarya; Ascomycota; Pezizomycotina; Sordariomycetes; Hypocreomycetidae; Hypocreales; Clavicipitaceae; Metarhizium.<br>Chromosome Unknown, exon count: 4 (KJK94263) | cd08190<br>KJK94263<br>(H633G_01841)<br>497 aa |
| *Metarhizium anisopliae* BRIP 53293<br>Eukaryota; Fungi; Dikarya; Ascomycota; Pezizomycotina; Sordariomycetes; Hypocreomycetidae; Hypocreales; Clavicipitaceae; Metarhizium.<br>Chromosome Unknown, exon count: 4 (KJK81755) | cd08190<br>KJK81755<br>(H634G_03018)<br>497 aa |
| *Metarhizium brunneum* ARSEF 3297<br>Eukaryota; Fungi; Dikarya; Ascomycota; Pezizomycotina; Sordariomycetes; Hypocreomycetidae; Hypocreales; Clavicipitaceae; Metarhizium.<br>Chromosome Unknown, exon count: 4 (XP_014547023) | cd08190<br>XP_014547023<br>(MBR_02316)<br>497 aa |
| *Metarhizium guizhouense* ARSEF 977<br>Eukaryota; Fungi; Dikarya; Ascomycota; Pezizomycotina; Sordariomycetes; Hypocreomycetidae; Hypocreales; Clavicipitaceae; Metarhizium.<br>Chromosome Unknown, exon count: 1 (KID84173)<br>Chromosome Unknown, exon count: 4 (KID93133) | CD08177<br>KID84173<br>(MGU_08587)<br>363 aa<br><br>cd08190<br>KID93133<br>(MGU_00722)<br>497 aa |
| *Metarhizium majus* ARSEF 297<br>Eukaryota; Fungi; Dikarya; Ascomycota; Pezizomycotina; Sordariomycetes; Hypocreomycetidae; Hypocreales; Clavicipitaceae; Metarhizium.<br>Chromosome Unknown, exon count: 4 (XP_014582811) | cd08190<br>XP_014582811<br>(MAJ_00349)<br>497 aa |
| *Metarhizium robertsii* ARSEF 23<br>Eukaryota; Fungi; Dikarya; Ascomycota; Pezizomycotina; Sordariomycetes; Hypocreomycetidae; Hypocreales; Clavicipitaceae; Metarhizium.<br>Chromosome Unknown, exon count: 4 (XP_007818183) | cd08190<br>XP_007818183<br>(MAA_01994)<br>497 aa |
| *Drechmeria coniospora*<br>Eukaryota; Fungi; Dikarya; Ascomycota; Pezizomycotina; Sordariomycetes; Hypocreomycetidae; Hypocreales; Ophiocordycipitaceae; Drechmeria<br>Chromosome Unknown, exon count: 4 (KYK60641) | cd08190<br>KYK60641<br>(DCS_01779)<br>495 aa |
| *Hirsutella minnesotensis* 3608<br>Eukaryota; Fungi; Dikarya; Ascomycota; Pezizomycotina; Sordariomycetes; Hypocreomycetidae; Hypocreales; Ophiocordycipitaceae; Hirsutella<br>Chromosome Unknown, exon count: 4 (KJZ74577) | cd08190<br>KJZ74577<br>(HIM_05927)<br>496 aa |
| *Ophiocordyceps unilateralis*<br>Eukaryota; Fungi; Dikarya; Ascomycota; Pezizomycotina; Sordariomycetes; Hypocreomycetidae; Hypocreales; Ophiocordycipitaceae; Ophiocordyceps<br>Chromosome Unknown, exon count: 4 (KOM23703) | cd08190<br>KOM23703<br>(XA68_2923)<br>489 aa |
| *Ophiocordyceps sinensis* CO18<br>Eukaryota; Fungi; Dikarya; Ascomycota; Pezizomycotina; Sordariomycetes; Hypocreomycetidae; Hypocreales; Ophiocordycipitaceae; Ophiocordyceps<br>Chromosome Unknown, exon count: 3 (EQK99626) | cd08190<br>EQK99626<br>(OCS_04655)<br>424 aa |
| *Tolypocladium ophioglossoides* CBS 100239 (*Elaphocordyceps ophioglossoides* CBS 100239)<br>Eukaryota; Fungi; Dikarya; Ascomycota; Pezizomycotina; Sordariomycetes; Hypocreomycetidae; Hypocreales; Ophiocordycipitaceae; Tolypocladium<br>Chromosome Unknown, exon count: 4 (KND88605) | cd08190<br>KND88605<br>(TOPH_06769)<br>497 aa |
| *Stachybotrys chartarum* IBT 40293<br>Eukaryota; Fungi; Dikarya; Ascomycota; Pezizomycotina; Sordariomycetes; Hypocreomycetidae; Hypocreales; Stachybotriaceae; Stachybotrys<br>Chromosome Unknown, exon count: 4 (KFA50928) | cd08190<br>KFA50928<br>(S40293_02465)<br>497 aa |

| Organism[1]<br>Lineage<br>Chromosome location and exon count | Fe-ADH family (conserved Domain Database)<br>Protein accesion number<br>(Gene locus)<br>Amino acid sequence lenght |
|---|---|
| *Stachybotrys chartarum* IBT 7711<br>Eukaryota; Fungi; Dikarya; Ascomycota; Pezizomycotina; Sordariomycetes; Hypocreomycetidae; Hypocreales; Stachybotriaceae; Stachybotrys<br>Chromosome Unknown, exon count: 4 (KEY65886) | cd08190<br>KEY65886<br>(S7711_09393)<br>497 aa |
| *Stachybotrys chlorohalonata* IBT 40285<br>Eukaryota; Fungi; Dikarya; Ascomycota; Pezizomycotina; Sordariomycetes; Hypocreomycetidae; Hypocreales; Stachybotriaceae; Stachybotrys<br>Chromosome Unknown, exon count: 4 (KFA60499) | cd08190<br>KFA60499<br>(S40285_06910)<br>497 aa |
| *Acremonium chrysogenum* ATCC 11550<br>Eukaryota; Fungi; Dikarya; Ascomycota; Pezizomycotina; Sordariomycetes; Hypocreomycetidae; Hypocreales; Hypocreales incertae sedis; Acremonium<br>Chromosome Unknown, exon count: 4 (KFH46069) | cd08190<br>KFH46069<br>(ACRE_031150)<br>501 aa |
| *Ustilaginoidea virens* (*Villosiclava virens*)<br>Eukaryota; Fungi; Dikarya; Ascomycota; Pezizomycotina; Sordariomycetes; Hypocreomycetidae; Hypocreales; Hypocreales incertae sedis; Ustilaginoideae; Ustilaginoidea<br>Chromosome Unknown, exon count: 4 (KDB14331) | cd08190<br>KDB14331<br>(UV8b_4926)<br>497 aa |
| *Ceratocystis platani*<br>Eukaryota; Fungi; Dikarya; Ascomycota; Pezizomycotina; Sordariomycetes; Hypocreomycetidae; Microascales; Ceratocystidaceae; Ceratocystis<br>Chromosome Unknown, exon count: 3 (KKF97464) | cd08190<br>KKF97464<br>(CFO_g211)<br>275 aa (fragment) |
| *Thielaviopsis punctulata*<br>Eukaryota; Fungi; Dikarya; Ascomycota; Pezizomycotina; Sordariomycetes; Hypocreomycetidae; Microascales; Ceratocystidaceae; Thielaviopsis<br>Chromosome Unknown, exon count: 3 (KKA30276) | cd08190<br>KKA30276<br>(TD95_004388)<br>468 aa |
| *Scedosporium apiospermum* (*Pseudallescheria apiosperma*)<br>Eukaryota; Fungi; Dikarya; Ascomycota; Pezizomycotina; Sordariomycetes; Hypocreomycetidae; Microascales; Microascaceae; Scedosporium<br>Chromosome Unknown, exon count: 4 (KEZ39498) | cd08190<br>KEZ39498<br>(SAPIO_CDS9364)<br>498 aa |
| *Valsa mali* var. Pyri<br>Eukaryota; Fungi; Dikarya; Ascomycota; Pezizomycotina; Sordariomycetes; Sordariomycetidae; Diaporthales; Valsaceae; Valsa.<br>Chromosome Unknown, exon count: 2 (KUI59709)<br>Chromosome Unknown, exon count: 3 (KUI56111) | CD08177<br>KUI59709<br>(VP1G_06939)<br>367 aa<br><br>cd08190<br>KUI56111<br>(VP1G_03489)<br>493 aa |
| *Valsa mali*<br>Eukaryota; Fungi; Dikarya; Ascomycota; Pezizomycotina; Sordariomycetes; Sordariomycetidae; Diaporthales; Valsaceae; Valsa.<br>Chromosome Unknown, exon count: 2 (KUI64218)<br>Chromosome Unknown, exon count: 3 (KUI73354) | CD08177<br>KUI64218<br>(VM1G_10998)<br>343 aa<br><br>cd08190<br>KUI73354<br>(VM1G_09053)<br>493 aa |
| *Togninia minima* UCRPA7<br>Eukaryota; Fungi; Dikarya; Ascomycota; Pezizomycotina; Sordariomycetes; Sordariomycetidae; Calosphaeriales; Calosphaeriaceae; Togninia<br>Chromosome Unknown, exon count: 1 (EOO01596)<br>Chromosome Unknown, exon count: 4 (XP_007918024) | CD08178<br>EOO01596<br>XP_007913653<br>(UCRPA7_2908)<br>912 aa (bidomain)<br><br>cd08190<br>XP_007918024<br>(UCRPA7_7301)<br>417 aa |
| *Diaporthe ampelina*<br>Eukaryota; Fungi; Dikarya; Ascomycota; Pezizomycotina; Sordariomycetes; Sordariomycetidae; Diaporthales; Diaporthaceae; Diaporthe<br>Chromosome Unknown, exon count: 4 (KKY35621) | cd08190<br>KKY35621<br>(UCDDA912_g04402)<br>443 aa |
| *Gaeumannomyces graminis var. tritici R3-111a-1*<br>Eukaryota; Fungi; Dikarya; Ascomycota; Pezizomycotina; Sordariomycetes; Sordariomycetidae; Magnaporthales; Magnaporthaceae; Gaeumannomyces<br>Chromosome Unknown, exon count: 2 (XP_009223503) | cd08190<br>XP_009223503<br>(GGTG_07415)<br>497 aa |
| *Magnaporthe oryzae* 70-15<br>Eukaryota; Fungi; Dikarya; Ascomycota; Pezizomycotina; Sordariomycetes; Sordariomycetidae; Magnaporthales; Magnaporthaceae; Magnaporthe.<br>Chromosome 6, exon count: 3 (XP_003720069) | cd08190<br>XP_003720069<br>(MGG_03824)<br>494 aa |

| Organism[1]<br>Lineage<br>Chromosome location and exon count | Fe-ADH family (conserved Domain Database)<br>Protein accesion number<br>(Gene locus)<br>Amino acid sequence lenght |
|---|---|
| *Magnaporthe oryzae* P131<br>Eukaryota; Fungi; Dikarya; Ascomycota; Pezizomycotina; Sordariomycetes; Sordariomycetidae; Magnaporthales; Magnaporthaceae; Magnaporthe.<br>Chromosome Unknown, exon count: 3 (ELQ58584) | cd08190<br>ELQ58584<br>(OOW_P131scaffold01576g2)<br>488 aa |
| *Magnaporthe oryzae* Y34<br>Eukaryota; Fungi; Dikarya; Ascomycota; Pezizomycotina; Sordariomycetes; Sordariomycetidae; Magnaporthales; Magnaporthaceae; Magnaporthe.<br>Chromosome Unknown, exon count: 6 (ELQ33498)<br>Chromosome Unknown, exon count: 3 (ELQ32716) | cd08177<br>ELQ33498<br>(OOU_Y34scaffold00936g25)<br>385 aa (corrected)<br><br>cd08190<br>ELQ32716<br>(OOU_Y34scaffold01069g4)<br>488 aa |
| *Magnaporthiopsis poae ATCC 64411 (Magnaporthe poae ATCC 64411)*<br>Eukaryota; Fungi; Dikarya; Ascomycota; Pezizomycotina; Sordariomycetes; Sordariomycetidae; Magnaporthales; Magnaporthaceae; Magnaporthiopsis<br>Chromosome Unknown, exon count: 1 (KLU87939) | cd08190<br>KLU87939<br>(MAPG_06929)<br>499 aa |
| *Grosmannia clavigera* kw1407<br>Eukaryota; Fungi; Dikarya; Ascomycota; Pezizomycotina; Sordariomycetes; Sordariomycetidae; Ophiostomatales; Ophiostomataceae; Grosmannia<br>Chromosome Unknown, exon count: 3 (XP_014173917) | cd08190<br>XP_014173917<br>(CMQ_1363)<br>495 aa |
| *Ophiostoma piceae* UAMH 11346<br>Eukaryota; Fungi; Dikarya; Ascomycota; Pezizomycotina; Sordariomycetes; Sordariomycetidae; Ophiostomatales; Ophiostomataceae; Ophiostoma<br>Chromosome Unknown, exon count: 2 (EPE02416) | cd08190<br>EPE02416<br>(F503_00684)<br>495 aa |
| *Sporothrix brasiliensis* 5110<br>Eukaryota; Fungi; Dikarya; Ascomycota; Pezizomycotina; Sordariomycetes; Sordariomycetidae; Ophiostomatales; Ophiostomataceae; Sporothrix<br>Chromosome Unknown, exon count: 3 (KIH88796) | cd08190<br>KIH88796<br>(SPBR_07018)<br>499 aa |
| *Sporothrix schenckii* ATCC 58251<br>Eukaryota; Fungi; Dikarya; Ascomycota; Pezizomycotina; Sordariomycetes; Sordariomycetidae; Ophiostomatales; Ophiostomataceae; Sporothrix<br>Chromosome Unknown, exon count: 3 (ERT02475) | cd08190<br>ERT02475<br>(HMPREF1624_00774)<br>499 aa |
| *Chaetomium globosum* CBS 148.51<br>Eukaryota; Fungi; Dikarya; Ascomycota; Pezizomycotina; Sordariomycetes; Sordariomycetidae; Sordariales; Chaetomiaceae; Chaetomium<br>Chromosome Unknown, exon count: 4 (XP_001227183) | cd08190<br>XP_001227183<br>(CHGG_09256)<br>514 aa |
| *Chaetomium thermophilum* var. *thermophilum* DSM 1495<br>Eukaryota; Fungi; Dikarya; Ascomycota; Pezizomycotina; Sordariomycetes; Sordariomycetidae; Sordariales; Chaetomiaceae; Chaetomium<br>Chromosome Unknown, exon count: 4 (XP_006694734) | cd08190<br>XP_006694734<br>(CHGG_09256)<br>1530 aa (FeADH domain at residues 1030-1530) |
| *Myceliophthora thermophila ATCC 42464*<br>Eukaryota; Fungi; Dikarya; Ascomycota; Pezizomycotina; Sordariomycetes; Sordariomycetidae; Sordariales; Chaetomiaceae; mitosporic Chaetomiaceae; Myceliophthora<br>Chromosome 4, exon count: 3 (XP_003664003) | cd08190<br>XP_003664003<br>(MYCTH_2306298)<br>511 aa |
| *Thielavia terrestris* NRRL 8126<br>Eukaryota; Fungi; Dikarya; Ascomycota; Pezizomycotina; Sordariomycetes; Sordariomycetidae; Sordariales; Chaetomiaceae; Thielavia<br>Chromosome 1, exon count: 4 (XP_003650327) | cd08190<br>XP_003650327<br>(THITE_2063410)<br>512 aa |
| *Podospora anserina S mat+*<br>Eukaryota; Fungi; Dikarya; Ascomycota; Pezizomycotina; Sordariomycetes; Sordariomycetidae; Sordariales; Lasiosphaeriaceae; Podospora<br>Chromosome 4, exon count: 4 (XP_001906205) | cd08190<br>XP_001906205<br>(PODANSg3233)<br>507 aa |
| *Madurella mycetomatis* mm55<br>Eukaryota; Fungi; Dikarya; Ascomycota; Pezizomycotina; Sordariomycetes; Sordariomycetidae; Sordariales; mitosporic Sordariales; Madurella<br>Chromosome Unknown, exon count: 4 (KXX83351) | cd08190<br>KXX83351<br>(MMYC01_200084)<br>511 aa |
| *Neurospora crassa* OR74A<br>Eukaryota; Fungi; Dikarya; Ascomycota; Pezizomycotina; Sordariomycetes; Sordariomycetidae; Sordariales; Sordariaceae; Neurospora<br>Chromosome: Linkage group VI, exon count: 3 (XP_957693) | cd08190<br>XP_957693<br>(NCU04078)<br>501 aa |
| *Neurospora tetrasperma* FGSC 2508<br>Eukaryota; Fungi; Dikarya; Ascomycota; Pezizomycotina; Sordariomycetes; Sordariomycetidae; Sordariales; Sordariaceae; Neurospora<br>Chromosome Unknown, exon count: 3 (XP_009854371) | cd08190<br>XP_009854371<br>(NEUTE1DRAFT_87713)<br>501 aa |
| *Sordaria macrospora k-hell*<br>Eukaryota; Fungi; Dikarya; Ascomycota; Pezizomycotina; Sordariomycetes; Sordariomycetidae; Sordariales; Sordariaceae; Sordaria<br>Chromosome Unknown, exon count: 3 (XP_003349774) | cd08190<br>XP_003349774<br>(SMAC_00662)<br>507 AA |

| Organism[1]<br>Lineage<br>Chromosome location and exon count | Fe-ADH family (conserved Domain Database)<br>Protein accesion number<br>(Gene locus)<br>Amino acid sequence lenght |
|---|---|
| *Eutypa lata* UCREL1<br>Eukaryota; Fungi; Dikarya; Ascomycota; Pezizomycotina; Sordariomycetes; Xylariomycetidae; Xylariales; Diatrypaceae; Eutypa<br>Chromosome Unknown, exon count: 5 (XP_007791329) | cd08190<br>XP_007791329<br>(UCREL1_3396)<br>451 aa |
| *Microdochium bolleyi*<br>Eukaryota; Fungi; Dikarya; Ascomycota; Pezizomycotina; Sordariomycetes; Xylariomycetidae; Xylariales; Microdochiaceae; Microdochium<br>Chromosome Unknown, exon count: 3 (KXJ97333) | cd08190<br>KXJ97333<br>(Micbo1qcDRAFT_142937)<br>501 aa |
| *Pestalotiopsis fici* W106-1<br>Eukaryota; Fungi; Dikarya; Ascomycota; Pezizomycotina; Sordariomycetes; Xylariomycetidae; Xylariales; Sporocadaceae; Pestalotiopsis<br>Chromosome Unknown, exon count: 3 (XP_007836589) | cd08190<br>XP_007836589<br>(PFICI_09817)<br>499 aa |
| *Rosellinia necatrix*<br>Eukaryota; Fungi; Dikarya; Ascomycota; Pezizomycotina; Sordariomycetes; Xylariomycetidae; Xylariales; Xylariaceae; Rosellinia<br>Chromosome Unknown, exon count: 4 (GAP88418) | cd08190<br>GAP88418<br>(SAMD00023353_3000770)<br>502 aa |
| *Blumeria graminis* f. sp. *hordei* DH14<br>Eukaryota; Fungi; Dikarya; Ascomycota; Pezizomycotina; Leotiomycetes; Erysiphales; Erysiphaceae; Blumeria<br>Chromosome Unknown, exon count: 4 (CCU82647) | cd08190<br>CCU82647<br>(BGHDH14_bghG006725000001001)<br>497 aa |
| *Erysiphe necator* (*Uncinula necator*)<br>Eukaryota; Fungi; Dikarya; Ascomycota; Pezizomycotina; Leotiomycetes; Erysiphales; Erysiphaceae; Erysiphe<br>Chromosome Unknown, exon count: 4 (KHJ35541) | cd08190<br>KHJ35541<br>(EV44_g6522)<br>505 aa |
| *Marssonina brunnea* f. sp. 'multigermtubi' MB_m1<br>Eukaryota; Fungi; Dikarya; Ascomycota; Pezizomycotina; Leotiomycetes; Helotiales; Dermateaceae; Marssonina<br>Chromosome Unknown, exon count: 5 (XP_007294265) | cd08190<br>XP_007294265<br>(MBM_06376)<br>500 aa |
| *Glarea lozoyensis* ATCC 20868<br>Eukaryota; Fungi; Dikarya; Ascomycota; Pezizomycotina; Leotiomycetes; Helotiales; Helotiaceae; Glarea<br>Chromosome Unknown, exon count: 4 (XP_008078189) | cd08190<br>XP_008078189<br>(GLAREA_10898)<br>496 aa |
| *Botrytis cinerea* BcDW1<br>Eukaryota; Fungi; Dikarya; Ascomycota; Pezizomycotina; Leotiomycetes; Helotiales; Sclerotiniaceae; Botrytis<br>Chromosome Unknown, exon count: 4 (EMR83588) | cd08190<br>EMR83588<br>(BcDW1_7778)<br>497 aa |
| *Botrytis cinerea* T4<br>Eukaryota; Fungi; Dikarya; Ascomycota; Pezizomycotina; Leotiomycetes; Helotiales; Sclerotiniaceae; Botrytis<br>Chromosome Unknown, exon count: 4 (CCD48190) | cd08190<br>CCD48190<br>(BofuT4P27000001001)<br>497 aa |
| *Sclerotinia borealis* F-4157<br>Eukaryota; Fungi; Dikarya; Ascomycota; Pezizomycotina; Leotiomycetes; Helotiales; Sclerotiniaceae; Sclerotinia<br>Chromosome Unknown, exon count: 4 (ESZ95094) | cd08190<br>ESZ95094<br>(SBOR_4549)<br>498 aa |
| *Sclerotinia sclerotiorum* 1980<br>Eukaryota; Fungi; Dikarya; Ascomycota; Pezizomycotina; Leotiomycetes; Helotiales; Sclerotiniaceae; Sclerotinia<br>Chromosome Unknown, exon count: 4 (XP_001585607) | cd08190<br>XP_001585607<br>(SS1G_13491)<br>498 aa |
| *Phialocephala scopiformis*<br>Eukaryota; Fungi; Dikarya; Ascomycota; Pezizomycotina; Leotiomycetes; Helotiales; Helotiales incertae sedis; Phialocephala<br>Chromosome Unknown, exon count: 5 (KUJ23064) | cd08190<br>KUJ23064<br>(LY89DRAFT_713602)<br>499 aa |
| *Oidiodendron maius* Zn<br>Eukaryota; Fungi; Dikarya; Ascomycota; Pezizomycotina; Leotiomycetes; Leotiomycetes incertae sedis; Myxotrichaceae; mitosporic Myxotrichaceae; Oidiodendron<br>Chromosome Unknown, exon count: 4 (KIN01293) | cd08190<br>KIN01293<br>(OIDMADRAFT_198681)<br>499 aa |
| *Pseudogymnoascus destructans* 20631-21<br>Eukaryota; Fungi; Dikarya; Ascomycota; Pezizomycotina; Leotiomycetes; Leotiomycetes incertae sedis; Pseudeurotiaceae; Pseudogymnoascus<br>Chromosome Unknown, exon count: 4 (XP_012740407) | cd08190<br>XP_012740407<br>ELR06130<br>(GMDG_02004)<br>499 aa |

| Organism[1]<br>Lineage<br>Chromosome location and exon count | Fe-ADH family (conserved Domain Database)<br>Protein accesion number<br>(Gene locus)<br>Amino acid sequence lenght |
|---|---|
| *Pseudogymnoascus* sp. VKM F-103<br>Eukaryota; Fungi; Dikarya; Ascomycota; Pezizomycotina; Leotiomycetes; Leotiomycetes incertae sedis; Pseudeurotiaceae; Pseudogymnoascus.<br>Chromosome Unknown, exon count: 2 (KFY73232)<br>Chromosome Unknown, exon count: 1 (KFY80066)<br>Chromosome Unknown, exon count: 1 (KFY79477)<br>Chromosome Unknown, exon count: 4 (KFY77194) | CD08177<br>KFY73232<br>(V499_06672)<br>360 aa<br><br>CD08177<br>KFY80066<br>(V499_01041)<br>360 aa<br><br>CD08177<br>KFY79477<br>(V499_01537)<br>360 aa<br><br>cd08190<br>KFY77194<br>(V499_03357)<br>472 aa (corrected) |
| *Pseudogymnoascus* sp. VKM F-3775<br>Eukaryota; Fungi; Dikarya; Ascomycota; Pezizomycotina; Leotiomycetes; Leotiomycetes incertae sedis; Pseudeurotiaceae; Pseudogymnoascus.<br>Chromosome Unknown, exon count: 2 (KFY28602) | CD08177<br>KFY28602<br>(V491_00387)<br>346 aa |
| *Pseudogymnoascus* sp. VKM F-3808<br>Eukaryota; Fungi; Dikarya; Ascomycota; Pezizomycotina; Leotiomycetes; Leotiomycetes incertae sedis; Pseudeurotiaceae; Pseudogymnoascus.<br>Chromosome Unknown, exon count: 2 (KFX93692) | CD08177<br>KFX93692<br>(O988_06660)<br>360 aa |
| *Pseudogymnoascus* sp. VKM F-4246<br>Eukaryota; Fungi; Dikarya; Ascomycota; Pezizomycotina; Leotiomycetes; Leotiomycetes incertae sedis; Pseudeurotiaceae; Pseudogymnoascus.<br>Chromosome Unknown, exon count: 2 (KFY12350) | CD08177<br>KFY12350<br>(V492_03931)<br>352 aa |
| *Pseudogymnoascus* sp. VKM F-4513 (FW-928)<br>Eukaryota; Fungi; Dikarya; Ascomycota; Pezizomycotina; Leotiomycetes; Leotiomycetes incertae sedis; Pseudeurotiaceae; Pseudogymnoascus.<br>Chromosome Unknown, exon count: 1 (KFY40669)<br>Chromosome Unknown, exon count: 3 (KFY45970) | CD08177<br>KFY40669<br>(V494_03388)<br>368 aa<br><br>cd08190<br>KFY45970<br>(V494_00690)<br>499 aa |
| *Pseudogymnoascus* sp. VKM F-4515 (FW-2607)<br>Eukaryota; Fungi; Dikarya; Ascomycota; Pezizomycotina; Leotiomycetes; Leotiomycetes incertae sedis; Pseudeurotiaceae; Pseudogymnoascus.<br>Chromosome Unknown, exon count: 13 (KFY52687)<br>Chromosome Unknown, exon count: 4 (KFY55257) | CD08177<br>KFY52687<br>(V496_08273)<br>1179 aa (FeADH domain located at residues 851-1179aa)<br><br>cd08190<br>KFY55257<br>(V496_07031)<br>499 aa |
| *Pseudogymnoascus* sp. VKM F-4516 (FW-969)<br>Eukaryota; Fungi; Dikarya; Ascomycota; Pezizomycotina; Leotiomycetes; Leotiomycetes incertae sedis; Pseudeurotiaceae; Pseudogymnoascus.<br>Chromosome Unknown, exon count: 7 (KFY62180) | cd08190<br>KFY62180<br>(V497_02519)<br>1428 aa (FeADH domain located at residues 930-1428aa) |
| *Pseudogymnoascus* sp. VKM F-4517 (FW-2822)<br>Eukaryota; Fungi; Dikarya; Ascomycota; Pezizomycotina; Leotiomycetes; Leotiomycetes incertae sedis; Pseudeurotiaceae; Pseudogymnoascus.<br>Chromosome Unknown, exon count: 18 (KFY87842) | CD08177<br>KFY87842<br>(V498_07005)<br>2200 aa (FeADH domain located at residues 1496-1850aa) |
| *Pseudogymnoascus* sp. VKM F-4518 (FW-2643)<br>Eukaryota; Fungi; Dikarya; Ascomycota; Pezizomycotina; Leotiomycetes; Leotiomycetes incertae sedis; Pseudeurotiaceae; Pseudogymnoascus.<br>Chromosome Unknown, exon count: 2 (KFY97662)<br>Chromosome Unknown, exon count: 1 (KFY86601)<br>Chromosome Unknown, exon count: 4 (KFY84162) | CD08177<br>KFY97662<br>(V500_01969)<br>357 aa<br><br>CD08177<br>KFY86601<br>(V500_07532)<br>360 aa<br><br>cd08190<br>KFY84162<br>(V500_09546)<br>452 aa |

| Organism[1]<br>Lineage<br>Chromosome location and exon count | Fe-ADH family (conserved Domain Database)<br>Protein accesion number<br>(Gene locus)<br>Amino acid sequence lenght |
|---|---|
| *Pseudogymnoascus* sp. VKM F-4519 (FW-2642)<br>Eukaryota; Fungi; Dikarya; Ascomycota; Pezizomycotina; Leotiomycetes; Leotiomycetes incertae sedis; Pseudeurotiaceae; Pseudogymnoascus.<br>Chromosome Unknown, exon count: 1 (KFZ08521)<br>Chromosome Unknown, exon count: 2 (KFZ06809)<br>Chromosome Unknown, exon count: 4 (KFZ13608) | CD08177<br>KFZ08521<br>(V501_05924)<br>360 aa<br><br>CD08177<br>KFZ06809<br>(V501_07059)<br>360 aa<br><br>cd08190<br>KFZ13608<br>(V501_03624)<br>499 aa |
| *Pseudogymnoascus* sp. VKM F-4520 (FW-2644)<br>Eukaryota; Fungi; Dikarya; Ascomycota; Pezizomycotina; Leotiomycetes; Leotiomycetes incertae sedis; Pseudeurotiaceae; Pseudogymnoascus.<br>Chromosome Unknown, exon count: 1 (KFZ24260)<br>Chromosome Unknown, exon count: 4 (KFZ23376) | CD08177<br>KFZ24260<br>(V502_01252)<br>360 aa<br><br>cd08190<br>KFZ23376<br>(V502_02152)<br>499 aa |
| *Dothistroma septosporum* NZE10<br>Eukaryota; Fungi; Dikarya; Ascomycota; Pezizomycotina; Dothideomycetes; Dothideomycetidae; Capnodiales; Mycosphaerellaceae; Dothistroma<br>Chromosome Unknown, exon count: 3 (EME50327) | cd08190<br>EME50327<br>(DOTSEDRAFT_69004)<br>494 aa |
| *Mycosphaerella eumusae* (anamorph: *Pseudocercospora eumusae*)<br>Eukaryota; Fungi; Dikarya; Ascomycota; Pezizomycotina; Dothideomycetes; Dothideomycetidae; Capnodiales; Mycosphaerellaceae; Mycosphaerella.<br>Chromosome Unknown, exon count: 2 (KXT03593)<br>Chromosome Unknown, exon count: 1 (KXT04691) | CD08177<br>KXT03593<br>(AC578_9981)<br>359 aa<br><br>cd08190<br>KXT04691<br>(AC578_2112)<br>493 aa |
| *Pseudocercospora fijiensis* CIRAD86 (*Mycosphaerella fijiensis* CIRAD86)<br>Eukaryota; Fungi; Dikarya; Ascomycota; Pezizomycotina; Dothideomycetes; Dothideomycetidae; Capnodiales; Mycosphaerellaceae; Pseudocercospora.<br>Chromosome Unknown, exon count: 2 (EME88408)<br>Chromosome Unknown, exon count: 4 (EME79549)<br>Chromosome Unknown, exon count: 1 (XP_007920184) | CD08177<br>EME88408<br>XP_007919695<br>(MYCFIDRAFT_25384)<br>360 aa<br><br>CD08177<br>EME79549<br>(MYCFIDRAFT_61341)<br>389 aa<br><br>cd08190<br>XP_007920184<br>EME89377<br>(MYCFIDRAFT_55798)<br>493 aa |
| *Sphaerulina musiva* SO2202 (*Mycosphaerella populorum* SO2202, *Septoria musiva* SO2202)<br>Eukaryota; Fungi; Dikarya; Ascomycota; Pezizomycotina; Dothideomycetes; Dothideomycetidae; Capnodiales; Mycosphaerellaceae; Sphaerulina<br>Chromosome Unknown, exon count: 3 (EMF17798) | cd08190<br>EMF17798<br>XP_016765919<br>(SEPMUDRAFT_105001)<br>533 aa |
| *Zymoseptoria brevis*<br>Eukaryota; Fungi; Dikarya; Ascomycota; Pezizomycotina; Dothideomycetes; Dothideomycetidae; Capnodiales; Mycosphaerellaceae; Zymoseptoria<br>Chromosome Unknown, exon count: 2 (KJX96650)<br>Chromosome Unknown, exon count: 3 (KJX95343) | CD08177<br>KJX96650<br>(TI39_contig609g00012)<br>360 aa<br><br>cd08190<br>KJX95343<br>(TI39_contig4119g00038)<br>495 aa |

| Organism[1]<br>Lineage<br>Chromosome location and exon count | Fe-ADH family (conserved Domain Database)<br>Protein accesion number<br>(Gene locus)<br>Amino acid sequence lenght |
|---|---|
| *Zymoseptoria tritici* IPO323 (*Mycosphaerella graminicola* IPO323)<br>Eukaryota; Fungi; Dikarya; Ascomycota; Pezizomycotina; Dothideomycetes; Dothideomycetidae; Capnodiales; Mycosphaerellaceae; Zymoseptoria<br>Chromosome 5, exon count: 2 (XP_003852066)<br>Chromosome 1, exon count: 3 (XP_003856468) | CD08177<br>XP_003852066<br>(MYCGRDRAFT_71772)<br>360 aa<br><br>cd08190<br>XP_003856468<br>(MYCGRDRAFT_66658)<br>495 aa |
| *Baudoinia panamericana* UAMH 10762 (*Baudoinia compniacensis* UAMH 10762)<br>Eukaryota; Fungi; Dikarya; Ascomycota; Pezizomycotina; Dothideomycetes; Dothideomycetidae; Capnodiales; Teratosphaeriaceae; Baudoinia<br>Chromosome 1, exon count: 3 (XP_007678306)<br>Chromosome 1, exon count: 3 (XP_007673313) | CD08177<br>XP_007678306<br>BAUCODRAFT_548167<br>360 aa<br><br>cd08190<br>XP_007673313<br>(BAUCODRAFT_348772)<br>494 aa |
| *Aureobasidium melanogenum* CBS 110374<br>Eukaryota; Fungi; Dikarya; Ascomycota; Pezizomycotina; Dothideomycetes; Dothideomycetidae; Dothideales; Aureobasidiaceae; Aureobasidium<br>Chromosome Unknown, exon count: 1 (KEQ61369)<br>Chromosome Unknown, exon count: 2 (KEQ66480) | CD08177<br>KEQ61369<br>(M437DRAFT_85819)<br>360 aa<br><br>cd08190<br>KEQ66480<br>(M437DRAFT_72050)<br>490 aa |
| *Aureobasidium namibiae* CBS 147.97<br>Eukaryota; Fungi; Dikarya; Ascomycota; Pezizomycotina; Dothideomycetes; Dothideomycetidae; Dothideales; Aureobasidiaceae; Aureobasidium<br>Chromosome Unknown, exon count: 1 (XP_013428431)<br>Chromosome Unknown, exon count: 2 (XP_013426753) | CD08177<br>XP_013428431<br>(M436DRAFT_43616)<br>360 aa<br><br>cd08190<br>XP_013426753<br>(M436DRAFT_73282)<br>490 aa |
| *Aureobasidium pullulans* EXF-150<br>Eukaryota; Fungi; Dikarya; Ascomycota; Pezizomycotina; Dothideomycetes; Dothideomycetidae; Dothideales; Aureobasidiaceae; Aureobasidium<br>Chromosome Unknown, exon count: 2 (KEQ89192) | cd08190<br>KEQ89192<br>(M438DRAFT_340998)<br>490 aa |
| *Aureobasidium subglaciale* EXF-2481 (*Aureobasidium pullulans* var. *subglaciale* EXF-2481)<br>Eukaryota; Fungi; Dikarya; Ascomycota; Pezizomycotina; Dothideomycetes; Dothideomycetidae; Dothideales; Aureobasidiaceae; Aureobasidium<br>Chromosome Unknown, exon count: 1 (XP_013342698)<br>Chromosome Unknown, exon count: 2 (XP_013343871) | CD08177<br>XP_013342698<br>(AUEXF2481DRAFT_292294)<br>362 aa<br><br>cd08190<br>XP_013343871<br>(AUEXF2481DRAFT_40008)<br>490 aa |
| *Acidomyces richmondensis*<br>Eukaryota; Fungi; Dikarya; Ascomycota; Pezizomycotina; Dothideomycetes; Dothideomycetes incertae sedis; Acidomyces<br>Chromosome Unknown, exon count: 2 (KXL43521)<br>Chromosome Unknown, exon count: 1 (KXL46316) | CD08177<br>KXL43521<br>(FE78DRAFT_72480)<br>360 aa<br><br>cd08190<br>KXL46316<br>(FE78DRAFT_146422)<br>493 aa |
| *Neofusicoccum parvum* UCRNP2<br>Eukaryota; Fungi; Dikarya; Ascomycota; Pezizomycotina; Dothideomycetes; Dothideomycetes incertae sedis; Botryosphaeriales; Botryosphaeriaceae; Neofusicoccum<br>Chromosome Unknown, exon count: 3 (XP_007585355) | cd08190<br>XP_007585355<br>(UCRNP2_6086)<br>503 aa |
| *Verruconis gallopava* (*Ochroconis gallopava* CBS43764)<br>Eukaryota; Fungi; Dikarya; Ascomycota; Pezizomycotina; Dothideomycetes; Dothideomycetes incertae sedis; Venturiales; Sympoventuriaceae; Verruconis<br>Chromosome Unknown, exon count: 2 (KIW04238) | cd08190<br>KIW04238<br>(PV09_04544)<br>500 aa |
| *Geotrichum candidum* CLIB 918<br>Eukaryota; Fungi; Dikarya; Ascomycota; Saccharomycotina; Saccharomycetes; Saccharomycetales; Dipodascaceae; Geotrichum<br>Chromosome Unknown, exon count: 1 (CDO55355) | cd08190<br>CDO55355<br>(BN980_GECA10s03893g)<br>496 aa |

| Organism[1]<br>Lineage<br>Chromosome location and exon count | Fe-ADH family (conserved Domain Database)<br>Protein accesion number<br>(Gene locus)<br>Amino acid sequence lenght |
|---|---|
| *Yarrowia lipolytica* CLIB122<br>Eukaryota; Fungi; Dikarya; Ascomycota; Saccharomycotina; Saccharomycetes; Saccharomycetales; Dipodascaceae; Yarrowia<br>Chromosome E, exon count: 1 (XP_503997) | cd08190<br>XP_503997<br>(YALI0E15818g)<br>492 aa |
| *Lachancea quebecensis*<br>Eukaryota; Fungi; Dikarya; Ascomycota; Saccharomycotina; Saccharomycetes; Saccharomycetales; Saccharomycetaceae; Lachancea<br>Chromosome Unknown, exon count: 1 | CD08176<br>CUS23793<br>(LAQU0_S12e00144g)<br>382 aa |
| *Kluyveromyces lactis* NRRL Y-1140<br>Eukaryota; Fungi; Dikarya; Ascomycota; Saccharomycotina; Saccharomycetes; Saccharomycetales; Saccharomycetaceae; Kluyveromyces<br>Chromosome C, exon count: 1 | CD08176<br>XP_453065<br>(KLLA0C19382g)<br>418 aa |
| *Saccharomyces cerevisiae* S288c<br>Eukaryota; Fungi; Dikarya; Ascomycota; Saccharomycotina; Saccharomycetes; Saccharomycetales; Saccharomycetaceae; Saccharomyces<br>Also known as NRC465; ZRG5<br>Chromosome VII, exon count: 1 | CD08176<br>P10127<br>NP_011258<br>(ADH4)<br>382 aa |
| *Saccharomyces cerevisiae* RM11-1a<br>Eukaryota; Fungi; Dikarya; Ascomycota; Saccharomycotina; Saccharomycetes; Saccharomycetales; Saccharomycetaceae; Saccharomyces<br>Chromosome Unknown, exon count: 1 | CD08176<br>EDV10456<br>(SCRG_01240)<br>465 aa |
| *Saccharomyces kudriavzevii* IFO 1802<br>Eukaryota; Fungi; Dikarya; Ascomycota; Saccharomycotina; Saccharomycetes; Saccharomycetales; Saccharomycetaceae; Saccharomyces<br>Chromosome Unknown, exon count: 1 | CD08176<br>EJT43785<br>(YGL256W; SKUD_204908)<br>427 aa |
| *Torulaspora delbrueckii*<br>Eukaryota; Fungi; Dikarya; Ascomycota; Saccharomycotina; Saccharomycetes; Saccharomycetales; Saccharomycetaceae; Torulaspora<br>Chromosome 7, exon count: 1 | CD08176<br>XP_003683057<br>(TDEL0G04790)<br>419 aa |
| *Schizosaccharomyces cryophilus* OY26<br>Eukaryota; Fungi; Dikarya; Ascomycota; Taphrinomycotina; Schizosaccharomycetes; Schizosaccharomycetales; Schizosaccharomycetaceae; Schizosaccharomyces<br>Chromosome Unknown, exon count: 1 | CD08176<br>XP_013022584<br>EPY52707<br>(SPOG_02026)<br>446 aa |
| *Schizosaccharomyces octosporus* yFS286<br>Eukaryota; Fungi; Dikarya; Ascomycota; Taphrinomycotina; Schizosaccharomycetes; Schizosaccharomycetales; Schizosaccharomycetaceae; Schizosaccharomyces<br>Chromosome Unknown, exon count: 1 | CD08176<br>XP_013018352<br>EPX72716<br>(SOCG_00478)<br>443 aa |
| *Schizosaccharomyces japonicus* yFS275<br>Eukaryota; Fungi; Dikarya; Ascomycota; Taphrinomycotina; Schizosaccharomycetes; Schizosaccharomycetales; Schizosaccharomycetaceae; Schizosaccharomyces<br>Chromosome Unknown, exon count: 1 (XP_002171533)<br>Chromosome Unknown, exon count: 1 (XP_002174952) | CD08176<br>XP_002171533<br>(SJAG_00240)<br>378 aa<br><br>CD08176<br>XP_002174952<br>(SJAG_03822)<br>377 aa |
| *Schizosaccharomyces pombe* 972h-<br>Eukaryota; Fungi; Dikarya; Ascomycota; Taphrinomycotina; Schizosaccharomycetes; Schizosaccharomycetales; Schizosaccharomycetaceae; Schizosaccharomyces<br>Chromosome 1, exon count: 1 (NP_592819) | CD08176<br>NP_592819<br>Q09669 (ADH4_SCHPO)<br>(adh4; SPAC5H10.06c)<br>422 aa |
| *Taphrina deformans* PYCC 5710<br>Eukaryota; Fungi; Dikarya; Ascomycota; Taphrinomycotina; Taphrinomycetes; Taphrinales; Taphrinaceae; Taphrina<br>Chromosome Unknown, exon count: 1 (CCG81770) | cd08190<br>CCG81770<br>(TAPDE_001617)<br>448 aa |
| *Saitoella complicata* NRRL Y-17804<br>Eukaryota; Fungi; Dikarya; Ascomycota; Taphrinomycotina; Taphrinomycotina incertae sedis; Saitoella<br>Chromosome Unknown, exon count: 1 (GAO47549) | cd08190<br>GAO47549<br>(G7K_1754-t1)<br>487 aa |
| Basidiomycota | |
| *Agaricus bisporus* var. *burnettii* JB137-S8<br>Eukaryota; Fungi; Dikarya; Basidiomycota; Agaricomycotina; Agaricomycetes; Agaricomycetidae; Agaricales; Agaricaceae; Agaricus<br>Chromosome Unknown, exon count: 13 (XP_007331711)<br>Chromosome Unknown, exon count: 13 (XP_007329474) | cd08190<br>XP_007331711<br>(AGABI1DRAFT_61606)<br>494 aa<br><br>cd08190<br>XP_007329474<br>(AGABI1DRAFT_113486)<br>493aa |

| Organism[1]<br>Lineage<br>Chromosome location and exon count | Fe-ADH family (conserved Domain Database)<br>Protein accesion number<br>(Gene locus)<br>Amino acid sequence lenght |
|---|---|
| *Agaricus bisporus* var. *bisporus* H97<br>Eukaryota; Fungi; Dikarya; Basidiomycota; Agaricomycotina; Agaricomycetes; Agaricomycetidae; Agaricales; Agaricaceae; Agaricus<br>Chromosome Unknown, exon count: 13 (XP_006457439)<br>Chromosome Unknown, exon count: 13 (XP_006460096) | cd08190<br>XP_006457439<br>(AGABI2DRAFT_229861)<br>494 aa<br><br>cd08190<br>XP_006460096<br>(AGABI2DRAFT_191865)<br>494 aa |
| *Leucoagaricus* sp. SymC.cos<br>Eukaryota; Fungi; Dikarya; Basidiomycota; Agaricomycotina; Agaricomycetes; Agaricomycetidae; Agaricales; Agaricaceae; Leucoagaricus<br>Chromosome Unknown, exon count: 12 (KXN86269) | cd08190<br>KXN86269<br>(AN958_10131)<br>510 aa |
| *Amanita muscaria Koide* BX008<br>Eukaryota; Fungi; Dikarya; Basidiomycota; Agaricomycotina; Agaricomycetes; Agaricomycetidae; Agaricales; Amanitaceae; Amanita<br>Chromosome Unknown, exon count: 13 (KIL70062) | cd08190<br>KIL70062<br>(M378DRAFT_96258)<br>491 aa |
| *Hebeloma cylindrosporum* h7<br>Eukaryota; Fungi; Dikarya; Basidiomycota; Agaricomycotina; Agaricomycetes; Agaricomycetidae; Agaricales; Cortinariaceae; Hebeloma<br>Chromosome Unknown, exon count: 12 (KIM46190) | cd08190<br>KIM46190<br>(M413DRAFT_441262)<br>493 aa |
| *Fistulina hepatica* ATCC 64428<br>Eukaryota; Fungi; Dikarya; Basidiomycota; Agaricomycotina; Agaricomycetes; Agaricomycetidae; Agaricales; Fistulinaceae; Fistulina<br>Chromosome Unknown, exon count: 12 (KIY49198) | cd08190<br>KIY49198<br>(FISHEDRAFT_41233)<br>507 aa |
| *Hypsizygus marmoreus* 51987-8<br>Eukaryota; Fungi; Dikarya; Basidiomycota; Agaricomycotina; Agaricomycetes; Agaricomycetidae; Agaricales; Lyophyllaceae; Hypsizygus<br>Chromosome Unknown, exon count: 13 (KYQ42544) | cd08190<br>KYQ42544<br>(Hypma_03629)<br>492 aa |
| *Termitomyces* sp. J132<br>Eukaryota; Fungi; Dikarya; Basidiomycota; Agaricomycotina; Agaricomycetes; Agaricomycetidae; Agaricales; Lyophyllaceae; Termitomyces<br>Chromosome Unknown, exon count: 13 (KNZ79801) | cd08190<br>KNZ79801<br>(J132_08459)<br>492 aa |
| *Moniliophthora perniciosa FA553*<br>Eukaryota; Fungi; Dikarya; Basidiomycota; Agaricomycotina; Agaricomycetes; Agaricomycetidae; Agaricales; Marasmiaceae; mitosporic Marasmiaceae; Moniliophthora<br>Chromosome Unknown, exon count: >6 (XP_002396519) | cd08190<br>XP_002396519<br>(MPER_03230)<br>238 aa (corrected fragment) |
| *Moniliophthora roreri* MCA 2997<br>Eukaryota; Fungi; Dikarya; Basidiomycota; Agaricomycotina; Agaricomycetes; Agaricomycetidae; Agaricales; Marasmiaceae; mitosporic Marasmiaceae; Moniliophthora<br>Chromosome Unknown, exon count: 12 (XP_007844595) | cd08190<br>XP_007844595<br>(Moror_7341)<br>492 aa |
| *Gymnopus luxurians* FD-317 M1<br>Eukaryota; Fungi; Dikarya; Basidiomycota; Agaricomycotina; Agaricomycetes; Agaricomycetidae; Agaricales; Omphalotaceae; Gymnopus<br>Chromosome Unknown, exon count: 14 (KIK68170) | cd08190<br>KIK68170<br>(GYMLUDRAFT_37010)<br>449 aa |
| *Pleurotus ostreatus* PC15<br>Eukaryota; Fungi; Dikarya; Basidiomycota; Agaricomycotina; Agaricomycetes; Agaricomycetidae; Agaricales; Pleurotaceae; Pleurotus<br>Chromosome Unknown, exon count: 13 (KDQ28960) | cd08190<br>KDQ28960<br>(PLEOSDRAFT_1102997)<br>492 aa |
| *Coprinopsis cinerea okayama7#130 (Coprinus cinereus okayama7#130)*<br>Eukaryota; Fungi; Dikarya; Basidiomycota; Agaricomycotina; Agaricomycetes; Agaricomycetidae; Agaricales; Psathyrellaceae; Coprinopsis<br>Chromosome 5, exon count: 13 (XP_001837165) | cd08190<br>XP_001837165<br>CC1G_00301)<br>492 aa |
| *Schizophyllum commune* H4-8<br>Eukaryota; Fungi; Dikarya; Basidiomycota; Agaricomycotina; Agaricomycetes; Agaricomycetidae; Agaricales; Schizophyllaceae; Schizophyllum<br>Chromosome Unknown, exon count: 11 (XP_003036478) | cd08190<br>XP_003036478<br>(SCHCODRAFT_71701)<br>494 aa |
| *Galerina marginata* CBS 339.88<br>Eukaryota; Fungi; Dikarya; Basidiomycota; Agaricomycotina; Agaricomycetes; Agaricomycetidae; Agaricales; Strophariaceae; Galerina<br>Chromosome Unknown, exon count: 13 (KDR83744) | cd08190<br>KDR83744<br>(GALMADRAFT_236099)<br>493 aa |
| *Hypholoma sublateritium* FD-334 SS-4<br>Eukaryota; Fungi; Dikarya; Basidiomycota; Agaricomycotina; Agaricomycetes; Agaricomycetidae; Agaricales; Strophariaceae; Hypholoma<br>Chromosome Unknown, exon count: 13 (KJA24478) | cd08190<br>KJA24478<br>(HYPSUDRAFT_1074679)<br>493 aa |
| *Laccaria amethystina* LaAM-08-1<br>Eukaryota; Fungi; Dikarya; Basidiomycota; Agaricomycotina; Agaricomycetes; Agaricomycetidae; Agaricales; Tricholomataceae; Laccaria<br>Chromosome Unknown, exon count: 13 (KIK06711) | cd08190<br>KIK06711<br>(K443DRAFT_129650)<br>488 aa |

| Organism[1]<br>Lineage<br>Chromosome location and exon count | Fe-ADH family (conserved Domain Database)<br>Protein accesion number<br>(Gene locus)<br>Amino acid sequence lenght |
|---|---|
| *Laccaria bicolor* S238N-H82<br>Eukaryota; Fungi; Dikarya; Basidiomycota; Agaricomycotina; Agaricomycetes; Agaricomycetidae; Agaricales; Tricholomataceae; Laccaria<br>Chromosome Unknown, exon count: 9 (XP_001880100)<br>Chromosome Unknown, exon count: 13 (XP_001876672) | Cd08177<br>XP_001880100<br>(LACBIDRAFT_386226)<br>418 aa<br><br>cd08190<br>XP_001876672<br>(LACBIDRAFT_311781)<br>488 aa |
| *Plicaturopsis crispa* FD-325 SS-3<br>Eukaryota; Fungi; Dikarya; Basidiomycota; Agaricomycotina; Agaricomycetes; Agaricomycetidae; Agaricales; Agaricales incertae sedis; Plicaturopsis<br>Chromosome Unknown, exon count: 9 (KII95205) | cd08190<br>KII95205<br>(PLICRDRAFT_96252)<br>495 aa |
| *Piloderma croceum* F 1598<br>Eukaryota; Fungi; Dikarya; Basidiomycota; Agaricomycotina; Agaricomycetes; Agaricomycetidae; Atheliales; Atheliaceae; Piloderma<br>Chromosome Unknown, exon count: 12 (KIM90795) | cd08190<br>KIM90795<br>(PILCRDRAFT_94495)<br>491 aa |
| *Coniophora puteana* RWD-64-598 SS2<br>Eukaryota; Fungi; Dikarya; Basidiomycota; Agaricomycotina; Agaricomycetes; Agaricomycetidae; Boletales; Coniophorineae; Coniophoraceae; Coniophora<br>Chromosome Unknown, exon count: 12 (XP_007773383) | cd08190<br>XP_007773383<br>EIW76109<br>(CONPUDRAFT_130475)<br>495 aa |
| *Serpula lacrymans* var. *lacrymans* S7.9<br>Eukaryota; Fungi; Dikarya; Basidiomycota; Agaricomycotina; Agaricomycetes; Agaricomycetidae; Boletales; Coniophorineae; Serpulaceae; Serpula<br>Chromosome Unknown, exon count: 11 (XP_007315972) | cd08190<br>XP_007315972<br>(SERLADRAFT_462073)<br>495 aa |
| *Hydnomerulius pinastri* MD-312<br>Eukaryota; Fungi; Dikarya; Basidiomycota; Agaricomycotina; Agaricomycetes; Agaricomycetidae; Boletales; Paxilineae; Paxillaceae; Hydnomerulius<br>Chromosome Unknown, exon count: 12 (KIJ70567) | cd08190<br>KIJ70567<br>(HYDPIDRAFT_172357)<br>497 aa |
| *Paxillus involutus* ATCC 200175<br>Eukaryota; Fungi; Dikarya; Basidiomycota; Agaricomycotina; Agaricomycetes; Agaricomycetidae; Boletales; Paxilineae; Paxillaceae; Paxillus<br>Chromosome Unknown, exon count: 12 (KIJ20018) | cd08190<br>KIJ20018<br>(PAXINDRAFT_126399)<br>497 aa |
| *Paxillus rubicundulus* Ve08.2h10<br>Eukaryota; Fungi; Dikarya; Basidiomycota; Agaricomycotina; Agaricomycetes; Agaricomycetidae; Boletales; Paxilineae; Paxillaceae; Paxillus<br>Chromosome Unknown, exon count: 12 (KIK97872) | cd08190<br>KIK97872<br>(PAXRUDRAFT_824499)<br>497 aa |
| *Pisolithus microcarpus* 441<br>Eukaryota; Fungi; Dikarya; Basidiomycota; Agaricomycotina; Agaricomycetes; Agaricomycetidae; Boletales; Sclerodermatineae; Pisolithaceae; Pisolithus<br>Chromosome Unknown, exon count: 12 (KIK30686) | cd08190<br>KIK30686<br>(PISMIDRAFT_306255)<br>494 aa |
| *Pisolithus tinctorius* Marx 270<br>Eukaryota; Fungi; Dikarya; Basidiomycota; Agaricomycotina; Agaricomycetes; Agaricomycetidae; Boletales; Sclerodermatineae; Pisolithaceae; Pisolithus<br>Chromosome Unknown, exon count: 12 (KIO12955) | cd08190<br>KIO12955<br>(M404DRAFT_123702)<br>494 aa |
| *Scleroderma citrinum* Foug A<br>Eukaryota; Fungi; Dikarya; Basidiomycota; Agaricomycotina; Agaricomycetes; Agaricomycetidae; Boletales; Sclerodermatineae; Sclerodermataceae; Scleroderma<br>Chromosome Unknown, exon count: 12 (KIM53799) | cd08190<br>KIM53799<br>(SCLCIDRAFT_17831)<br>492 aa |
| *Suillus luteus* UH-Slu-Lm8-n1<br>Eukaryota; Fungi; Dikarya; Basidiomycota; Agaricomycotina; Agaricomycetes; Agaricomycetidae; Boletales; Suillineae; Suillaceae; Suillus<br>Chromosome Unknown, exon count: 11 (KIK48910) | cd08190<br>KIK48910<br>(CY34DRAFT_517633)<br>496 aa |
| *Jaapia argillacea* MUCL 33604<br>Eukaryota; Fungi; Dikarya; Basidiomycota; Agaricomycotina; Agaricomycetes; Agaricomycetidae; Jaapiales; Jaapiaceae; Jaapia<br>Chromosome Unknown, exon count: 12 (KDQ64499) | cd08190<br>KDQ64499<br>(JAAARDRAFT_28128)<br>492 aa |
| *Auricularia subglabra* TFB-10046 SS5<br>Eukaryota; Fungi; Dikarya; Basidiomycota; Agaricomycotina; Agaricomycetes; Auriculariales; Auriculariaceae; Auricularia<br>Chromosome Unknown, exon count: 14 (XP_007343343) | cd08190<br>XP_007343343<br>(AURDEDRAFT_112896<br>494 aa |
| *Botryobasidium botryosum* FD-172 SS1<br>Eukaryota; Fungi; Dikarya; Basidiomycota; Agaricomycotina; Agaricomycetes; Cantharellales; Botryobasidiaceae; Botryobasidium<br>Chromosome Unknown, exon count: 14 (KDQ15396) | cd08190<br>KDQ15396<br>(BOTBODRAFT_108650)<br>492 aa |
| *Rhizoctonia solani* AG-1 IB<br>Eukaryota; Fungi; Dikarya; Basidiomycota; Agaricomycotina; Agaricomycetes; Cantharellales; Ceratobasidiaceae; mitosporic Ceratobasidiaceae; Rhizoctonia<br>Chromosome Unknown, exon count: 14 (CEL63653) | cd08190<br>CEL63653<br>(RSOLAG1IB_05414)<br>493 aa |

| Organism[1]<br>Lineage<br>Chromosome location and exon count | Fe-ADH family (conserved Domain Database)<br>Protein accesion number<br>(Gene locus)<br>Amino acid sequence lenght |
|---|---|
| *Rhizoctonia solani* AG-3 Rhs1AP<br>Eukaryota; Fungi; Dikarya; Basidiomycota; Agaricomycotina; Agaricomycetes; Cantharellales; Ceratobasidiaceae; mitosporic Ceratobasidiaceae; Rhizoctonia<br>Chromosome Unknown, exon count: 15 (EUC66454) | cd08190<br>EUC66454<br>(RSOL_475830)<br>493 aa |
| *Tulasnella calospora* MUT 4182<br>Eukaryota; Fungi; Dikarya; Basidiomycota; Agaricomycotina; Agaricomycetes; Cantharellales; Tulasnellaceae; Tulasnella<br>Chromosome Unknown, exon count: 11 (KIO31048) | cd08190<br>KIO31048<br>(M407DRAFT_68432)<br>493 aa |
| *Cylindrobasidium torrendii* FP15055 ss-10<br>Eukaryota; Fungi; Dikarya; Basidiomycota; Agaricomycotina; Agaricomycetes; Corticiales; Corticiaceae; Cylindrobasidium<br>Chromosome Unknown, exon count: 9 (KIY68873) | cd08190<br>KIY68873<br>(CYLTODRAFT_421207)<br>492 aa |
| *Schizopora paradoxa*<br>Eukaryota; Fungi; Dikarya; Basidiomycota; Agaricomycotina; Agaricomycetes; Corticiales; Corticiaceae; Schizopora<br>Chromosome Unknown, exon count: 11 (KLO19947) | cd08190<br>KLO19947<br>(SCHPADRAFT_47797)<br>494 aa |
| *Punctularia strigosozonata* HHB-11173 SS5<br>Eukaryota; Fungi; Dikarya; Basidiomycota; Agaricomycotina; Agaricomycetes; Corticiales; Punctulariaceae; Punctularia<br>Chromosome Unknown, exon count: 16 (XP_007382257) | cd08190<br>XP_007382257<br>(PUNSTDRAFT_100490)<br>494 aa |
| *Gloeophyllum trabeum* ATCC 11539<br>Eukaryota; Fungi; Dikarya; Basidiomycota; Agaricomycotina; Agaricomycetes; Gloeophyllales; Gloeophyllaceae; Gloeophyllum<br>Chromosome Unknown, exon count: 12 (XP_007862281) | cd08190<br>XP_007862281<br>(GLOTRDRAFT_136160)<br>493 aa |
| *Fomitiporia mediterranea* MF3/22<br>Eukaryota; Fungi; Dikarya; Basidiomycota; Agaricomycotina; Agaricomycetes; Hymenochaetales; Hymenochaetaceae; Fomitiporia<br>Chromosome Unknown, exon count: 11 (XP_007268780) | cd08190<br>XP_007268780<br>(FOMMEDRAFT_147995)<br>510 aa |
| *Phanerochaete carnosa* HHB-10118-sp<br>Eukaryota; Fungi; Dikarya; Basidiomycota; Agaricomycotina; Agaricomycetes; Polyporales; Phanerochaetaceae; Phanerochaete<br>Chromosome Unknown, exon count: 10 (XP_007391904) | cd08190<br>XP_007391904<br>(PHACADRAFT_249766)<br>496 aa |
| *Phlebiopsis gigantea* 11061_1 CR5-6<br>Eukaryota; Fungi; Dikarya; Basidiomycota; Agaricomycotina; Agaricomycetes; Polyporales; Phanerochaetaceae; Phlebiopsis<br>Chromosome Unknown, exon count: 9 (KIP12707) | cd08190<br>KIP12707<br>(PHLGIDRAFT_123800)<br>495 aa |
| *Dichomitus squalens* LYAD-421 SS1<br>Eukaryota; Fungi; Dikarya; Basidiomycota; Agaricomycotina; Agaricomycetes; Polyporales; Polyporaceae; Dichomitus<br>Chromosome Unknown, exon count: 12 (XP_007366111) | cd08190<br>XP_007366111<br>(DICSQDRAFT_136849)<br>496 aa |
| *Fibroporia radiculosa*<br>Eukaryota; Fungi; Dikarya; Basidiomycota; Agaricomycotina; Agaricomycetes; Polyporales; Polyporaceae; Fibroporia<br>Chromosome Unknown, exon count: 12 (XP_012184076) | cd08190<br>XP_012184076<br>(FIBRA_06986)<br>495 aa |
| *Postia placenta* Mad-698-R<br>Eukaryota; Fungi; Dikarya; Basidiomycota; Agaricomycotina; Agaricomycetes; Polyporales; Postia<br>Chromosome Unknown, exon count: 13 (XP_002475474) | cd08190<br>XP_002475474<br>(POSPLDRAFT_104120)<br>429 aa |
| *Fomitopsis pinicola* FP-58527 SS1<br>Eukaryota; Fungi; Dikarya; Basidiomycota; Agaricomycotina; Agaricomycetes; Polyporales; Fomitopsis<br>Chromosome Unknown, exon count: 9 (EPS99233)<br>Chromosome Unknown, exon count: 9 (EPT02717) | cd08190<br>EPS99233<br>(FOMPIDRAFT_1124646)<br>(Fompi3_1124646)<br>493 aa<br><br>cd08190<br>EPT02717<br>(FOMPIDRAFT_1022658)<br>(Fompi3_1022658)<br>496 aa |
| *Gelatoporia subvermispora* B<br>Eukaryota; Fungi; Dikarya; Basidiomycota; Agaricomycotina; Agaricomycetes; Polyporales; Gelatoporia<br>Chromosome Unknown, exon count: 11 (EMD40675) | cd08190<br>EMD40675<br>(CERSUDRAFT_111255)<br>514 aa |
| *Taiwanofungus camphoratus* (*Antrodia camphorata*)<br>Eukaryota; Fungi; Dikarya; Basidiomycota; Agaricomycotina; Agaricomycetes; Polyporales; Taiwanofungus<br>Chromosome Unknown, exon count: Unknown (AFF60483) | cd08190<br>AFF60483<br>(--)<br>496 aa |
| *Trametes cinnabarina*<br>Eukaryota; Fungi; Dikarya; Basidiomycota; Agaricomycotina; Agaricomycetes; Polyporales; Trametes<br>Chromosome Unknown, exon count: 10 (CDO76248) | cd08190<br>CDO76248<br>(BN946_scf184470.g6)<br>454 aa |

| Organism[1]<br>Lineage<br>Chromosome location and exon count | Fe-ADH family (conserved Domain Database)<br>Protein accesion number<br>(Gene locus)<br>Amino acid sequence lenght |
|---|---|
| *Trametes versicolor* FP-101664 SS1<br>Eukaryota; Fungi; Dikarya; Basidiomycota; Agaricomycotina; Agaricomycetes; Polyporales; Trametes<br>Chromosome Unknown, exon count: 12 (XP_008035327) | cd08190<br>XP_008035327<br>(TRAVEDRAFT_69690)<br>496 aa |
| *Sphaerobolus stellatus* SS14<br>Eukaryota; Fungi; Dikarya; Basidiomycota; Agaricomycotina; Agaricomycetes; Phallomycetidae; Geastrales; Sphaerobolaceae; Sphaerobolus.<br>Chromosome Unknown, exon count: 1 (KIJ30521)<br>Chromosome Unknown, exon count: 14 (KIJ57033) | CD08177<br>KIJ30521<br>(M422DRAFT_187040)<br>359 aa<br><br>cd08190<br>KIJ57033<br>(M422DRAFT_40580)<br>504 aa |
| *Heterobasidion irregulare* TC 32-1<br>Eukaryota; Fungi; Dikarya; Basidiomycota; Agaricomycotina; Agaricomycetes; Russulales; Bondarzewiaceae; Heterobasidion; Heterobasidion annosum species complex<br>Chromosome Unknown, exon count: 14 (XP_009543776) | cd08190<br>XP_009543776<br>(HETIRDRAFT_381405)<br>491 aa |
| *Stereum hirsutum* FP-91666 SS1<br>Eukaryota; Fungi; Dikarya; Basidiomycota; Agaricomycotina; Agaricomycetes; Russulales; Stereaceae; Stereum<br>Chromosome Unknown, exon count: 11 (XP_007305655) | cd08190<br>XP_007305655<br>(STEHIDRAFT_147985)<br>491 aa |
| *Serendipita indica* DSM 11827 (*Piriformospora indica* DSM 11827)<br>Eukaryota; Fungi; Dikarya; Basidiomycota; Agaricomycotina; Agaricomycetes; Sebacinales; Serendipitaceae; Serendipita<br>Chromosome Unknown, exon count: 7 (CCA68123) | cd08190<br>CCA68123<br>(PIIN_01990)<br>493 aa |
| *Serendipita vermifera* MAFF 305830 (*Sebacina vermifera* MAFF 305830)<br>Eukaryota; Fungi; Dikarya; Basidiomycota; Agaricomycotina; Agaricomycetes; Sebacinales; Serendipitaceae; Serendipita<br>Chromosome Unknown, exon count: 8 (KIM25870) | cd08190<br>KIM25870<br>(M408DRAFT_330877)<br>493 aa |
| *Dacryopinax primogenitus* DJM-731 SS1<br>Eukaryota; Fungi; Dikarya; Basidiomycota; Agaricomycotina; Dacrymycetes; Dacrymycetales; Dacrymycetaceae; Dacryopinax<br>Chromosome Unknown, exon count: 8 (EJU06564) | cd08190<br>EJU06564<br>DACRYDRAFT_92508)<br>501 aa |
| *Xanthophyllomyces dendrorhous*<br>Eukaryota; Fungi; Dikarya; Basidiomycota; Agaricomycotina; Tremellomycetes; Cystofilobasidiales; Cystofilobasidiaceae; Xanthophyllomyces<br>Chromosome Unknown, exon count: 14 (CDZ98808) | cd08190<br>CDZ98808<br>(--)<br>518 aa |
| *Cryptococcus gattii* VGII CA1014 (*Cryptococcus deuterogattii* CA1014)<br>Eukaryota; Fungi; Dikarya; Basidiomycota; Agaricomycotina; Tremellomycetes; Tremellales; Cryptococcaceae; Cryptococcus; Cryptococcus gattii species complex<br>Chromosome Unknown, exon count: 9 (KIR74373) | cd08190<br>KIR74373<br>(I310_01980)<br>519 aa |
| *Cryptococcus gattii* CA1280 (*Cryptococcus bacillisporus* CA1280)<br>Eukaryota; Fungi; Dikarya; Basidiomycota; Agaricomycotina; Tremellomycetes; Tremellales; Cryptococcaceae; Cryptococcus; Cryptococcus gattii species complex<br>Chromosome Unknown, exon count: 9 (KIR50258) | cd08190<br>KIR50258<br>(I312_00189)<br>519 aa |
| *Cryptococcus gattii* VGIV IND107 (*Cryptococcus tetragattii* IND107)<br>Eukaryota; Fungi; Dikarya; Basidiomycota; Agaricomycotina; Tremellomycetes; Tremellales; Cryptococcaceae; Cryptococcus; Cryptococcus gattii species complex<br>Chromosome Unknown, exon count: 9 (KIR87684) | cd08190<br>KIR87684<br>(I308_01706)<br>519 aa |
| *Cryptococcus gattii* VGII LA55 (*Cryptococcus deuterogattii* LA55)<br>Eukaryota; Fungi; Dikarya; Basidiomycota; Agaricomycotina; Tremellomycetes; Tremellales; Cryptococcaceae; Cryptococcus; Cryptococcus gattii species complex<br>Chromosome Unknown, exon count: 9 (KIR25635) | cd08190<br>KIR25635<br>(I309_05574)<br>519 aa |
| *Cryptococcus gattii* VGII MMRL2647 (*Cryptococcus deuterogattii* MMRL2647)<br>Eukaryota; Fungi; Dikarya; Basidiomycota; Agaricomycotina; Tremellomycetes; Tremellales; Cryptococcaceae; Cryptococcus; Cryptococcus gattii species complex<br>Chromosome Unknown, exon count: 9 (KIR33757) | cd08190<br>KIR33757<br>(I352_03834)<br>519 aa |
| *Cryptococcus gattii* Ru294<br>Eukaryota; Fungi; Dikarya; Basidiomycota; Agaricomycotina; Tremellomycetes; Tremellales; Tremellaceae; Filobasidiella; Filobasidiella/Cryptococcus neoformans species complex<br>Chromosome Unknown, exon count: 9 (KIR55777) | cd08190<br>KIR55777<br>(I315_01659)<br>519 aa |
| *Cryptococcus gattii* WM276<br>Eukaryota; Fungi; Dikarya; Basidiomycota; Agaricomycotina; Tremellomycetes; Tremellales; Tremellaceae; Filobasidiella; Filobasidiella/Cryptococcus neoformans species complex<br>Chromosome B, exon count: 9 (XP_003192075) | cd08190<br>XP_003192075<br>(CGB_B2250C)<br>519 aa |
| *Cryptococcus neoformans* var. grubii H99<br>Eukaryota; Fungi; Dikarya; Basidiomycota; Agaricomycotina; Tremellomycetes; Tremellales; Tremellaceae; Filobasidiella; Filobasidiella/Cryptococcus neoformans species complex<br>Chromosome 1, exon count: 9 (XP_012047020) | cd08190<br>XP_012047020<br>(CNAG_07316)<br>519 aa |

| Organism[1]<br>Lineage<br>Chromosome location and exon count | Fe-ADH family (conserved Domain Database)<br>Protein accesion number<br>(Gene locus)<br>Amino acid sequence lenght |
|---|---|
| *Cryptococcus neoformans* var. *neoformans* B-3501A<br>Eukaryota; Fungi; Dikarya; Basidiomycota; Agaricomycotina; Tremellomycetes; Tremellales; Tremellaceae; Filobasidiella; Filobasidiella/Cryptococcus neoformans species complex<br>Chromosome 1, exon count: 9 (XP_778178) | cd08190<br>XP_778178<br>(CNBA1780)<br>519 aa |
| *Cryptococcus neoformans* var. *neoformans* JEC21<br>Eukaryota; Fungi; Dikarya; Basidiomycota; Agaricomycotina; Tremellomycetes; Tremellales; Tremellaceae; Filobasidiella; Filobasidiella/Cryptococcus neoformans species complex<br>Chromosome 1, exon count: 9 (XP_566553) | cd08190<br>XP_566553<br>(CNA01850)<br>519 aa |
| *Trichosporon asahii* var. *asahii* CBS 2479<br>Eukaryota; Fungi; Dikarya; Basidiomycota; Agaricomycotina; Tremellomycetes; Tremellales; Tremellales incertae sedis; Trichosporon<br>Chromosome Unknown, exon count: 3 (XP_014180536)<br>Chromosome Unknown, exon count: 3 (XP_014184467) | CD08177<br>XP_014180536<br>(A1Q1_02116)<br>357 aa<br><br>cd08190<br>XP_014184467<br>(A1Q1_00294)<br>513 aa |
| *Trichosporon asahii* var. *asahii* CBS 8904<br>Eukaryota; Fungi; Dikarya; Basidiomycota; Agaricomycotina; Tremellomycetes; Tremellales; Tremellales incertae sedis; Trichosporon<br>Chromosome Unknown, exon count: 4 (EKC97630)<br>Chromosome Unknown, exon count: 4 (EKD02340) | CD08177<br>EKC97630<br>(A1Q2_08089)<br>373 aa<br><br>cd08190<br>EKD02340<br>(A1Q2_03344)<br>619 aa |
| *Tremella mesenterica* DSM 1558<br>Eukaryota; Fungi; Dikarya; Basidiomycota; Agaricomycotina; Tremellomycetes; Tremellales; Tremellaceae; Tremella<br>Chromosome Unknown, exon count: 9 (XP_007003705) | cd08190<br>XP_007003705<br>(TREMEDRAFT_68204)<br>512 aa |
| *Trichosporon oleaginosus*<br>Eukaryota; Fungi; Dikarya; Basidiomycota; Agaricomycotina; Tremellomycetes; Tremellales; Trichosporonaceae; Trichosporon<br>Chromosome Unknown, exon count: 3 (KLT42783) | cd08190<br>KLT42783<br>(CC85DRAFT_285125)<br>511 aa |
| *Rhodotorula* sp. JG-1b<br>Eukaryota; Fungi; Dikarya; Basidiomycota; mitosporic Basidiomycota; Rhodotorula<br>Chromosome Unknown, exon count: 14 (KWU41559) | cd08190<br>KWU41559<br>(RHOSPDRAFT_36870)<br>514 aa |
| *Rhodotorula graminis* WP1<br>Eukaryota; Fungi; Dikarya; Basidiomycota; Pucciniomycotina; Microbotryomycetes; Sporidiobolales; Sporidiobolaceae; Rhodotorula<br>Chromosome Unknown, exon count: 13 (KPV77218) | cd08190<br>KPV77218<br>(RHOBADRAFT_34718)<br>489 aa |
| *Rhodotorula toruloides* NP11<br>Eukaryota; Fungi; Dikarya; Basidiomycota; Pucciniomycotina; Microbotryomycetes; Sporidiobolales; Sporidiobolaceae; Rhodotorula<br>Chromosome Unknown, exon count: 10 (EMS22096) | cd08190<br>EMS22096<br>XP_016273215<br>(RHTO_01311)<br>525 aa |
| *Microbotryum lychnidis-dioicae* p1A1 Lamole<br>Eukaryota; Fungi; Dikarya; Basidiomycota; Pucciniomycotina; Microbotryomycetes; Microbotryales; Microbotryaceae; Microbotryum<br>Chromosome Unknown, exon count: 9 (KDE03823) | cd08190<br>KDE03823<br>(MVLG_05707)<br>530 aa |
| *Sporidiobolus salmonicolor*<br>Eukaryota; Fungi; Dikarya; Basidiomycota; Pucciniomycotina; Microbotryomycetes; Sporidiobolales; Sporidiobolaceae; Sporidiobolus<br>Chromosome Unknown, exon count: 11 (CEQ39230) | cd08190<br>CEQ39230<br>(SPOSA6832_00741)<br>507 aa |
| *Mixia osmundae* IAM 14324<br>Eukaryota; Fungi; Dikarya; Basidiomycota; Pucciniomycotina; Mixiomycetes; Mixiales; Mixiaceae; Mixia<br>Chromosome Unknown, exon count: 4 (XP_014570799) | cd08190<br>XP_014570799<br>(L969DRAFT_91631)<br>500 aa |
| *Melampsora larici-populina* 98AG31<br>Eukaryota; Fungi; Dikarya; Basidiomycota; Pucciniomycotina; Pucciniomycetes; Pucciniales; Melampsoraceae; Melampsora<br>Chromosome Unknown, exon count: 10 (XP_007412317)<br>Chromosome Unknown, exon count: 4 (XP_007416729) | cd08190<br>XP_007412317<br>MELLADRAFT_44184<br>502 aa<br><br>cd08190<br>XP_007416729<br>(MELLADRAFT_112213)<br>158 aa<br>Probably not functional (not included in phylogenetic analysis). |

| Organism[1]<br>Lineage<br>Chromosome location and exon count | Fe-ADH family (conserved Domain Database)<br>Protein accesion number<br>(Gene locus)<br>Amino acid sequence lenght |
|---|---|
| *Puccinia graminis* f. sp. *tritici* CRL 75-36-700-3<br>Eukaryota; Fungi; Dikarya; Basidiomycota; Pucciniomycotina; Pucciniomycetes; Pucciniales; Pucciniaceae; Puccinia<br>Chromosome Unknown, exon count: 10 (XP_003890301) | cd08190<br>XP_003890301<br>(PGTG_21040)<br>501 aa (corrected) |
| *Puccinia sorghi*<br>Eukaryota; Fungi; Dikarya; Basidiomycota; Pucciniomycotina; Pucciniomycetes; Pucciniales; Pucciniaceae; Puccinia<br>Chromosome Unknown, exon count: 11 (KNZ46068) | cd08190<br>KNZ46068<br>(VP01_758g5)<br>418 aa |
| *Puccinia striiformis* f. sp. *tritici* PST-78<br>Eukaryota; Fungi; Dikarya; Basidiomycota; Pucciniomycotina; Pucciniomycetes; Pucciniales; Pucciniaceae; Puccinia<br>Chromosome Unknown, exon count: 10 (KNE91206) | cd08190<br>KNE91206<br>(PSTG_15370)<br>500 aa |
| *Ceraceosorus bombacis*<br>Eukaryota; Fungi; Dikarya; Basidiomycota; Ustilaginomycotina; Exobasidiomycetes; Ceraceosorales; Ceraceosoraceae; Ceraceosorus<br>Chromosome Unknown, exon count: 3 (CEH17239) | cd08190<br>CEH17239<br>(CBOM_03297)<br>511 aa |
| *Tilletiaria anomala* UBC 951<br>Eukaryota; Fungi; Dikarya; Basidiomycota; Ustilaginomycotina; Exobasidiomycetes; Georgefischeriales; Tilletiariaceae; Tilletiaria<br>Chromosome Unknown, exon count: 6 (XP_013243686) | cd08190<br>XP_013243686<br>(K437DRAFT_256098)<br>509 aa |
| *Anthracocystis flocculosa* PF-1 (*Pseudozyma flocculosa* PF-1)<br>Eukaryota; Fungi; Dikarya; Basidiomycota; Ustilaginomycotina; Ustilaginomycetes; Ustilaginales; Ustilaginaceae; Anthracocystis<br>Chromosome Unknown, exon count: 3 (XP_007879979) | cd08190<br>XP_007879979<br>(PFL1_04265)<br>510 aa |
| *Kalmanozyma brasiliensis* GHG001<br>Eukaryota; Fungi; Dikarya; Basidiomycota; Ustilaginomycotina; Ustilaginomycetes; Ustilaginales; Ustilaginaceae; Kalmanozyma<br>Chromosome Unknown, exon count: 1 (EST04841) | cd08190<br>EST04841<br>(PSEUBRA_SCAF8g02240)<br>510 aa |
| *Melanopsichium pennsylvanicum* 4<br>Eukaryota; Fungi; Dikarya; Basidiomycota; Ustilaginomycotina; Ustilaginomycetes; Ustilaginales; Ustilaginaceae; Melanopsichium.<br>Chromosome Unknown, exon count: 1 (CDI54649)<br>Chromosome Unknown, exon count: 1 (CDI53318) | cd08177<br>CDI54649<br>(BN887_01363)<br>417 aa<br><br>cd08190<br>CDI53318<br>(BN887_02914)<br>510 aa |
| *Moesziomyces aphidis* DSM 70725<br>Eukaryota; Fungi; Dikarya; Basidiomycota; Ustilaginomycotina; Ustilaginomycetes; Ustilaginales; Ustilaginaceae; Moesziomyces<br>Chromosome Unknown, exon count: 4 (ETS59493) | cd08190<br>ETS59493<br>(PaG_06412)<br>1086 aa (FeADH domain located at residues 577-1086) |
| *Moesziomyces antarcticus* (*Pseudozyma antarctica*)<br>Eukaryota; Fungi; Dikarya; Basidiomycota; Ustilaginomycotina; Ustilaginomycetes; Ustilaginales; Ustilaginaceae; Moesziomyces<br>Chromosome Unknown, exon count: 4 (XP_014656425) | cd08190<br>XP_014656425<br>(PAN0_008c3438)<br>572 aa (FeADH domain located at residues 60-572) |
| *Pseudozyma hubeiensis* SY62<br>Eukaryota; Fungi; Dikarya; Basidiomycota; Ustilaginomycotina; Ustilaginomycetes; Ustilaginales; Ustilaginaceae; mitosporic Ustilaginaceae; Pseudozyma<br>Chromosome Unknown, exon count: 2 (XP_012188027) | cd08190<br>XP_012188027<br>(PHSY_002011)<br>743 aa (FeADH domain located at residues 1-515) |
| *Sporisorium reilianum SRZ2*<br>Eukaryota; Fungi; Dikarya; Basidiomycota; Ustilaginomycotina; Ustilaginomycetes; Ustilaginales; Ustilaginaceae; Sporisorium<br>Chromosome Unknown, exon count: 2 (CBQ72809) | cd08190<br>CBQ72809<br>(sr13430)<br>510 aa |
| *Sporisorium scitamineum*<br>Eukaryota; Fungi; Dikarya; Basidiomycota; Ustilaginomycotina; Ustilaginomycetes; Ustilaginales; Ustilaginaceae; Sporisorium<br>Chromosome Unknown, exon count: 2 (CDW97096) | cd08190<br>CDW97096<br>(SSCI23540.1)<br>510 aa |
| *Ustilago hordei*<br>Eukaryota; Fungi; Dikarya; Basidiomycota; Ustilaginomycotina; Ustilaginomycetes; Ustilaginales; Ustilaginaceae; Ustilago<br>Chromosome 5, exon count: 2 (CCF52126) | cd08190<br>CCF52126<br>(UHOR_03673)<br>508 aa |
| *Ustilago maydis* 521<br>Eukaryota; Fungi; Dikarya; Basidiomycota; Ustilaginomycotina; Ustilaginomycetes; Ustilaginales; Ustilaginaceae; Ustilago<br>Chromosome 11, exon count: 1 (XP_760061)<br>Chromosome 5, exon count: 2 (XP_011388804) | cd08177<br>XP_760061<br>(UM03914.1)<br>418 aa<br><br>cd08190<br>XP_011388804<br>(UMAG_10077)<br>510 aa |

| Organism[1]<br>Lineage<br>Chromosome location and exon count | Fe-ADH family (conserved Domain Database)<br>Protein accesion number<br>(Gene locus)<br>Amino acid sequence lenght |
|---|---|
| *Wallemia ichthyophaga* EXF-994<br>Eukaryota; Fungi; Dikarya; Basidiomycota; Basidiomycota incertae sedis; Wallemiomycetes; Wallemiales; Wallemiales incertae sedis; Wallemia<br>Chromosome Unknown, exon count: 8 (XP_009266922) | cd08190<br>XP_009266922<br>(J056_003424)<br>491 aa |
| *Wallemia mellicola* CBS 633.66 (*Wallemia sebi* CBS 633.66)<br>Eukaryota; Fungi; Dikarya; Basidiomycota; Basidiomycota incertae sedis; Wallemiomycetes; Wallemiales; Wallemiales incertae sedis; Wallemia<br>Chromosome Unknown, exon count: 9 (XP_006957872) | cd08190<br>XP_006957872<br>(WALSEDRAFT_63809)<br>489 aa |
| Chytridiomycota | |
| *Gonapodya prolifera* JEL478<br>Eukaryota; Fungi; Chytridiomycota; Monoblepharidomycetes; Monoblepharidales; Gonapodyaceae; Gonapodya<br>Chromosome Unknown, exon count: 9 | CD08194<br>KXS18892<br>(M427DRAFT_53376)<br>403 aa |
| *Spizellomyces punctatus* DAOM BR117<br>Eukaryota; Fungi; Chytridiomycota; Chytridiomycetes; Spizellomycetales; Spizellomycetaceae; Spizellomyces<br>Chromosome Unknown, exon count: 9 (KNC96439) | cd08190<br>KNC96439<br>(SPPG_08032)<br>485 aa |
| Entomophthoromycota | |
| *Conidiobolus coronatus* NRRL 28638<br>Eukaryota; Fungi; Entomophthoromycota; Entomophthoromycetes; Entomophthorales; Ancylistaceae; Conidiobolus<br>Chromosome Unknown, exon count: 12 (KXN66843) | cd08190<br>KXN66843<br>(CONCODRAFT_11233)<br>474 aa |
| Neocallimastigomycota | |
| *Neocallimastix frontalis*<br>Eukaryota; Fungi; Neocallimastigomycota; Neocallimastigomycetes; Neocallimastigales; Neocallimastigaceae; Neocallimastix | CD08178<br>AFJ73504<br>(ACDH2)<br>885 aa (bidomain protein) |
| *Piromyces* sp. E2<br>Eukaryota; Fungi; Neocallimastigomycota; Neocallimastigomycetes; Neocallimastigales; Neocallimastigaceae; Piromyces | CD08178<br>AAQ22352<br>(adhE)<br>885 aa (bidomain protein) |
| Fungi incertae sedis | |
| *Lichtheimia ramosa*<br>Eukaryota; Fungi; Fungi incertae sedis; Mucoromycotina; Mucorales; Lichtheimiaceae; Lichtheimia<br>Chromosome Unknown, exon count: 5 (CDS13572) | cd08190<br>CDS13572<br>(LRAMOSA05748)<br>484 aa |
| *Mucor ambiguus*<br>Eukaryota; Fungi; Fungi incertae sedis; Mucoromycotina; Mucorales; Mucorineae; Mucoraceae; Mucor<br>Chromosome Unknown, exon count: 6 (GAN03077) | cd08190<br>GAN03077<br>(MAM1_0033c02528)<br>482 aa |
| *Mucor circinelloides* f. *circinelloides* 1006PhL<br>Eukaryota; Fungi; Fungi incertae sedis; Mucoromycotina; Mucorales; Mucorineae; Mucoraceae; Mucor<br>Chromosome Unknown, exon count: 6 (EPB85506) | cd08190<br>EPB85506<br>(HMPREF1544_07689)<br>482 aa |
| *Parasitella parasitica*<br>Eukaryota; Fungi; Fungi incertae sedis; Mucoromycotina; Mucorales; Mucorineae; Mucoraceae; Parasitella<br>Chromosome Unknown, exon count: 6 (CEP08392) | cd08190<br>CEP08392<br>(PARPA_01703.1 scaffold 1359)<br>482 aa |
| *Rhizopus delemar* RA 99-880<br>Eukaryota; Fungi; Fungi incertae sedis; Mucoromycotina; Mucorales; Mucorineae; Rhizopodaceae; Rhizopus<br>Chromosome Unknown, exon count: 6 (EIE84356) | cd08190<br>EIE84356<br>(RO3G_09066)<br>485 aa |
| *Rhizopus microsporus*<br>Eukaryota; Fungi; Fungi incertae sedis; Mucoromycotina; Mucorales; Mucorineae; Rhizopodaceae; Rhizopus<br>Chromosome Unknown, exon count: 5 (CEG71586) | cd08190<br>CEG71586<br>(RMATCC62417_07295)<br>482 aa |
| *Mortierella verticillata* NRRL 6337<br>Eukaryota; Fungi; Fungi incertae sedis; Mortierellomycotina; Mortierellales; Mortierellaceae; Mortierella<br>Chromosome Unknown, exon count: 2 (KFH69889) | cd08190<br>KFH69889<br>(MVEG_04693)<br>497 aa |

| Organism[1]<br>Lineage<br>Chromosome location and exon count | Fe-ADH family (conserved Domain Database)<br>Protein accesion number<br>(Gene locus)<br>Amino acid sequence lenght |
|---|---|
| Viridiplantae | |
| Chlorophyta | |
| *Chlamydomonas reinhardtii* (strain: CC-503 cw92 mt+)<br>Eukaryota; Viridiplantae; Chlorophyta; Chlorophyceae; Chlamydomonadales; Chlamydomonadaceae; Chlamydomonas<br>Chromosome Unknown, exon count: 17 (XP_001703585)<br>Chromosome Unknown, exon count: 7 (XP_001699167)<br>Chromosome Unknown, exon count: 10 (XP_001699226)<br>Las dos últimas proteínas posiblemente no sean funcionales porque el dominio de Fe-ADH no esta completo | CD08178<br>XP_001703585<br>(ADH1)<br>954 aa (bidomain protein)<br><br>CD08178<br>XP_001699167<br>CHLREDRAFT_82021<br>325 aa<br><br>CD08178<br>XP_001699226<br>CHLREDRAFT_121409<br>476 aa |
| *Polytomella* sp. Pringsheim 198.80<br>Eukaryota; Viridiplantae; Chlorophyta; Chlorophyceae; Chlamydomonadales; Chlamydomonadaceae; Polytomella<br>Chromosome Unknown, exon count: Unknown (sequenced from a mRNA) | CD08178<br>CAD42653<br>(adhE)<br>885 aa |
| *Volvox carteri* f. Nagariensis<br>Eukaryota; Viridiplantae; Chlorophyta; Chlorophyceae; Chlamydomonadales; Volvocaceae; Volvox<br>Chromosome Unknown, exon count: 20 (XP_002958353)<br>Chromosome Unknown, exon count: 34 (XP_002958348) | CD08178<br>XP_002958353<br>(adh1)<br>1105 aa (bidomain protein)<br><br>CD08178<br>XP_002958348<br>(VOLCADRAFT_119931)<br>2066 aa (multidomain protein) |
| *Gonium pectorale*<br>Eukaryota; Viridiplantae; Chlorophyta; Chlorophyceae; Chlamydomonadales; Volvocaceae; Gonium<br>Chromosome Unknown, exon count: 15 (KXZ52653)<br>Chromosome Unknown, exon count: 15 (KXZ52654)<br>Possess two gen copies in tandem | CD08178<br>KXZ52653<br>(GPECTOR_9g698)<br>951 aa (bidomain protein)<br><br>CD08178<br>KXZ52654<br>(GPECTOR_9g699)<br>961 aa (bidomain protein) |
| *Monoraphidium neglectum* SAG 48.87<br>Eukaryota; Viridiplantae; Chlorophyta; Chlorophyceae; Sphaeropleales; Selenastraceae; Monoraphidium<br>Chromosome Unknown, exon count: >5 | CD08178<br>XP_013890864<br>(MNEG_16119)<br>312 aa (corrected fragment of a bidomain protein) |
| *Chlorella variabilis* (strain: NC64A<br>Eukaryota; Viridiplantae; Chlorophyta; Trebouxiophyceae; Chlorellales; Chlorellaceae; Chlorella<br>Chromosome Unknown, exon count: 28 (XP_005850263)<br>Chromosome Unknown, exon count: 23 (XP_005845617) | CD08178<br>XP_005850263<br>EFN58161<br>(ADHE1)<br>947 aa (bidomain protein)<br><br>CD08178<br>XP_005845617<br>EFN53515<br>(ADHE2)<br>889 aa (bidomain protein) |
| *Auxenochlorella protothecoides* 0710 (*Chlorella protothecoides*)<br>Eukaryota; Viridiplantae; Chlorophyta; Trebouxiophyceae; Chlorellales; Chlorellaceae; Auxenochlorella<br>Chromosome Unknown, exon count: 4 | CD08176<br>XP_011400345<br>(F751_1155)<br>460 aa |
| *Micromonas pusilla* CCMP1545<br>Eukaryota; Viridiplantae; Chlorophyta; prasinophytes; Mamiellophyceae; Mamiellales; Mamiellaceae; Micromonas<br>Chromosome Unknown, exon count: 1(XP_003057250)<br>Chromosome Unknown, exon count: 1(XP_003059518) | CD08176<br>XP_003057250<br>(MICPUCDRAFT_10609)<br>448 aa (corrected)<br><br>CD08183<br>XP_003059518<br>(MICPUCDRAFT_58857)<br>487 aa |
| *Micromonas* sp. RCC299 (*Micromonas commoda*)<br>Eukaryota; Viridiplantae; Chlorophyta; Mamiellophyceae; Mamiellales; Micromonas<br>Chromosome 5, exon count: 1 | CD08183<br>XP_002502234<br>(MICPUN_81646)<br>406 aa |

| Organism[1]<br>Lineage<br>Chromosome location and exon count | Fe-ADH family (conserved Domain Database)<br>Protein accesion number<br>(Gene locus)<br>Amino acid sequence lenght |
|---|---|
| *Ostreococcus tauri*<br>Eukaryota; Viridiplantae; Chlorophyta; Mamiellophyceae; Mamiellales; Ostreococcus<br>Chromosome 5, exon count: 1(XP_003059518) | CD08183<br>XP_003079531-CEF98118<br>Ot05g04340<br>432 aa (corrected) |
| *Ostreococcus lucimarinus* CCE9901<br>Eukaryota; Viridiplantae; Chlorophyta; Mamiellophyceae; Mamiellales; Ostreococcus<br>Chromosome 5, exon count: 1(XP_001417847) | CD08183<br>XP_001417847<br>OSTLU_24600<br>413 aa |
| *Bathycoccus prasinos*<br>Eukaryota; Viridiplantae; Chlorophyta; prasinophytes; Mamiellophyceae; Mamiellales; Bathycoccaceae; Bathycoccus<br>Chromosome 3, exon count: 1(XP_007513902) | CD08183<br>XP_007513902<br>CCO15339<br>(Bathy03g05010)<br>408 aa |
| Streptophyta | |
| *Posidonia oceanica*<br>Eukaryota; Viridiplantae; Streptophyta; Embryophyta; Tracheophyta; Spermatophyta; Magnoliophyta; Liliopsida; Posidoniaceae; Posidonia. | CD08193<br>AEP33255<br>--<br>200 aa (fragment) |
| Other eukaryotes | |
| Alveolata | |
| *Cryptosporidium parvum* Iowa II<br>Eukaryota; Alveolata; Apicomplexa; Conoidasida; Coccidia; Eucoccidiorida; Eimeriorina; Cryptosporidiidae; Cryptosporidium<br>Chromosome 8, exon count: 1 (XP_627078)<br>Chromosome 8, exon count: 1 (XP_627076) | CD08178<br>XP_627078<br>(cgd8_1720)<br>867 aa (bidomain protein)<br><br>CD08178<br>XP_627076<br>(cgd8_1700)<br>480 aa (monodomain protein) |
| *Cryptosporidium hominis* TU502<br>Eukaryota; Alveolata; Apicomplexa; Conoidasida; Coccidia; Eucoccidiorida; Eimeriorina; Cryptosporidiidae; Cryptosporidium<br>Chromosome 8, exon count: 1 (XP_668075)<br>Chromosome 8, exon count: 1 (XP_668076) | CD08178<br>XP_668075<br>(Chro.80199)<br>822 aa (bidomain protein)<br><br>CD08178<br>XP_668076<br>(Chro.80198)<br>469 aa (monodomain protein) |
| *Cryptosporidium muris* RN66<br>Eukaryota; Alveolata; Apicomplexa; Conoidasida; Coccidia; Eucoccidiorida; Eimeriorina; Cryptosporidiidae; Cryptosporidium<br>Chromosome Unknown, exon count: 2 (XP_002139908)<br>Chromosome Unknown, exon count: 1 (XP_002139907) | CD08178<br>XP_002139908<br>(CMU_025660)<br>894 aa (bidomain protein)<br><br>CD08178<br>XP_002139907<br>(CMU_025650)<br>460 aa (monodomain protein) |
| *Karenia brevis*<br>Eukaryota; Alveolata; Dinophyceae; Gymnodiniales; Kareniaceae; Karenia | CD08194<br>ABV49393<br><br>379 aa (fragment) |
| *Vitrella brassicaformis* CCMP3155<br>Eukaryota; Alveolata; Chromerida; Vitrella.<br>Chromosome Unknown, exon count: 18 (CEL99899)<br>Chromosome Unknown, exon count: 14 (CEM34088)<br>Chromosome Unknown, exon count: 20 (CEM19686) | CD08178<br>CEL99899<br>(Vbra_4060)<br>890 aa (bidomain protein)<br><br>Protein family not determined<br>CEM34088<br>(Vbra_10312)<br>930 aa (N-terminal has no putative conserved domain)<br><br>CD08183<br>CEM19686<br>Vbra_5980<br>691 aa (threedomain protein; antibiotic biosynthesis monooxygenase)) |
| Amoebozoa | |
| *Acanthamoeba castellanii* str. Neff<br>Eukaryota; Amoebozoa; Discosea; Longamoebia; Centramoebida; Acanthamoebidae; Acanthamoeba<br>Chromosome Unknown, exon count: Unknown (sequenced from mRNA) | cd08190<br>XP_004347150<br>(ACA1_385440)<br>581 aa |

| Organism[1]<br>Lineage<br>Chromosome location and exon count | Fe-ADH family (conserved Domain Database)<br>Protein accesion number<br>(Gene locus)<br>Amino acid sequence lenght |
|---|---|
| *Mastigamoeba balamuthi*<br>Eukaryota; Amoebozoa; Archamoebae; Mastigamoebidae; Mastigamoeba.<br>Chromosome Unknown, exon count: 3 | CD08178<br>AAM51642<br>(aadhE1)<br>882 aa (bidomain protein) |
| *Entamoeba dispar* SAW760<br>Eukaryota; Amoebozoa; Archamoebae; Entamoebidae; Entamoeba<br>Chromosome Unknown, exon count: 1 (XP_001735062)<br>Chromosome Unknown, exon count: 1 (XP_001741571) | CD08178<br>XP_001735062<br>EDR28755<br>(EDI_342050)<br>870 aa (bidomain protein)<br><br>CD08178<br>XP_001741571<br>(EDI_272710)<br>407 aa (monodomain protein) |
| *Entamoeba histolytica* HM-1:IMSS<br>Eukaryota; Amoebozoa; Archamoebae; Entamoebidae; Entamoeba<br>Chromosome Unknown, exon count: 1 (XP_652300)<br>Chromosome Unknown, exon count: 3 (XP_001913653)<br>Chromosome Unknown, exon count: 1 (XP_655817)<br>Chromosome Unknown, exon count: 1 (XP_652262) | CD08178<br>XP_652300<br>CAA54388<br>Q24803<br>(EHI_150490)<br>870 aa (bidomain protein)<br><br>CD08178<br>XP_001913653<br>(EHI_024240)<br>829 aa<br><br>CD08178<br>XP_655817<br>(EHI_160940)<br>870 aa (bidomain protein)<br><br>CD08178<br>XP_652262<br>(EHI_166490)<br>419 aa (monodomain protein) |
| *Entamoeba histolytica* HM-3:IMSS<br>Eukaryota; Amoebozoa; Archamoebae; Entamoebidae; Entamoeba<br>Chromosome Unknown, exon count: 1 (EMS13409)<br>Chromosome Unknown, exon count: 1 (EMS13752) | CD08178<br>EMS13409<br>(KM1_283380)<br>870 aa (bidomain protein; 447-870 aa)<br><br>CD08178<br>EMS13752<br>(KM1_148280)<br>419 aa |
| *Entamoeba histolytica* KU27<br>Eukaryota; Amoebozoa; Archamoebae; Entamoebidae; Entamoeba<br>Chromosome Unknown, exon count: 3 (EMD44696)<br>Chromosome Unknown, exon count: 1 (EMD48458) | CD08178<br>EMD44696<br>(EHI5A_157720)<br>829 aa<br><br>CD08178<br>EMD48458<br>(EHI5A_016300)<br>419 aa |
| *Entamoeba moshkovskii*<br>Eukaryota; Amoebozoa; Archamoebae; Entamoebidae; Entamoeba<br>Chromosome Unknown, exon count: 1 | CD08178<br>CAI11396<br>(adhE)<br>869 aa |
| *Entamoeba nuttalli* P19<br>Eukaryota; Amoebozoa; Archamoebae; Entamoebidae; Entamoeba<br>Chromosome Unknown, exon count: 1 (XP_008855382)<br>Chromosome Unknown, exon count: 1 (EKE40879) | CD08178<br>XP_008855382<br>EKE42282<br>(ENU1_026400)<br>302 aa (monodomain protein; fragment)<br><br>CD08187<br>XP_008856787<br>EKE40879<br>(ENU1_076680)<br>382 aa |

| Organism[1]<br>Lineage<br>Chromosome location and exon count | Fe-ADH family (conserved Domain Database)<br>Protein accesion number<br>(Gene locus)<br>Amino acid sequence lenght |
|---|---|
| *Entamoeba invadens* IP1<br>Eukaryota; Amoebozoa; Archamoebae; Entamoebidae; Entamoeba<br>Chromosome Unknown, exon count: 1 (XP_004261410)<br>Chromosome Unknown, exon count: 1 (XP_004261373)<br>Chromosome Unknown, exon count: 1 (XP_004184603)<br>Chromosome Unknown, exon count: 2 (XP_004260764)<br>Chromosome Unknown, exon count: 2 (XP_004254345)<br>Chromosome Unknown, exon count: 2 (XP_004183907) | CD08178<br>XP_004261410<br>ELP94639<br>CAI11395<br>(EIN_498390)<br>870 aa (bidomain protein)<br><br>CD08178<br>XP_004261373<br>ELP94602<br>(EIN_497720)<br>867 aa (bidomain protein)<br><br>CD08178<br>XP_004184603<br>(EIN_084190)<br>785 aa (bidomain protein)<br><br>CD08178<br>XP_004260764<br>(EIN_181600)<br>850 aa (bidomain protein)<br><br>CD08178<br>XP_004254345<br>(EIN_215620)<br>337 aa (monodomain protein)<br><br>CD08178<br>XP_004183907<br>(EIN_171060)<br>407 aa (monodomain protein) |
| *Entamoeba terrapinae*<br>Eukaryota; Amoebozoa; Archamoebae; Entamoebidae; Entamoeba<br>Chromosome Unknown, exon count: 1 | CD08178<br>CAI11397<br>(adhE)<br>619 aa (fragment) |
| *Acytostelium subglobosum* LB1<br>Eukaryota; Amoebozoa; Mycetozoa; Dictyosteliida; Acytostelium<br>Chromosome Unknown, exon count: 3 | cd08190<br>XP_012750340<br>(SAMD00019534_098540)<br>546 aa |
| *Polysphondylium pallidum* PN500<br>Eukaryota; Amoebozoa; Mycetozoa; Dictyosteliida; Polysphondylium<br>Chromosome Unknown, exon count: 4 | cd08190<br>EFA77378<br>(PPL_12591)<br>592 aa |
| *Dictyostelium fasciculatum*<br>Eukaryota; Amoebozoa; Mycetozoa; Dictyosteliida; Dictyostelium<br>Chromosome Unknown, exon count: 4 | cd08190<br>XP_004359563<br>(adhfe1)<br>559 aa |
| *Dictyostelium lacteum*<br>Eukaryota; Amoebozoa; Mycetozoa; Dictyosteliida; Dictyostelium<br>Chromosome Unknown, exon count: 1 | cd08190<br>KYR00037<br>(DLAC_03535)<br>549 aa |
| *Dictyostelium discoideum* AX4<br>Eukaryota; Amoebozoa; Mycetozoa; Dictyosteliida; Dictyostelium<br>Chromosome 5, exon count: 1 | cd08190<br>XP_635889<br>(adhfe1)<br>547 aa |
| *Dictyostelium purpureum*<br>Eukaryota; Amoebozoa; Mycetozoa; Dictyosteliida; Dictyostelium<br>Chromosome Unknown, exon count: 1 | cd08190<br>XP_003294908<br>(DICPUDRAFT_51782)<br>543 aa |
| Apusozoa | |
| *Thecamonas trahens* ATCC 50062<br>Eukaryota; Apusozoa; Apusomonadidae; Thecamonas.<br>Chromosome Unknown, exon count: 2 | cd08190<br>XP_013755751<br>(AMSG_07993)<br>445 aa |
| Cryptophyta | |

| Organism[1]<br>Lineage<br>Chromosome location and exon count | Fe-ADH family (conserved Domain Database)<br>Protein accesion number<br>(Gene locus)<br>Amino acid sequence lenght |
|---|---|
| *Guillardia theta* CCMP2712<br>Eukaryota; Cryptophyta; Pyrenomonadales; Geminigeraceae; Guillardia<br>Chromosome Unknown, exon count: 5 (XP_005838939)<br>Chromosome Unknown, exon count: 3 (XP_005838938) | CD08178<br>XP_005838939<br>EKX51959<br>(GUITHDRAFT_92563)<br>907 aa (bidomain protein)<br><br>CD08178<br>XP_005838938<br>GUITHDRAFT_65579<br>875 aa (bidomain protein) |
| Diplomonadida | |
| *Giardia lamblia* ATCC 50803 (gb-synonym: *Giardia intestinalis* ATCC 50803)<br>Eukaryota; Diplomonadida; Hexamitidae; Giardiinae; Giardia<br>Chromosome Unknown, exon count: 1 (XP_001710238)<br>Chromosome Unknown, exon count: 1 (XP_001704899)<br>Chromosome Unknown, exon count: 1(XP_001706932)<br>Chromosome Unknown, exon count: 1(XP_001705763) | CD08178<br>XP_001710238<br>AAC47539<br>GL50803_93358<br>888 aa (bidomain protein)<br><br>CD08181<br>XP_001704899<br>(GL50803_13350)<br>442 aa<br><br>CD08186<br>XP_001706932<br>(GL50803_3861)<br>408 aa<br><br>CD08186<br>XP_001705763<br>(GL50803_3593)<br>407 aa |
| *Giardia intestinalis* ATCC 50581<br>Eukaryota; Diplomonadida; Hexamitidae; Giardiinae; Giardia<br>Chromosome Unknown, exon count: 1 (CD08178)<br>Chromosome Unknown, exon count: 1 (EET02355)<br>Chromosome Unknown, exon count: 1 (EET00033)<br>Chromosome Unknown, exon count: 1 (EET01185) | CD08178<br>(not registered; 100% identical to ESU43298)<br>(ACGJ01002313: 31285-33261,<br>ACGJ01002195: 2-733)<br>888 aa (bidomain protein; 454-888aa)<br><br>CD08181<br>EET02355<br>(GL50581_396)<br>405 aa (monodomain protein)<br><br>CD08186<br>EET00033<br>(GL50581_2728)<br>408 aa<br><br>CD08186<br>EET01185<br>(GL50581_1580)<br>407 aa |
| *Giardia intestinalis* (*Giardia lamblia*) DH<br>Eukaryota; Diplomonadida; Hexamitidae; Giardiinae; Giardia<br>Chromosome Unknown, exon count: 1 (ESU37410) | CD08178<br>ESU37410<br>(DHA2_93358)<br>888 aa (bidomain protein; 454-888aa)<br><br>CD08181<br>ESU36221<br>(DHA2_13350)<br>443 aa<br><br>CD08186<br>ESU37832<br>(DHA2_3593)<br>407 aa |

| Organism[1]<br>Lineage<br>Chromosome location and exon count | Fe-ADH family (conserved Domain Database)<br>Protein accesion number<br>(Gene locus)<br>Amino acid sequence lenght |
|---|---|
| *Giardia lamblia* P15<br>Eukaryota; Diplomonadida; Hexamitidae; Giardiinae; Giardia<br>Chromosome Unknown, exon count: 1 (EFO64033)<br>Chromosome Unknown, exon count: 1 (EFO62795)<br>Chromosome Unknown, exon count: 1 (EFO63891) | CD08178<br>EFO64033<br>(GLP15_4141)<br>888 aa (bidomain protein; 454-888aa)<br><br>CD08181<br>EFO62795<br>(GLP15_696)<br>406 aa<br><br>CD08186<br>EFO63891<br>(GLP15_2750)<br>407 aa |
| *Giardia intestinalis* (*Giardia lamblia*) GS<br>Eukaryota; Diplomonadida; Hexamitidae; Giardiinae; Giardia<br>Chromosome Unknown, exon count: 1 | CD08178<br>ESU43298<br>GSB_93358<br>888 aa (bidomain protein; 454-888aa) |
| *Giardia intestinalis* assemblage B<br>Eukaryota; Diplomonadida; Hexamitidae; Giardiinae; Giardia<br>Chromosome Unknown, exon count: 1 (KWX15163)<br>Chromosome Unknown, exon count: 1 (KWX15511)<br>Chromosome Unknown, exon count: 1 (KWX15824) | CD08178<br>KWX15163<br>(QR46_0787)<br>888 aa (bidomain protein; 454-888aa)<br><br>CD08181<br>KWX15511<br>(QR46_0496)<br>405 aa<br><br>CD08186<br>KWX15824<br>(QR46_0142)<br>407 aa |
| *Spironucleus barkhanus*<br>Eukaryota; Diplomonadida; Hexamitidae; Hexamitinae; Spironucleus | CD08178<br>AAM94650<br>(adhE)<br>880 aa |
| *Spironucleus salmonicida*<br>Eukaryota; Diplomonadida; Hexamitidae; Hexamitinae; Spironucleus | CD08178<br>AFV80071<br>(adhE)<br>880 aa |
| Euglenozoa | |
| *Angomonas deanei*<br>Eukaryota; Euglenozoa; Kinetoplastida; Trypanosomatidae; Strigomonadinae; Angomonas.<br>Chromosome Unknown, exon count: 1 (EPY39735)<br>Chromosome Unknown, exon count: 1 (EPY43482) | CD08176<br>EPY39735<br>(AGDE_04193)<br>394 aa<br><br>CD08189<br>EPY43482<br>(AGDE_00439)<br>355 aa |
| *Leishmania panamensis* MHOM/PA/94/PSC-1<br>Eukaryota; Euglenozoa; Kinetoplastida; Trypanosomatidae; Leishmaniinae; Leishmania; Leishmania guyanensis species complex<br>Chromosome 30, exon count: 1 | CD08189<br>XP_010701228<br>(LPMP_302050)<br>399 aa |
| *Leishmania guyanensis*<br>Eukaryota; Euglenozoa; Kinetoplastida; Trypanosomatidae; Leishmaniinae; Leishmania; Leishmania guyanensis species complex<br>Chromosome Unknown, exon count: 1 | CD08189<br>CCM25618<br>(BN141_3052210)<br>407 aa |
| *Leishmania braziliensis* complex EV-2015<br>Eukaryota; Euglenozoa; Kinetoplastida; Trypanosomatidae; Leishmaniinae; Leishmania; Leishmania braziliensis species complex | CD08189<br>AKK31263<br><br>326 aa (fragment) |
| *Leishmania braziliensis* MHOM/BR/75/M2904<br>Eukaryota; Euglenozoa; Kinetoplastida; Trypanosomatidae; Leishmaniinae; Leishmania; Leishmania braziliensis species complex<br>Chromosome 30, exon count: 1 | CD08189<br>XP_001566831<br>(LBRM_30_2040)<br>399 aa |
| *Leishmania tropica*<br>Eukaryota; Euglenozoa; Kinetoplastida; Trypanosomatidae; Leishmaniinae; Leishmania<br>Chromosome Unknown, exon count: 1 | CD08189<br>AKK31254<br><br>399 aa |
| *Leishmania major* strain Friedlin<br>Eukaryota; Euglenozoa; Kinetoplastida; Trypanosomatidae; Leishmaniinae; Leishmania<br>Chromosome 30, exon count: 1 | CD08189<br>XP_001684814<br>(LMJF_30_2090)<br>399 aa |

| Organism[1]<br>Lineage<br>Chromosome location and exon count | Fe-ADH family (conserved Domain Database)<br>Protein accesion number<br>(Gene locus)<br>Amino acid sequence lenght |
|---|---|
| *Leishmania mexicana* MHOM/GT/2001/U1103<br>Eukaryota; Euglenozoa; Kinetoplastida; Trypanosomatidae; Leishmaniinae; Leishmania<br>Chromosome 29, exon count: 1 | CD08189<br>XP_003877353<br>(LMXM_29_2090)<br>399 aa |
| *Leishmania amazonensis*<br>Eukaryota; Euglenozoa; Kinetoplastida; Trypanosomatidae; Leishmaniinae; Leishmania<br>Chromosome Unknown, exon count: 1 | CD08189<br>AKK31262<br><br>399 aa |
| *Leishmania infantum* JPCM5<br>Eukaryota; Euglenozoa; Kinetoplastida; Trypanosomatidae; Leishmaniinae; Leishmania<br>Chromosome 30, exon count: 1 | CD08189<br>XP_001467057<br>(LINJ_30_2100)<br>399 aa |
| *Leishmania donovani* LV9<br>Eukaryota; Euglenozoa; Kinetoplastida; Trypanosomatidae; Leishmaniinae; Leishmania<br>Chromosome Unknown, exon count: 1 | CD08189<br>AKK31260<br><br>399 aa |
| *Leishmania donovani* BPK282A1<br>Eukaryota; Euglenozoa; Kinetoplastida; Trypanosomatidae; Leishmaniinae; Leishmania<br>Chromosome 30, exon count: 1 | CD08189<br>XP_003862922<br>(LDBPK_302100)<br>399 aa |
| *Leptomonas seymouri*<br>Eukaryota; Euglenozoa; Kinetoplastida; Trypanosomatidae; Leishmaniinae; Leptomonas<br>Chromosome Unknown, exon count: 1 (KPI83770)<br>Chromosome Unknown, exon count: 1 (KPI90171) | CD08176<br>KPI83770<br>(ABL78_7186)<br>393 aa<br><br>CD08189<br>KPI90171<br>(ABL78_0689)<br>407 aa |
| *Leptomonas pyrrhocoris*<br>Eukaryota; Euglenozoa; Kinetoplastida; Trypanosomatidae; Leishmaniinae; Leptomonas<br>Chromosome Unknown, exon count: 1 (XP_015654215)<br>Chromosome Unknown, exon count: 1 (XP_015661690) | CD08176<br>XP_015654215<br>(ABB37_08308)<br>360 aa<br><br>CD08189<br>XP_015661690<br>(ABB37_02930)<br>407 aa |
| *Phytomonas* sp. isolate Hart1<br>Eukaryota; Euglenozoa; Kinetoplastida; Trypanosomatidae; Phytomonas; unclassified Phytomonas<br>Chromosome Unknown, exon count: 1 (CCW71337)<br>Chromosome Unknown, exon count: 1 (CCW68085) | CD08176<br>CCW71337<br>(GSHART1_T00001798001)<br>394 aa<br><br>CD08189<br>CCW68085<br>(GSHART1_T00004766001)<br>390 aa |
| *Phytomonas* sp. isolate EM1<br>Eukaryota; Euglenozoa; Kinetoplastida; Trypanosomatidae; Phytomonas; unclassified Phytomonas<br>Chromosome Unknown, exon count: 1 (CCW64160)<br>Chromosome Unknown, exon count: 1 (CCW61617) | CD08176<br>CCW64160<br>(GSEM1_T00003582001)<br>394 aa<br><br>CD08189<br>CCW61617<br>(GSEM1_T00006475001)<br>407 aa |
| *Strigomonas culicis*<br>Eukaryota; Euglenozoa; Kinetoplastida; Trypanosomatidae; Strigomonadinae; Strigomonas<br>Chromosome Unknown, exon count: 1 | CD08189<br>EPY36875<br>EPY43482<br>(STCU_00363)<br>(STCU_00685)<br>407 aa |
| *Trypanosoma cruzi* strain CL Brener<br>Eukaryota; Euglenozoa; Kinetoplastida; Trypanosomatidae; Trypanosoma; Schizotrypanum<br>Chromosome Unknown, exon count: 1 | CD08176<br>XP_819264<br>(Tc00.1047053506357.50)<br>392 aa |
| *Trypanosoma cruzi marinkellei*<br>Eukaryota; Euglenozoa; Kinetoplastida; Trypanosomatidae; Trypanosoma; Schizotrypanum<br>Chromosome Unknown, exon count: 1 | CD08176<br>EKF31255<br>(MOQ_004912)<br>461 aa |
| Haptophyceae | |

| Organism[1]<br>Lineage<br>Chromosome location and exon count | Fe-ADH family (conserved Domain Database)<br>Protein accesion number<br>(Gene locus)<br>Amino acid sequence lenght |
|---|---|
| *Emiliania huxleyi* CCMP1516<br>Eukaryota; Haptophyceae; Isochrysidales; Noelaerhabdaceae; Emiliania<br>Chromosome Unknown, exon count: 3 (XP_005761545)<br>Chromosome Unknown, exon count: 8 (XP_005769875)<br>Chromosome Unknown, exon count: 3 (XP_005763823)<br>Chromosome Unknown, exon count: 5 (XP_005789285) | CD08177<br>EOD09116<br>XP_005761545<br>(EMIHUDRAFT_216985)<br>429 aa<br><br>CD08193<br>XP_005769875-XP_005769573<br>(EMIHUDRAFT_451417)<br>396 aa (corrected)<br><br>CD08194<br>XP_005763823<br>XP_005765505<br>(EMIHUDRAFT_357847)<br>401 aa<br><br>CD08192<br>XP_005789285<br>EOD36856<br>(EMIHUDRAFT_415021)<br>417 aa |
| Heterolobosea | |
| *Naegleria gruberi* NEG-M<br>Eukaryota; Heterolobosea; Schizopyrenida; Vahlkampfiidae; Naegleria<br>Chromosome Unknown, exon count: 2 (XP_002681118) | CD08176<br>XP_002681118<br>CAI28407<br>(NAEGRDRAFT_56035)<br>453 aa |
| Ichthyosporea | |
| *Capsaspora owczarzaki* ATCC 30864<br>Eukaryota; Ichthyosporea; Capsaspora.<br>Chromosome Unknown, exon count: 8 (XP_004344325) | cd08190<br>XP_004344325<br>EFW41572<br>KJE96366<br>(CAOG_06704)<br>560 aa |
| *Sphaeroforma arctica* JP610<br>Eukaryota; Ichthyosporea; Ichthyophonida; Sphaeroforma<br>Chromosome Unknown, exon count: 5 | CD08194<br>XP_014157820<br>(SARC_03853)<br>227 aa (fragment) |
| Parabasalidea | |

| Organism[1]<br>Lineage<br>Chromosome location and exon count | Fe-ADH family (conserved Domain Database)<br>Protein accesion number<br>(Gene locus)<br>Amino acid sequence lenght |
|---|---|
| *Trichomonas vaginalis* G3<br>Eukaryota; Parabasalidea; Trichomonada; Trichomonadida; Trichomonadidae; Trichomonadinae; Trichomonas<br>Chromosome Unknown, exon count: 1 (XP_001308988)<br>Chromosome Unknown, exon count: 1 (XP_001319933)<br>Chromosome Unknown, exon count: 1 (XP_001322301)<br>Chromosome Unknown, exon count: 1 (XP_001322302)<br>Chromosome Unknown, exon count: 1 (XP_001315345)<br>Chromosome Unknown, exon count: 1 (XP_001322724)<br>Chromosome Unknown, exon count: 1 (XP_001307170) | CD08187<br>XP_001308988<br>(TVAG_265470)<br>200 aa (probable pseudogene)<br><br>CD08187<br>XP_001319933<br>(TVAG_117910)<br>392 aa<br><br>CD08187<br>XP_001322301<br>TVAG_329650<br>394 aa<br><br>CD08187<br>XP_001322302<br>TVAG_329660<br>257 aa<br><br>CD08187<br>XP_001315345<br>TVAG_328940<br>395 aa<br><br>CD08187<br>XP_001322724<br>(TVAG_484120)<br>393 aa<br><br>CD08187<br>XP_001307170<br>(TVAG_001570)<br>389 aa |
| Rhizaria | |
| *Plasmodiophora brassicae*<br>Eukaryota; Rhizaria; Cercozoa; Plasmodiophorida; Plasmodiophoridae; Plasmodiophora<br>Chromosome Unknown, exon count: 7 | CD08194<br>CEP00319<br>(PBRA_008053)<br>388 aa |
| *Reticulomyxa filosa*<br>Eukaryota; Rhizaria; Foraminifera; Monothalamids; Reticulomyxidae; Reticulomyxa<br>Chromosome Unknown, exon count: 2 | cd08190<br>ETO27853<br>(RFI_09279)<br>483 aa |
| Rhodophyta | |
| *Galdieria sulphuraria* 074W<br>Eukaryota; Rhodophyta; Bangiophyceae; Cyanidiales; Cyanidiaceae; Galdieria<br>Chromosome Unknown, exon count: 4 | CD08183<br>XP_005703706<br>(Gasu_52870)<br>701 aa (bidomain protein; 280-701 aa) |
| *Chondrus crispus* (carragheen)<br>Eukaryota; Rhodophyta; Florideophyceae; Gigartinales; Gigartinaceae; Chondrus<br>Chromosome Unknown, exon count: 2 | CD08183<br>CDF36599<br>(CHC_T00004859001)<br>500 aa |
| Stramenopiles | |
| *Blastocystis hominis* (isolate: Singapore isolate B (sub-type 7))<br>Eukaryota; Stramenopiles; Blastocystis<br>Chromosome Unknown, exon count: 4 | CD08187<br>CBK25146<br>(GSBLH_T00004779001)<br>398 aa |
| *Aphanomyces astaci* APO3<br>Eukaryota; Stramenopiles; Oomycetes; Saprolegniales; Saprolegniaceae; Aphanomyces<br>Chromosome Unknown, exon count: 7 | cd08190<br>XP_009837382<br>(H257_11991)<br>444 aa |
| *Aphanomyces invadans* NJM9701<br>Eukaryota; Stramenopiles; Oomycetes; Saprolegniales; Saprolegniaceae; Aphanomyces<br>Chromosome Unknown, exon count: 5 | cd08190<br>XP_008876342<br>(H310_11432)<br>379 aa |
| *Phytophthora infestans* T30-4<br>Eukaryota; Stramenopiles; Oomycetes; Peronosporales; Phytophthora<br>Chromosome Unknown, exon count: 2 | CD08183<br>XP_002904000<br>EEY55055<br>(PITG_08643)<br>434 aa (monodomain protein) |

| Organism[1]<br>Lineage<br>Chromosome location and exon count | Fe-ADH family (conserved Domain Database)<br>Protein accesion number<br>(Gene locus)<br>Amino acid sequence lenght |
|---|---|
| *Phytophthora sojae* (strain: P6497)<br>Eukaryota; Stramenopiles; Oomycetes; Peronosporales; Phytophthora<br>Chromosome Unknown, exon count: 2 | CD08183<br>XP_009524672<br>EGZ21955<br>(PHYSODRAFT_542747)<br>423 aa (monodomain protein) |
| *Phytophthora parasitica* INRA-310<br>Eukaryota; Stramenopiles; Oomycetes; Peronosporales; Phytophthora<br>Chromosome Unknown, exon count: 2 (XP_008892526) | CD08183<br>XP_008892526<br>ETN23353<br>(PPTG_02942)<br>476 aa (monodomain protein) |
| *Phytophthora parasitica* P10297<br>Eukaryota; Stramenopiles; Oomycetes; Peronosporales; Phytophthora<br>Chromosome Unknown, exon count: 2 | CD08183<br>ETP46926<br>(F442_06909)<br>452 aa |
| *Phytophthora parasitica* P1569<br>Eukaryota; Stramenopiles; Oomycetes; Peronosporales; Phytophthora<br>Chromosome Unknown, exon count: 2 | CD08183<br>ETI49210<br>(F443_06870)<br>476 aa |
| *Phytophthora parasitica* P1976<br>Eukaryota; Stramenopiles; Oomycetes; Peronosporales; Phytophthora<br>Chromosome Unknown, exon count: 2 | CD08183<br>ETO77947<br>(F444_06937)<br>476 aa |
| *Phytophthora parasitica* CJ02B3<br>Eukaryota; Stramenopiles; Oomycetes; Peronosporales; Phytophthora<br>Chromosome Unknown, exon count: 2 | CD08183<br>ETK89106<br>(L915_06742)<br>476 aa |
| *Plasmopara halstedii*<br>Eukaryota; Stramenopiles; Oomycetes; Peronosporales; Peronosporaceae; Plasmopara<br>Chromosome Unknown, exon count: 2 | CD08183<br>CEG41588<br>(PHALS_11924)<br>437 aa |
| *Ectocarpus siliculosus*<br>Eukaryota; Stramenopiles; PX clade; Phaeophyceae; Ectocarpales; Ectocarpaceae; Ectocarpus.<br>Chromosome Unknown, exon count: 7 (CBJ29353)<br>Chromosome Unknown, exon count: 8 (CBJ30835) | CD08183Unknown<br>CBJ29353<br>(Esi_0144_0006)<br>428 aa<br><br>CD08183Unknown<br>CBJ30835<br>(Esi_0217_0010)<br>325 aa (fragment) |
| *Nannochloropsis gaditana*<br>Eukaryota; Stramenopiles; Eustigmatophyceae; Eustigmatales; Monodopsidaceae; Nannochloropsis.<br>Chromosome Unknown, exon count: 2 (EWM25837)<br>Chromosome Unknown, exon count: 5 (EWM27010)<br>Chromosome Unknown, exon count: 1 (EWM28042) | CD08177<br>EWM25837<br>(Naga_100021g28)<br>637 aa (bidomain protein)<br>1-415aa (Fe-ADH domain)<br><br>CD08182<br>EWM27010<br>(Naga_100079g15)<br>509 aa<br><br>CD08183<br>EWM28042<br>(Naga_100008g93)<br>449 aa |
| *Saprolegnia diclina* VS20<br>Eukaryota; Stramenopiles; Oomycetes; Saprolegniales; Saprolegniaceae; Saprolegnia<br>Chromosome Unknown, exon count: 2 | cd08190<br>XP_008614861<br>(SDRG_10638)<br>454 aa |
| *Saprolegnia parasitica* CBS 223.65<br>Eukaryota; Stramenopiles; Oomycetes; Saprolegniales; Saprolegniaceae; Saprolegnia<br>Chromosome Unknown, exon count: 2 | cd08190<br>XP_012208377<br>(SPRG_14119)<br>454 aa |
| *Phaeodactylum tricornutum* CCAP 1055/1<br>Eukaryota; Stramenopiles; Bacillariophyta; Bacillariophyceae; Bacillariophycidae; Naviculales; Phaeodactylaceae; Phaeodactylum<br>Chromosome 6, exon count: 1 | cd08190<br>XP_002179196<br>(PHATRDRAFT_19659)<br>465 aa |
| *Thalassiosira pseudonana* CCMP1335<br>Eukaryota; Stramenopiles; Bacillariophyta; Coscinodiscophyceae; Thalassiosirophycidae; Thalassiosirales; Thalassiosiraceae; Thalassiosira<br>Chromosome 14, exon count: 1 (XP_002293824) | CD08177<br>XP_002293824<br>(THAPSDRAFT_24928)<br>418 aa |

| Organism[1]<br>Lineage<br>Chromosome location and exon count | Fe-ADH family (conserved Domain Database)<br>Protein accesion number<br>(Gene locus)<br>Amino acid sequence lenght |
|---|---|
| *Thalassiosira oceanica*<br>Eukaryota; Stramenopiles; Bacillariophyta; Coscinodiscophyceae; Thalassiosirophycidae; Thalassiosirales; Thalassiosiraceae; Thalassiosira<br>Chromosome Unknown, exon count: 2 (EJK70579) | cd08190<br>EJK70579<br>(THAOC_08052)<br>488 aa |
| *Aureococcus anophagefferens*<br>Eukaryota; Stramenopiles; Pelagophyceae; Pelagomonadales; Aureococcus<br>Chromosome Unknown, exon count: 2 | CD08192<br>XP_009041394<br>(AURANDRAFT_33332)<br>236 aa (fragment) |
